# Carbohydrate-auxiliary assisted preparation of enantiopure 1,2-oxazine derivatives and aminopolyols

**DOI:** 10.3762/bjoc.8.74

**Published:** 2012-04-30

**Authors:** Marcin Jasiński, Dieter Lentz, Hans-Ulrich Reissig

**Affiliations:** 1 Institut für Chemie und Biochemie, Freie Universität Berlin, Takustr. 3, D-14195 Berlin, Germany; 2 Department of Organic and Applied Chemistry, University of Łódź, Tamka 12, PL-91-403 Poland

**Keywords:** aminopolyols, carbohydrates, chiral auxiliaries, lithiated alkoxyallenes, 1,2-oxazines, pyrroles, pyrrolidines, samarium diiodide

## Abstract

An approach to enantiopure hydroxylated 2*H*-1,2-oxazine derivatives is presented utilizing the [3 + 3] cyclisation of lithiated alkoxyallenes and an L-erythrose-derived *N*-glycosyl nitrone as precursors. This key step proceeded only with moderate diastereoselectivity, but allowed entry into both enantiomeric series of the resulting 3,6-dihydro-2*H*-1,2-oxazines. Their enol ether double bond was then subjected to a hydroboration followed by an oxidative work-up, and finally the auxiliary was removed. The described three-step procedure enabled the synthesis of enantiopure hydroxylated 1,2-oxazines. Typical examples were treated with samarium diiodide leading to enantiopure acyclic aminopolyols. We also report on our attempts to convert these compounds into enantiopure hydroxylated pyrrolidine derivatives.

## Introduction

**Scheme 1:**
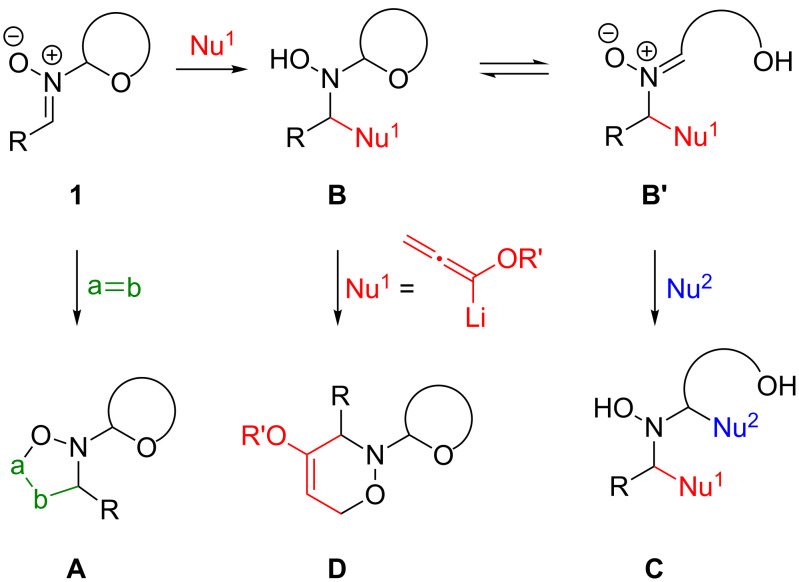
Reactivity of *N*-glycosyl nitrones **1** towards dipolarophiles and nucleophiles leading to products of type **A**, **B**, **C** and **D**.

During the last few decades carbohydrate-derived nitrones have turned out to be particularly attractive tools for the synthesis of structurally complex compounds [1–4]. Employed mainly as 1,3-dipoles in cycloadditions [5–6] or as imine analogues in nucleophilic additions [7–8], these nitrones very often furnish the corresponding products in a highly selective manner. In this context, reactions of lithiated alkoxyallenes with enantiopure nitrones are particularly of interest since they lead by a [3 + 3] cyclisation process to 1,2-oxazine derivatives with excellent diastereoselectivity [9]. We previously reported on the unusually diverse synthetic potential of carbohydrate-derived 1,2-oxazines allowing the smooth and flexible preparation of various highly functionalised compounds, including de novo syntheses of carbohydrates and their mimetics, as well as *N*-heterocycles [10–12]. Although the reactions of lithiated alkoxyallenes, with nitrones bearing substituents with stereogenic centres at the carbon atom, were studied in our group in great detail [13], *N*-glycosyl-substituted nitrones have so far not been used as electrophiles for this purpose. This type of nitrone has been introduced and broadly studied by Vasella and co-workers [14–20] and has also been used by other groups [21–25]. They observed moderate to high diastereoselectivities for 1,3-dipolar cycloadditions and for nucleophilic additions. Successful applications of these easily removable auxiliaries in the syntheses of biologically active agents were also reported [26–31]. Apart from the obvious reactivity of *N*-glycosyl nitrones of type **1** leading to five-membered heterocycles **A** or to *N*,*N*-disubstituted hydroxylamine derivatives **B**, a twofold nucleophilic addition of an excess of organometallic reagents furnishing compounds of type **C** (Nu^1^ = Nu^2^) was described and discussed by Goti et al. (Scheme 1) [32]. In selected examples, the synthesis of differently substituted products (Nu^1^ ≠ Nu^2^) was possible by consecutive additions of the appropriate Grignard reagents [33]. Here we report on the application of a nitrone with an L-erythronolactone-derived auxiliary for the synthesis of 3,6-dihydro-2*H*-1,2-oxazine derivatives of type **D**. Their selected transformations, including hydroboration of the enol ether moiety, oxidative work-up, glycosyl cleavage, and samarium diiodide-induced reactions, are presented as well.

## Results and Discussion

**Scheme 2:**
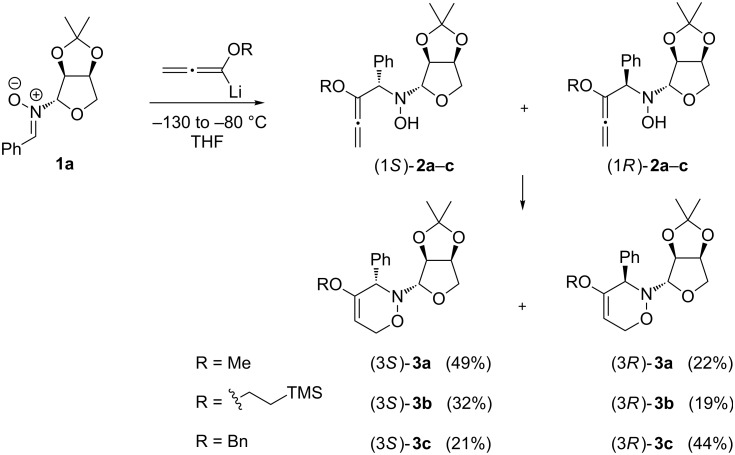
Additions of lithiated alkoxyallenes to L-erythrose-derived nitrone **1a** leading to 3,6-dihydro-2*H*-1,2-oxazine derivatives **3** via the respective *N*-hydroxylamines **2**.

**Figure 1:**
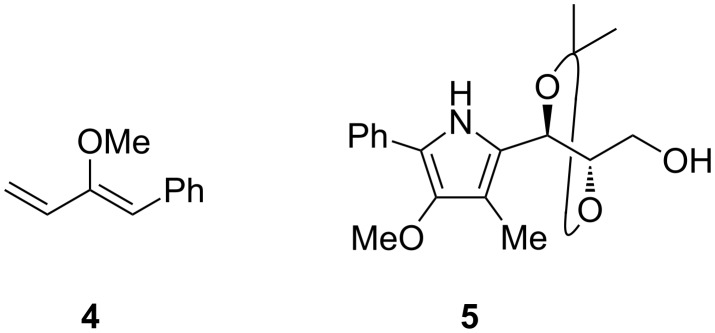
By-products **4** and **5** isolated from the reaction of nitrone **1a** with lithiated methoxyallene.

In continuation of our recent exploration of L-erythrose-derived nitrones for the synthesis of 3,6-dihydro-2*H*-1,2-oxazine derivatives [34], we turned our attention to benzaldehyde-derived nitrone **1a**, which is readily available in a three-step procedure starting from L-arabinose. L-Erythronolactone was first prepared [35] and was subsequently treated with *N*-benzylhydroxylamine [36], and the resulting product was oxidised with activated MnO_2_ [37] to furnish the desired compound **1a** in 51% overall yield. The initial experiment with **1a** was carried out under typical conditions with 2.4 equiv of lithiated methoxyallene at −78 °C in THF. Similarly to previous results for more rigid cyclic nitrones [38], the formation of the intermediate *N*-hydroxylamines **2** (Scheme 2) was clearly observed. These primarily formed compounds were not isolated, but (in the presence of a drying agent) they underwent slow cyclisation in Et_2_O solution at room temperature to furnish the desired 1,2-oxazine derivative as a mixture of separable diastereomers (3*S*)-**3a** and (3*R*)-**3a** in 25% and 8% yield, respectively (Table 1, entry 3). The 1,2-oxazines were accompanied by a complex mixture of by-products, from which only two compounds **4** (1%) and **5** (3%) could be isolated in pure form (Figure 1). After tedious optimisation with respect to stoichiometry, temperature, time, concentration, etc. (selected results are presented in Table 1), we found that running the reaction from −130 to −80 °C, followed by standing overnight at room temperature, allowed the synthesis of **3a** with an overall yield of 75% and a ratio of diastereomers of ca. 2:1 (49% and 22% after separation of the isomers, Table 1, entry 7). When the reaction was scaled up to 3.50 g of **1a** the expected diastereomers of 1,2-oxazines **3a** were obtained with no decrease in yield (78%). As illustrated in Scheme 2, lithiated (2-trimethylsilyl)ethoxyallene and benzyloxyallene were also examined under the optimised reaction conditions and furnished the expected diastereomers of 1,2-oxazine derivatives **3b** and **3c** in 51% and 65% yield, respectively.

**Table 1:** Selected reaction conditions of nitrone **1a** with lithiated methoxyallene.

entry	lithiated methoxyallene (equiv)^a^	*T* (°C)	time (h)	(3*S*)-**3a** / (3*R*)-**3a** ratio^b^	yield^c^ [%]

1	10.0	−78	1.0	–	traces^d^
2	3.0	−78	1.0	3:1	26
3	2.4	−78	1.0	3:1	33
4	2.2	−78	1.0	3:1	38
5	1.5	−78	1.0	2.5:1	26
6	2.2	−100 → −80	1.0	2:1	51
7	2.2	−130 → −80	1.5	2:1	75

^a^Reactions performed in 1.1 mmol scale with respect to **1a**. ^b^Crude product. ^c^Combined yield of isolated (3*S*)-**3a** and (3*R*)-**3a**. ^d^Only the major diastereomer (3*S*)-**3a** was detected.

**Figure 2:**
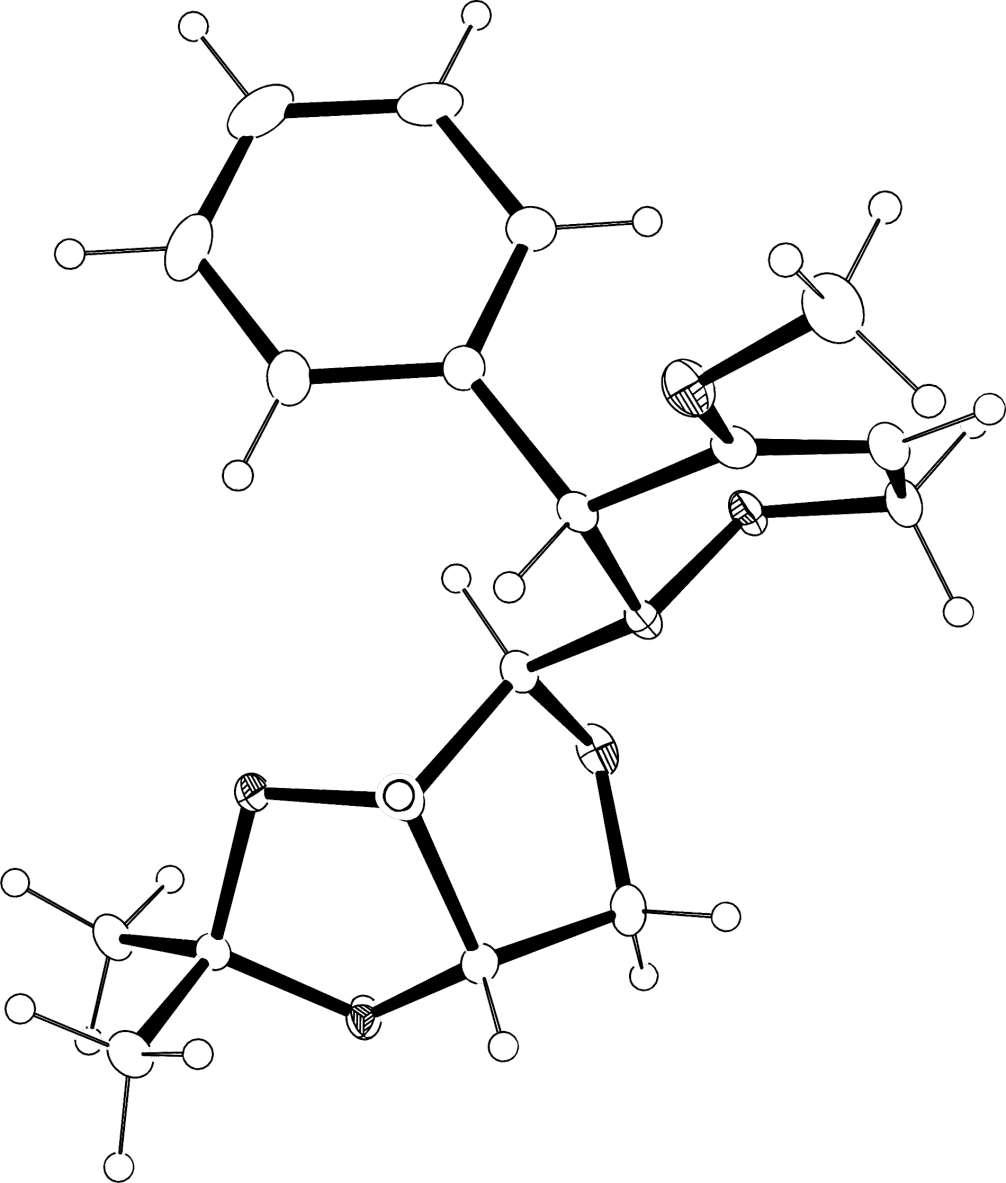
Single-crystal X-ray analysis of (3*R*)-**3a** (ellipsoids are drawn at a 50% probability level).

The mixtures of diastereomers of crude 1,2-oxazines **3a**–**c** were easily separated by standard column chromatography and characterised by spectroscopic methods. However, in certain cases additional purification was necessary to obtain analytically pure samples. The absolute configuration of the newly generated stereogenic centre could not be determined based on NMR techniques. For instance, in the ^1^H NMR spectra of the diastereomeric products (3*S*)-**3a** and (3*R*)-**3a**, the signals of the benzylic protons assigned to C-3 of the 1,2-oxazine ring appear as singlets at 4.85 and 4.48 ppm, respectively. Due to the unhindered rotation of the auxiliary moiety similar correlation peaks in NOE experiments were observed for both isomers. Fortunately, the minor product (3*R*)-**3a** isolated as an amorphous solid could be recrystallised from ethyl acetate solution to give crystals suitable for an X-ray crystallographic analysis (Figure 2). The X-ray analysis of (3*R*)-**3a** shows a well-defined half-chair conformation of the 1,2-oxazine ring, with four carbon atoms in plane and with ONCC and OCCC torsion angles of −60° and 13°, respectively. The bulky *N*-substituent occupies a pseudo-equatorial position and the phenyl group is in a pseudo-axial position. Since characteristic shift patterns in the ^1^H NMR spectra for both diastereomeric series are observed, the configuration at C-3 of the TMSE derivatives (**b**) and benzyloxy analogues (**c**) could be assigned as well with high certainty.

**Figure 3:**
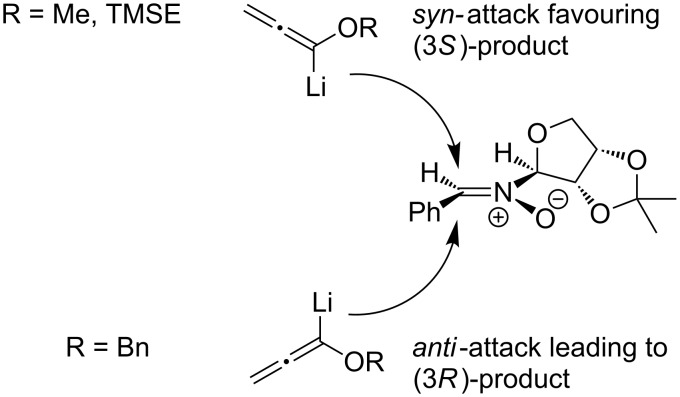
Model proposed for the addition of lithiated allenes to nitrone **1a**.

The stereochemical outcome observed for the reactions studied also deserves some comment. Whereas lithiated methoxy- and TMS-ethoxyallene yielded the diastereomers in ca. 2:1 ratio, in the case of lithiated benzyloxyallene a significant switch of the selectivity to an approximate 1:2 ratio was observed. According to the model proposed in the literature [7,18,20] for the addition of nucleophiles, the stereochemical course is governed by a competition of steric and electronic effects. As presented in Figure 3, the bulky benzyloxy substituent favours the *anti*-addition and hence yields the (3*R*)-configured product as the major compound. In contrast, the less hindered lithiated methoxyallene enables a *syn*-attack supported by a “kinetic anomeric effect”, stabilizing the respective transition state [20] and furnishing the (3*S*)-configured 1,2-oxazine. In all tested examples the level of diastereoselectivity was only low to moderate, and the interpretation should therefore not be exaggerated. Alternative conformations explaining the results are certainly possible.

The fragmentation of the primarily formed allenyl *N*-hydroxylamines of type **2** leading to 1,3-dienes such as **4** (Figure 1) by retro-nitroso-ene reaction was discussed in earlier work [13], but the formation of pyrrole derivative **5** is unprecedented for the reactions of nitrones and lithiated alkoxyallenes. The ^1^H NMR spectrum of **5** shows four singlets (3H each) assigned to three methyl groups and one methoxy substituent. Additionally, a broad singlet at δ = 8.98 ppm attributed to the NH functionality, an OH group at 2.11 ppm (dd, *J* ≈ 4.0, 8.1 Hz) coupling with the adjacent methylene group, and a characteristic set of multiplets of the [1,3]dioxolane and the phenyl moieties could be found. The signals of four quaternary carbon atoms in the ^13^C NMR spectrum evidenced the presence of the pyrrole structure. Finally, the HMBC experiment proved the proposed substitution pattern at the pyrrole ring. HRMS and elemental analysis allowed identification of **5** as a 2,3,4,5-tetrasubstituted pyrrole derivative.

**Scheme 3:**
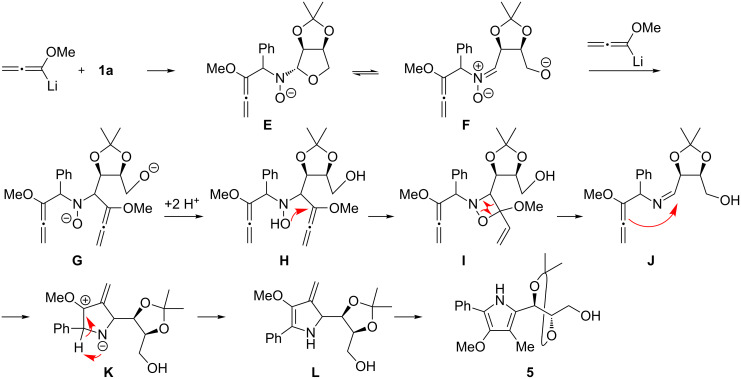
Speculative mechanistic suggestion for the formation of tetrasubstituted pyrrole derivative **5**.

As shown in Table 1, entries 1–3, a higher excess of lithiated methoxyallene resulted in a significant decrease in the yield of 1,2-oxazine derivatives. For example, in the case of 10 equiv of lithiated methoxyallene (Table 1, entry 1) only a trace amount of (3*S*)-**3a** (<5%) and numerous side products were found in the crude product, including compound **5**. On the other hand, no pyrrole **5** could be detected when only a slight excess of methoxyallene was used (Table 1, entry 5) or when the reaction was performed at lower temperatures (Table 1, entries 6 and 7). These observations prompted us to postulate that the surprising formation of **5** is the result of a double addition of lithiated methoxyallene to nitrone **1a** as illustrated in Scheme 3, the crucial step being the (reversible) opening of the tetrahydrofuran ring of the primary addition product **E**. The resulting new nitrone **F** can then react with the second equiv of lithiated methoxyallene to give the double adduct **G**. After aqueous work-up, hydroxylamine derivative **H** underwent a ring-closure to 1,2-oxazetidine derivative **I**. It is known that this class of compounds can suffer a thermally induced [2 + 2] cycloreversion involving N–O bond cleavage [39–40], which, in our case, led to the formation of methyl acrylate and imine **J**. This imine underwent subsequent cyclisation to zwitterion **K** and two proton shifts, probably via 3-exomethylene compound **L,** finally led to pyrrole **5**. This mechanism is certainly speculative but offers a possibility to explain the formation of the tetrasubstituted pyrrole **5**. The structure of the intermediate double-addition product **H** suggests that other by-products may be formed, e.g., two different *N*-allenylmethyl-substituted 3,6-dihydro-2*H*-1,2-oxazines or an isomeric pyrrole derivative. The numerous sets of signals in the ^1^H NMR spectrum as well as additional spots seen on TLC of the crude reaction mixtures support this assumption, but none of these possible by-products could be isolated.

**Scheme 4:**
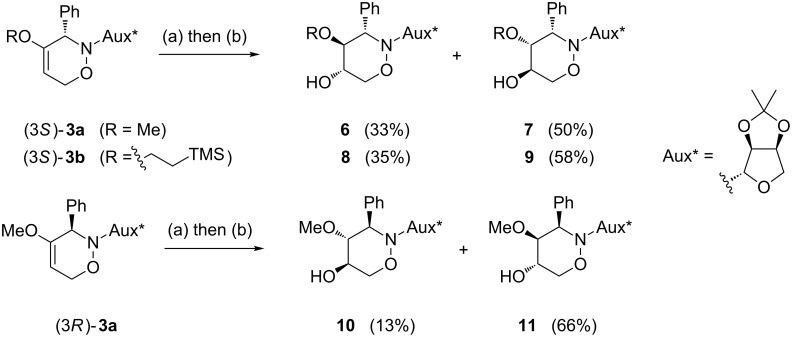
Introduction of a 5-hydroxy group into 1,2-oxazine derivatives **3** by a hydroboration/oxidation protocol; (a) BH_3_·THF (4.0 equiv), THF, −30 °C to rt, 3 h; (b) NaOH, H_2_O_2_ (30%), −10 °C to rt, overnight.

With respect to the enormous importance of polyhydroxylated *N*-heterocycles as carbohydrate-mimicking glycosidase inhibitors [41–45], the introduction of an additional hydroxyl moiety into 1,2-oxazine derivatives was an essential goal in several studies by our group [34,46–48]. A series of 5-hydroxy-1,2-oxazine derivatives was successfully prepared by the well known hydroboration/oxidation protocol, with yields and selectivities strongly depending on the relative configuration of the employed 1,2-oxazine derivative and on the presence of additives [34,48]. In general, *syn*-configured 1,2-oxazines (with respect to the relative configuration at C-3 and the neighbouring stereogenic centre at the carbohydrate-derived C-3-substituent) were found to be excellent substrates, leading to the desired alcohols exclusively with very high degrees of stereoselectivity. In an extension of these studies, selected compounds of type **3** were hydroxylated following the general methodology. As shown in Scheme 4, each of the (3*S*)-configured 1,2-oxazines **3a** and **3b** furnished a pair of hydroxylated products **6**, **7** and **8**, **9**, respectively, in high combined yields (83% and 93%) and almost the same ratio (approximately 3:2) of *cis-trans*/*trans-trans* isomers. The stereoselectivity for this series is apparently only low. On the other hand, for (3*R*)-**3a** a ca. 3:1 ratio of hydroxylated products was observed based on the ^1^H NMR spectrum of the crude mixture; however, the minor product **10** was isolated in 13% yield only, whereas the major product was obtained in a satisfying 66% yield. We assume that the observed low facial selectivity results predominantly from the moderate hindrance exhibited by the neighbouring phenyl substituent, which in the case of the (3*S*)-series probably occupies a pseudo-equatorial position in the half-chair conformation of the 3,6-dihydro-2*H*-1,2-oxazine derivatives **3**. The higher stereoselectivity of the hydroboration of (3*R*)-**3a** is probably caused by the pseudo-axial location of the phenyl group (as evidenced by the X-ray analysis, Figure 2), shielding one side more efficiently. The carbohydrate-derived *N*-substituent is relatively far away from the two reacting carbon atoms and very likely has no strong, direct influence on the observed diastereoselectivities.

All products **6**–**11** obtained by the hydroboration/oxidation protocol were easily purified and separated by column chromatography and finally deprotected by treatment with acid. This afforded a series of the desired, highly functionalised tetrahydro-2*H*-1,2-oxazine derivatives **12**–**15**, including *ent*-**12** and *ent*-**13** (Table 2). Reaction of the *trans-trans-*configured 4-methoxy-1,2-oxazines **6** and **10** with a methanolic solution of HCl (1 M) at elevated temperatures enabled the smooth cleavage of the glycosyl bond to give the *N*-unsubstituted derivatives **12** and *ent*-**12** in high yields (Table 2, entries 1 and 5). The *cis-trans-*configured compound pair **7** and **11** provided similar results, yielding the expected enantiomers **13** and *ent*-**13** (Table 2, entries 2 and 6). As expected, the enantiomers show nicely matching optical rotations with opposite sign. In the case of the TMSE-protected derivative **9**, selective removal of the *N*-protective group could be achieved under the applied conditions. After prolonged reaction times (16 h) there was no significant change in the tested sample. A complete deprotection of **9** leading to dihydroxylated compound **15** was possible in high overall yield (75%) by using ion-exchange resin DOWEX-50 at 50 °C (Table 2, entry 4). As shown for compound **8**, simultaneous cleavage was also possible, and the analytically pure compound **14** was isolated in comparable yield (Table 2, entry 3). Alternatively, demethylation of **12** by treatment with boron tribromide at low temperatures [49] also provided the expected compound **14** (Table 2, entry 7); however, the analytically pure sample of this compound could only be isolated in 18% yield. Therefore, the protocol applying a TMS-ethyl substituent as a more easily removable O-protective group turned out to be much more effective. All enantiopure 1,2-oxazines **12**–**15** were isolated as colourless crystals, which were prone to sublimation.

**Table 2:** Acid-induced cleavages of *N*- and *O*-protective groups of 5-hydroxy-1,2-oxazine derivatives **6**–**11**; conditions: (a) HCl (1 M) in MeOH, 40 °C, 3.5 h; (b) DOWEX-50, EtOH, 50 °C, 4 d; (c) BBr_3_ (3 equiv), CH_2_Cl_2_, −78 °C (1 h) then rt, overnight.

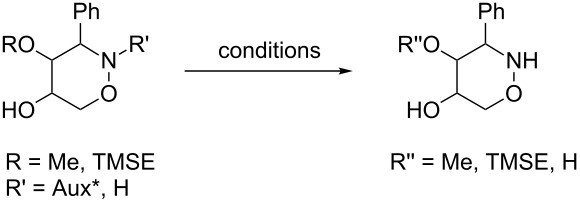					

entry	*N*-glycosyl 1,2-oxazine	conditions	product	yield	mp/[α]_D_^22^

1	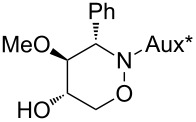 **6**	(a)	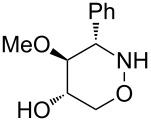 **12**	79%	110–112 °C/ +60.1 (*c* 1.05, CHCl_3_)
2	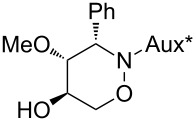 **7**	(a)	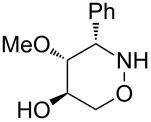 **13**	83%	112–113 °C/ +47.9 (*c* 1.00, CHCl_3_)
3	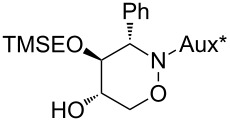 **8**	(b)^a^	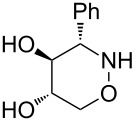 **14**	78%	153–154 °C/ +33.8 (*c* 1.05, CH_3_OH)
4	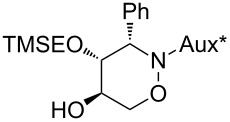 **9**	(a) then (b)	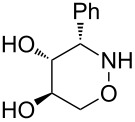 **15**	75%^b^	190–192 °C/ +65.6 (*c* 1.26, CH_3_OH)
5	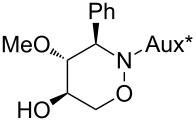 **10**	(a)	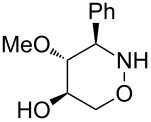 *ent*-**12**	84%	110–112 °C/ −61.5 (*c* 1.18, CHCl_3_)
6	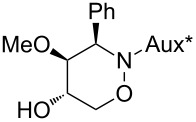 **11**	(a)	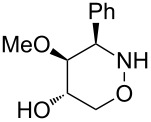 *ent*-**13**	92%	110–113 °C/ −48.8 (*c* 1.10, CHCl_3_)
7	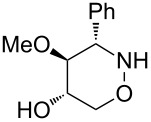 **12**	(c)	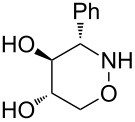 **14**	18%	—^c^

^a^Reaction time prolonged to 10 days; ^b^overall yield for two steps; ^c^melting point and spectroscopic data correspond with the sample of compound **14** obtained from **8** (Table 2, entry 3).

The ^1^H NMR spectrum of *trans*–*trans* configured **14** also deserves a short comment. Similarly to the previously described 2,4- and 2,5-dimethyltetrahydro-1,2-oxazine derivatives [50], significant long-range couplings could be observed in the ^1^H NMR spectrum. The low-field shifted multiplet (4.12–4.19 ppm) assigned to the equatorial 6-H showed additional couplings of <2.5 Hz. However, due to 4-H/5-H overlapping, selective decoupling of this complex spin system was not possible. An indirect proof for the observed phenomenon was found in the ^1^H NMR spectrum (Supporting Information File 1) of **14** prior to purification, i.e., still containing BBr_3_, which acts here as a shift reagent. The influence of the coordinated boron species resulted not only in a strong low-field shift but it also simplified the spectrum, and thus, only geminal and vicinal couplings (*J* = 12.2 Hz and *J* = 5.4 Hz) for the equatorial 6-H could be found. On the other hand, a possible nitrogen and/or ring inversion usually measurable at lower temperatures should be taken into account [51]. As expected, no significant changes in the shift pattern were observed in a series of ^1^H NMR spectra measured at elevated temperatures, both in methanol-*d*_4_ (up to 50 °C) and DMSO-*d*_6_ (up to 80 °C). Moreover, in the ^13^C NMR spectrum only one set of sharp signals was observed.

**Scheme 5:**
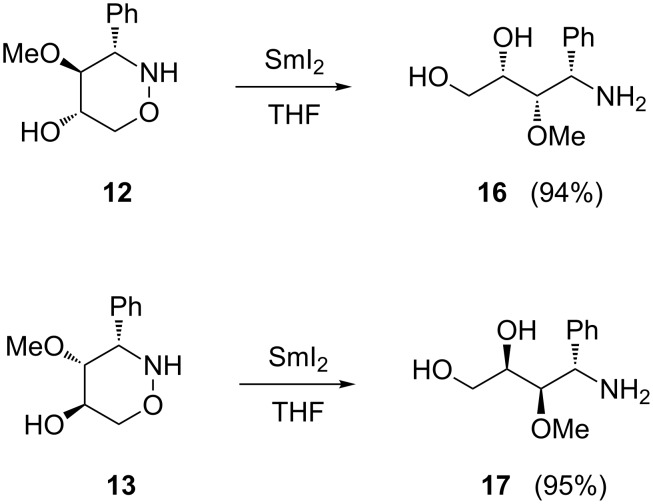
Samarium diiodide-induced ring opening of tetrahydro-2*H*-1,2-oxazine derivatives **12** and **13**.

Due to their similarity to carbohydrate derivatives, hydroxylated 1,2-oxazines such as **12**–**15** may already have interesting biological activity, but their functional groups also open several options for subsequent transformations into other relevant compound classes. By reductive ring opening the corresponding amino polyols should be accessible. Since compounds of type **12** contain a benzylamine substructure, standard methods that may possibly attack this moiety, such as catalytic hydrogenation, should be avoided. As an alternative, samarium diiodide is an attractive reagent for this purpose. Apart from its extraordinary potential for the formation of new carbon–carbon bonds [52–54], the cleavage of N–O bonds in a chemoselective fashion is also well documented [55–57]. The application of samarium diiodide for 1,2-oxazine ring opening allowed efficient syntheses of numerous polyhydroxylated heterocycles, such as pyrrolidine [46], azetidine [47], furan [58], and pyran derivatives [59]. Gratifyingly, the treatment of tetrahydro-2*H*-1,2-oxazine derivatives **12** and **13** with an excess of SmI_2_ in tetrahydrofuran smoothly provided the expected amino alcohols **16** and **17** in excellent yields (Scheme 5).

**Scheme 6:**
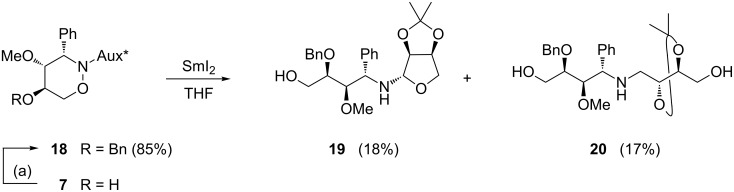
Reaction of tetrahydro-2*H*-1,2-oxazine **18** with samarium diiodide. (a) NaH (1.4 equiv), BnBr (1.2 equiv), DMF, 0 °C to rt, overnight.

In order to compare the behaviour of a compound still bearing the *N*-auxiliary, we converted tetrahydro-2*H*-1,2-oxazine **7** into the *O*-benzylated derivative **18** under standard conditions (Scheme 6). Treatment of this protected compound with samarium diiodide furnished a complex mixture of products from which only the two amino alcohols **19** and **20** were isolated, in low yield. The formation of **20** could be explained by a subsequent SmI_2_-mediated reduction of the C=N bond formed by ring opening of **19**, which contains a hemiaminal moiety. This suggestion is supported by the ^1^H NMR spectrum of **19** in which a second set of signals could be easily detected. Thus, the direct use of 1,2-oxazine derivatives still containing the carbohydrate-derived auxiliary at the nitrogen is apparently not sufficiently selective during samarium diiodide-promoted reactions.

**Scheme 7:**
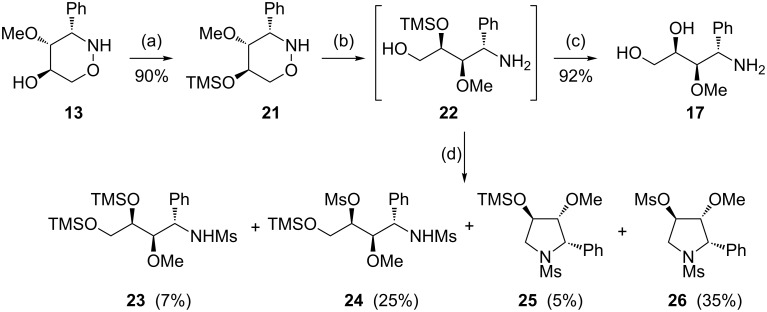
Attempted synthesis of pyrrolidine derivatives from precursor **13**. (a) TMSCl (1.5 equiv), imidazole, DMAP, CH_2_Cl_2_, rt, overnight; (b) SmI_2_, THF, 1.5 h, rt; (c) CHCl_3_, rt, overnight; (d) MsCl (4 equiv), Et_3_N, CH_2_Cl_2_, rt, overnight.

The successful transformation of *N*-benzyl-substituted tetrahydro-2*H*-1,2-oxazine derivatives into polyhydroxylated pyrrolidine derivatives [46] prompted us to select compound **13** as a precursor and to examine the described methods with this substrate. First, the free hydroxy group was protected as a trimethylsilyl ether and, after SmI_2_-induced ring opening, the expected product **22** was clearly identified based on TLC monitoring. However, the attempted isolation and purification of this compound by column chromatography provided amino alcohol **17** as the only product in high yield (92%). The limited stability of the TMS protective group is evident from the results presented in Scheme 7. Treatment of freshly prepared unpurified **22** with an excess of mesyl chloride and triethylamine yielded a complex product mixture. The isolated compounds **23**–**26** clearly indicate that the migration of the TMS group not only takes place in an intramolecular fashion to the terminal hydroxy function to furnish **24**, but it also occurs intermolecularly leading to the disilylated mesylamide **23**. The desired pyrrolidine derivative **25** was obtained only as a minor product (5%). The major isolated component, *N*,*O*-dimesylated pyrrolidine **26** (35%) derives from **25** by TMS-cleavage and subsequent mesylation of the OH group.

**Scheme 8:**
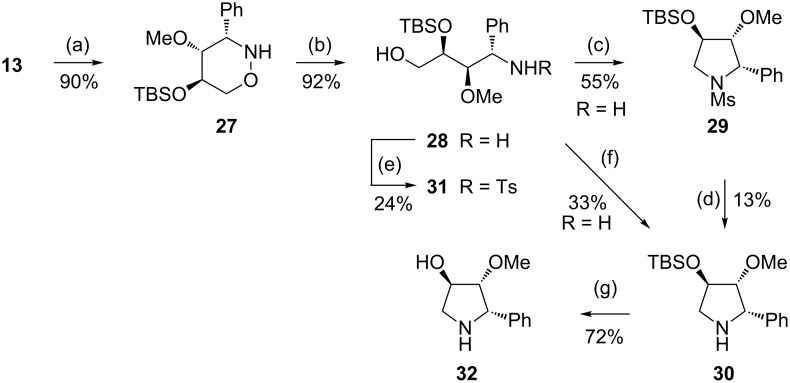
Synthesis of TBS-protected tetrahydro-2*H*-1,2-oxazine **27** and its transformation into pyrrolidine derivatives **29**, **30** and **32**. (a) TBSCl (2.0 equiv), imidazole, DMAP, CH_2_Cl_2_, rt, 5 d; (b) SmI_2_, THF, 1.5 h, rt; (c) MsCl (2.0 equiv), Et_3_N, CH_2_Cl_2_, rt, overnight; (d) LDA (5.4 equiv), rt, 16 h; (e) *p*TsCl (2.2 equiv), Et_3_N, CH_2_Cl_2_, rt, overnight; (f) CBr_4_ (1.2 equiv), PPh_3_ (1.2 equiv), Et_3_N (1.1 equiv), CH_2_Cl_2_, rt, overnight; (g) HCl (1 M) in MeOH, rt, 3 d.

To overcome these apparent difficulties, *tert*-butyldimethylsilyl-protected compound **27** was prepared. Samarium diiodide-mediated ring opening under standard conditions furnished the expected amino alcohol **28** in excellent yield (Scheme 8). An attempted cyclisation of **28** using tosyl chloride in the presence of triethylamine was not successful but led to *N*-tosylated compound **31** in 24% yield. A partial epimerisation at the benzylic position and slow decomposition of precursor **28** could also be observed under the reaction conditions applied, and none of the desired pyrrolidine derivatives could be found in the crude product. Purification on a silica gel column yielded two fractions containing a mixture of the C-4 epimeric *N*,*O*-di-tosylated compounds (14%, ca. 1:1 ratio) and a mixture of the respective tosylamides (41%, ca. 4:1 ratio). Additional chromatography of the latter fraction enabled isolation of compound **31** in the pure state (24%). Isolation of other by-products was not possible.

Fortunately, the use of mesyl chloride was more efficient to achieve cyclisation of **28**. Application of this reagent afforded pyrrolidine derivative **29** in acceptable overall yield. The different reaction outcome observed for the transformations of **28** with the two sulfonyl chlorides is probably a consequence of the bulkiness of the TBS group. The small sulfene intermediate, generated from mesyl chloride, smoothly reacts with the terminal OH group to give the respective mesylate, which subsequently cyclises to afford pyrrolidine **29**. On the other hand, the more bulky tosyl chloride competitively attacks the amino group. As illustrated in Scheme 8, the attempted conversion of **29** into the free secondary amine **30** by treatment with LDA [60] was not very efficient. The target compound was accompanied by a mixture of dihydropyrrole derivatives, which were very likely formed by deprotonation at the benzylic position and subsequent elimination.

Finally, freshly prepared unpurified **28** was subjected to the conditions of an Appel reaction [61] providing, after 16 hours at room temperature, pyrrolidine derivative **30** in 33% yield. Again, the relatively low efficacy could be explained by the destructive role of the base required for the subsequent cyclisation step. Cleavage of the TBS-moiety under acidic conditions furnished the desired hydroxylated pyrrolidine **32** in good yield. An attempted direct conversion of the unprotected amino diol **17** into **32** by treatment with tetrabromomethane in the presence of triphenylphosphine gave no satisfactory results, possibly due to the formation of the corresponding oxirane and its diverse, subsequent reactions.

## Conclusion

We achieved the efficient synthesis of enantiopure hydroxylated tetrahydro-2*H*-1,2-oxazine derivatives using, in the key step, lithiated alkoxyallenes and a phenyl-substituted nitrone **1a** bearing an L-erythronolactone-derived auxiliary as starting materials. Moderate levels of diastereoselectivity were observed for the formation of the 1,2-oxazine ring and for the subsequent hydroboration step. However, due to the easy separation of the formed products by standard column chromatography, the presented protocol opens up access to enantiopure products with both absolute configurations in different relative configurations, in a relatively short time. The described procedure supplements known protocols employing terpene units [62] and carbohydrate-derived auxiliaries [63–64] for the asymmetric synthesis of the 1,2-oxazine derivatives. More recently, the use of (–)-menthol as a chiral auxiliary was presented for the separation of diastereomeric 6*H*-1,2-oxazines [65–66]. Subsequent transformations of the newly prepared tetrahydro-2*H*-1,2-oxazines, utilizing samarium diiodide as the key reagent for the chemoselective ring opening, enable a smooth access to novel phenyl-substituted aminopolyols. Their transformation into hydroxylated pyrrolidine derivatives so far proceeds only with moderate efficacy, but this may certainly be optimised in the future.

## Experimental

**General methods.** Reactions were generally performed under an inert atmosphere (argon) in flame-dried flasks. Solvents and reagents were added by syringe. Solvents were purified with a MB SPS-800-dry solvent system. Triethylamine was distilled from CaH_2_ and stored over KOH under an atmosphere of argon. Other reagents were purchased and used as received without further purification unless stated otherwise. Products were purified by flash chromatography on silica gel (230–400 mesh, Merck or Fluka). Unless stated otherwise, yields refer to analytically pure samples. NMR spectra were recorded with Bruker (AC 250, AC 500, AVIII 700) and JOEL (ECX 400, Eclipse 500) instruments. Chemical shifts are reported relative to TMS or solvent residual peaks (^1^H: δ = 0.00 ppm [TMS], δ = 3.31 ppm [CD_3_OD], δ = 7.26 ppm [CDCl_3_]; ^13^C: δ = 49.0 ppm [CD_3_OD], δ = 77.0 ppm [CDCl_3_]). Integrals are in accordance with assignments and coupling constants are given in Hertz. All ^13^C NMR spectra are proton-decoupled. For detailed peak assignments, 2D spectra were measured (COSY, HMQC, HMBC). IR spectra were measured with a Nexus FT-IR spectrometer fitted with a Nicolet Smart DuraSample IR ATR. MS and HRMS analyses were performed with a Varian Ionspec QFT-7 (ESI–FT ICRMS) instrument. Elemental analyses were obtained with a Vario EL or a Vario EL III (Elementar Analysensysteme GmbH) instrument. Melting points were measured with a Reichert apparatus (Thermovar) and are uncorrected. Optical rotations ([α]_D_) were determined with a Perkin–Elmer 241 polarimeter at the temperatures given. Single crystal X-ray data were collected with a Bruker SMART CCD diffractometer (Mo Kα radiation, λ = 0.71073 Å, graphite monochromator); the structure solution and refinement was performed by using SHELXS-97 [67] and SHELXL-97 [67] in the WINGX system [68]. CCDC-864241 contains the supplementary crystallographic data for this paper. These data can be obtained free of charge from the Cambridge Crystallographic Data Centre via http://www.ccdc.cam.ac.uk/data_request/cif.

### Typical procedure for the preparation of 1,2-oxazine derivatives by addition of a lithiated alkoxyallene to nitrone **1a** (Procedure 1)

Lithiated methoxyallene was generated under an atmosphere of dry argon by treating a solution of methoxyallene (357 mg, 0.42 mL, 5.06 mmol) in dry THF (20 mL) with *n*-BuLi (2.5 M in hexanes; 2.0 mL, 5.0 mmol) at −40 °C. After 5 min, the resulting mixture was cooled to −130 °C (*n*-pentane/liq. N_2_ bath), and a solution of nitrone **1a** (606 mg, 2.30 mmol) in dry THF (15 mL) was added under vigorous stirring. The partially solidified mixture was allowed to reach −80 °C within 1.5 h and was quenched with water. Then, warming to room temperature was followed by extraction with Et_2_O (3 × 25 mL), and the combined organic layers were stirred overnight with the drying agent (MgSO_4_). When cyclisation of the primarily formed allene adducts was complete (TLC monitoring, hexane/ethyl acetate 4:1, *p*-anisaldehyde stain) the solvents were removed in vacuo to yield a light orange oil (763 mg). The crude material was filtered through a short silica gel pad (hexane/ethyl acetate 3:1) to yield a mixture of diastereomers (574 mg, 75%, 2:1 ratio), which were separated by column chromatography (silica gel, hexane/ethyl acetate 7:1, gradient to 5:1) to give (3*S*)-**3a** (380 mg, 49%, first eluted) as a pale yellow oil and (3*R*)-**3a** (170 mg, 22%) as a colourless solid. An analytically pure sample of (3*R*)-**3a** was obtained by recrystallisation from ethyl acetate.

**(3*S*,3a’*S*,4’*S*,6a’*S*)-2-(2’,2’-Dimethyltetrahydrofuro[3,4-*d*][1,3]dioxol-4’-yl)-4-methoxy-3-phenyl-3,6-dihydro-2*H*-[1,2]oxazine ((3*S*)-3a):** 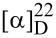 +133.4 (*c* 1.12, CHCl_3_); ^1^H NMR (CDCl_3_, 700 MHz) δ 1.32, 1.41 (2 s, 3H each, 2 Me), 3.47 (s, 3H, OMe), 4.03 (d, *J* = 9.5 Hz, 1H, 6’-H), 4.23–4.27 (dd_br_, *J* ≈ 3.5, 9.5 Hz, 1H, 6’-H), 4.30 (dd, *J* = 4.3, 13.7 Hz, 1H, 6-H), 4.40 (s, 1H, 4’-H), 4.62 (dt_br_, *J* ≈ 2.0, 13.7 Hz, 1H, 6-H), 4.85 (s_br_, 1H, 3-H), 4.87 (dt_br_, *J* ≈ 1.3, 4.3 Hz, 1H, 5-H), 4.88–4.90 (m, 2H, 3a’-H, 6a’-H), 7.26–7.33, 7.34–7.38 (2 m, 5H, Ph) ppm; ^13^C NMR (CDCl_3_, 126 MHz) δ 24.5, 26.3 (2 q, 2 Me), 54.8 (q, OMe), 63.5 (d, C-3), 67.3 (t, C-6), 76.6 (t, C-6’), 81.2, 84.2 (2 d, C-3a’, C-6a’), 92.1 (d, C-5), 94.7 (d, C-4’), 111.6 (s, C-2’), 128.0, 128.3, 129.7, 136.2 (3 d, s, Ph), 154.9 (s, C-4) ppm; IR (ATR) 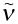: 3085–2840 (=C-H, C-H), 1670 (C=C), 1225, 1075, 1055 (C-O) cm^−1^; ESI–TOF (*m*/*z*): [M + Na]^+^ calcd for C_18_H_23_NNaO_5_, 356.1474; found, 356.1479; Anal. calcd for C_18_H_23_NO_5_ (333.4): C, 64.85; H, 6.95; N, 4.20; found: C, 64.81; H, 6.98; N, 4.15.

**(3*R*,3a’*S*,4’*S*,6a’*S*)-2-(2’,2’-Dimethyltetrahydrofuro[3,4-*d*][1,3]dioxol-4’-yl)-4-methoxy-3-phenyl-3,6-dihydro-2*H*-[1,2]oxazine ((3*R*)-3a):** mp 110–113 °C; crystals suitable for X-ray analysis were obtained from AcOEt solution by cooling (fridge); Crystal data: C_18_H_23_NO_5_, *M* = 333.37, orthorhombic, *a* = 5.6042(12) Å, *b* = 16.756(4) Å, *c* = 17.839(4) Å, α = 90.00°, β = 90.00°, γ = 90.00°, *V* = 1675.2(6) Å^3^, *T* = 133(2) K, space group *P*2(1)2(1)2(1), *Z* = 4, Mo Kα, 23651 reflections measured, 4186 independent reflections (*R*_int_ = 0.0178). *R*_1_ = 0.0307 (*I* > 2σ(*I*)); *wR*(*F*^2^) = 0.0782 (all data); GOOF(*F*^2^) = 1.048. 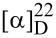 −87.0 (*c* 1.36, CHCl_3_); ^1^H NMR (CDCl_3_, 500 MHz) δ 1.34, 1.44 (2 s, 3H each, 2 Me), 3.49 (s, 3H, OMe), 3.89 (d, *J* = 9.9 Hz, 1H, 6’-H), 4.03 (dd, *J* = 4.0, 9.9 Hz, 1H, 6’-H), 4.41 (ddd, *J* = 1.7, 3.2, 14.3 Hz, 1H, 6-H), 4.48 (s_br_, 1H, 3-H), 4.54 (ddd, *J* = 1.6, 2.4, 14.3 Hz, 1H, 6-H), 4.72 (s, 1H, 4’-H), 4.80 (dd, *J* = 4.0, 6.1 Hz, 1H, 6a’-H), 4.90 (t_br_, *J* ≈ 3.0 Hz, 1H, 5-H), 5.03 (d, *J* = 6.1 Hz, 1H, 3a’-H), 7.27–7.34, 7.36–7.40 (2 m, 5H, Ph) ppm; ^13^C NMR (CDCl_3_, 126 MHz) δ 24.7, 26.3 (2 q, 2 Me), 54.6 (q, OMe), 63.6 (d, C-3), 65.2 (t, C-6), 74.5 (t, C-6’), 81.1 (d, C-6a’), 81.5 (d, C-3a’), 91.7 (d, C-5), 96.4 (d, C-4’), 112.0 (s, C-2’), 127.8, 128.3, 129.0, 138.1 (3 d, s, Ph), 153.1 (s, C-4) ppm; IR (ATR) 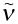: 3060–2840 (=C-H, C-H), 1675 (C=C), 1220, 1100, 1050 (C-O) cm^−1^; ESI–TOF (*m*/*z*): [M + Na]^+^ calcd for C_18_H_23_NNaO_5_, 356.1474; found, 356.1470; Anal. calcd for C_18_H_23_NO_5_ (333.4): C, 64.85; H, 6.95; N, 4.20; found: C, 64.85; H, 6.83; N, 4.11.

### Typical procedure for hydroborations of 1,2-oxazines (Procedure 2)

To a solution of 1,2-oxazine (3*S*)-**3a** (268 mg, 0.80 mmol) in dry THF (20 mL), a solution of BH_3_·THF (1 M in THF, 3.2 mL, 3.2 mmol) was added at −30 °C. The solution was warmed to room temperature and stirred for 3 h, then cooled to −10 °C and an aq NaOH solution (2 M, 4.8 mL) followed by H_2_O_2_ (30%, 1.6 mL) were added. Stirring at room temperature was continued overnight. After addition of a sat. aq Na_2_S_2_O_3_ solution, the layers were separated, the water layer was extracted with Et_2_O (3 × 15 mL), the combined organic layers were dried with MgSO_4_ and filtered, and the solvents were removed under reduced pressure. The crude products (321 mg, 3:2 ratio) were separated by chromatography column (silica gel, hexane/ethyl acetate 1:1) to give 5-hydroxy-1,2-oxazines **6** (92 mg, 33%, first eluted) and **7** (141 mg, 50%) as hygroscopic, colourless semisolids.

**(3*S*,4*S*,5*S*,3’a*S*,4’*S*,6’a*S*)-2-(2’,2’-Dimethyltetrahydrofuro[3,4-*d*][1,3]dioxol-4’-yl)-4-methoxy-3-phenyl-[1,2]oxazinan-5-ol (6):** 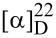 +131.2 (*c* 1.02, CHCl_3_); ^1^H NMR (CDCl_3_, 500 MHz) δ 1.28, 1.35 (2 s, 3H each, 2 Me), 2.60 (d, *J* = 1.9 Hz, 1H, OH), 2.90 (s, 3H, OMe), 3.41 (ddd, *J* = 1.4, 6.7, 9.4 Hz, 1H, 4-H), 3.66–3.72 (m, 2H, 5-H, 6-H), 3.93 (d, *J* = 9.4 Hz, 1H, 3-H), 3.93 (d, *J* = 9.5 Hz, 1H, 6’-H), 4.07 (dd, *J* ≈ 11, 16 Hz, 1H, 6-H), 4.19 (dd, *J* = 4.4, 9.5 Hz, 1H, 6’-H), 4.41 (s, 1H, 4’-H), 4.81 (dd, *J* = 4.4, 6.1 Hz, 1H, 6a’-H), 4.86 (d, *J* = 6.1 Hz, 1H, 3a’-H), 7.28–7.42 (m, 5H, Ph) ppm; ^13^C NMR (CDCl_3_, 126 MHz) δ 24.5, 26.2 (2 q, 2 Me), 60.5 (q, OMe), 67.6 (d, C-3), 70.6 (d, C-5), 71.4 (t, C-6), 77.4 (t, C-6’), 81.3 (d, C-6a’), 84.4 (d, C-3a’), 87.6 (d, C-4), 94.8 (d, C-4’), 111.7 (s, C-2’), 128.3, 128.8*, 136.8 (2 d, s, Ph) ppm; *higher intensity; IR (ATR) 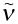: 3440 (O-H), 3090–2830 (=C-H, C-H), 1205, 1055 (C-O) cm^−1^; ESI–TOF (*m*/*z*): [M + Na]^+^ calcd for C_18_H_25_NNaO_6_, 374.1580; found, 374.1581; Anal. calcd for C_18_H_25_NO_6_ (351.4): C, 61.52; H, 7.17; N, 3.99; found: C, 61.43; H, 7.15; N, 3.85.

**(3*S*,4*R*,5*R*,3a’*S*,4’*S*,6a’*S*)-2-(2’,2’-Dimethyltetrahydrofuro[3,4-*d*][1,3]dioxol-4’-yl)-4-methoxy-3-phenyl-[1,2]oxazinan-5-ol (7):** 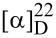 +138.2 (*c* 1.41, CHCl_3_); ^1^H NMR (CDCl_3_, 500 MHz) δ 1.28, 1.34 (2 s, 3H each, 2 Me), 2.44 (d, *J* = 7.7 Hz, 1H, OH), 3.10 (s, 3H, OMe), 3.21 (m_c_, 1H, 4-H), 3.75 (s_br_, 1H, 5-H), 3.82 (d, *J* = 12.2 Hz, 1H, 6-H), 3.94 (d, *J* = 9.4 Hz, 1H, 6’-H), 4.21 (dd, *J* = 4.6, 9.4 Hz, 1H, 6’-H), 4.36 (dd, *J* = 1.4, 12.2 Hz, 1H, 6-H), 4.45 (d, *J* = 2.3 Hz, 1H, 3-H), 4.60 (s, 1H, 4’-H), 4.81 (t_br_, *J* ≈ 5.2 Hz, 1H, 6a’-H), 4.94 (d, *J* = 6.1 Hz, 1H, 3a’-H), 7.24–7.31, 7.42–7.45 (2 m, 5H, Ph) ppm; ^13^C NMR (CDCl_3_, 126 MHz) δ 24.5, 26.3 (2 q, 2 Me), 59.3 (q, OMe), 62.7 (d, C-3), 65.3 (d, C-5), 71.0 (t, C-6), 77.5 (t, C-6’), 80.4 (d, C-4), 81.1 (d, C-6a’), 84.5 (d, C-3a’), 95.6 (d, C-4’), 111.6 (s, C-2’), 127.8, 128.3, 129.5, 136.4 (3 d, s, Ph) ppm; IR (ATR) 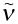: 3455 (O-H), 3090–2830 (=C-H, C-H), 1215, 1085, 1050 (C-O) cm^−1^; ESI–TOF (*m*/*z*): [M + Na]^+^ calcd for C_18_H_25_NNaO_6_, 374.1580; found, 374.1579; Anal. calcd for C_18_H_25_NO_6_ (351.4): C, 61.52; H, 7.17; N, 3.99; found: C, 61.43; H, 7.17; N, 3.87.

### Typical protocol for glycosyl bond cleavage (Procedure 3)

1,2-Oxazine **6** (425 mg, 1.21 mmol) was dissolved in 1 N HCl in MeOH (14 mL) and heated at 40 °C for 3.5 h (TLC monitoring, hexane/AcOEt 1:2, potassium permanganate stain). Then the mixture was allowed to reach room temperature, quenched with sat. aq NaHCO_3_ solution and extracted with Et_2_O (3 × 30 mL). The combined organic layers were dried with MgSO_4_ and filtered, and the solvents were removed. The purification by column chromatography (silica gel, dichloromethane/methanol 40:1) yielded **12** (201 mg, 79%) as a colourless solid.

**(3*S*,4*S*,5*S*)-4-Methoxy-3-phenyl-[1,2]oxazinan-5-ol (12):** mp 110–112 °C; 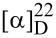 +60.1 (*c* 1.05, CHCl_3_); ^1^H NMR (CDCl_3_, 500 MHz) δ 3.02 (s, 3H, OMe), 3.37 (t_br_, *J* ≈ 8.5 Hz, 1H, 4-H), 3.71–3.81 (m, 2H, 5-H, 6-H), 3.91 (d, *J* = 9.2 Hz, 1H, 3-H), 4.14 (dd, *J* = 4.1, 9.7 Hz, 1H, 6-H), 2.60, 5.48 (2 s_br_, 2H, NH, OH), 7.31–7.38, 7.40–7.43 (2 m, 5H, Ph) ppm; ^13^C NMR (CDCl_3_, 126 MHz) δ 60.4 (q, OMe), 67.0 (t, C-3), 70.9 (d, C-5), 72.3 (t, C-6), 86.9 (d, C-4), 128.4, 128.6, 128.7, 136.3 (3 d, s, Ph) ppm; IR (ATR) 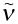: 3405–3260 (O-H, N-H), 3065–2830 (=C-H, C-H), 1105, 1055 (C-O) cm^−1^; ESI–TOF (*m*/*z*): [M + H]^+^ calcd for C_11_H_16_NO_3_, 210.1130; found, 210.1127; Anal. calcd for C_11_H_15_NO_3_ (209.2): C, 63.14; H, 7.23; N, 6.69; found: C, 63.14; H, 7.23; N, 6.66.

### Typical procedure for the reactions with samarium diiodide (Procedure 4)

To a solution of SmI_2_ (ca. 0.1 M in THF, 15 mL, ~1.5 mmol) at room temperature was added dropwise a solution of 5-hydroxy-1,2-oxazine **12** (102 mg, 0.49 mmol) in degassed THF (10 mL). After the mixture was stirred for 3 h it was quenched with sat. aq sodium potassium tartrate solution and extracted with Et_2_O (20 mL), and then with CH_2_Cl_2_ (3 × 15 mL). The combined organic layers were dried with MgSO_4_, filtered and the solvents were removed under reduced pressure to give the spectroscopically pure product as a yellow oil in almost quantitative yield. Filtration through a short silica gel pad (dichloromethane/methanol 15:1) yielded **16** (97 mg, 94%) as a colourless oil.

**(2*S*,3*S*,4*S*)-4-Amino-3-methoxy-4-phenylbutane-1,2-diol (16):** 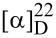 +12.2 (*c* 1.48, CHCl_3_); ^1^H NMR (CDCl_3_, 500 MHz) δ 3.31 (dd, *J* = 2.1, 5.3 Hz, 1H, 3-H), 3.39 (s, 3H, OMe), 3.57 (dd, *J* = 4.6, 11.1 Hz, 1H, 1-H), 3.72 (dd, *J* = 6.1, 11.1 Hz, 1H, 1-H), 3.77–3.80 (m, 1H, 2-H), 4.58 (d, *J* = 5.3 Hz, 1H, 4-H), 7.28–7.33, 7.36–7.43 (2 m, 5H, Ph) ppm; ^13^C NMR (CDCl_3_, 126 MHz) δ 55.9 (d, C-4), 59.3 (q, OMe), 63.4 (t, C-1), 70.7 (d, C-2), 83.0 (d, C-3), 127.1, 128.1, 128.8, 138.6 (3 d, s, Ph) ppm; IR (ATR) 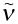: 3490–3230 (O-H, N-H), 3065–2810 (=C-H, C-H), 1075 (C-O) cm^−1^; ESI–TOF (*m*/*z*): [M + H]^+^ calcd for C_11_H_18_NO_3_, 212.1292; found, 212.1282.

## Supporting Information

File 1 Experimental procedures and characterisation data.

File 2 ^1^H NMR and ^13^C NMR spectra of synthesised compounds.
