# Supplementary material for: Carbohydrate-auxiliary assisted preparation of enantiopure 1,2-oxazine derivatives and aminopolyols
Source: Beilstein J Org Chem. 2012 Apr 30;8:662–74. doi: 10.3762/bjoc.8.74 (PMC3388852; doi:10.3762/bjoc.8.74)
Supplement: File 1 — Experimental procedures and characterisation data. [file Beilstein_J_Org_Chem-08-662-s001.pdf]

Supporting Information File 1

for

Carbohydrate-auxiliary assisted preparation of enantiopure 1,2-oxazine derivatives and aminopolyols

Marcin Jasiński[1,2], Dieter Lentz[1,§] and Hans-Ulrich Reissig*[,1]

Address: [1]Institut für Chemie und Biochemie, Freie Universität Berlin, Takustr. 3, D-14195 Berlin, Germany and [2]Department of Organic and Applied Chemistry, University of Łódź, Tamka 12, PL-91-403 Poland

Email: Hans-Ulrich Reissig* - hreissig@chemie.fu-berlin.de

* Corresponding author

[§] Responsible for X-ray crystal structure determination

Experimental procedures and characterisation data

Methoxyallene [1], TMSE-allene [2], and benzyloxyallene [2] were prepared following literature procedures.

Preparation of 1,2-oxazines (3*S*)-3b and (3*R*)-3b

By a procedure similar to that for methoxyallene (Procedure 1), TMSE-allene (411 mg, 2.63 mmol) in THF (40 mL) was treated with *n*-BuLi (2.5 M in hexanes;

1.0 mL, 2.5 mmol) at –40 °C, followed by slow addition of nitrone **1a** (263 mg, 1.00 mmol) in dry THF (6 mL) at –130 °C. The mixture was allowed to reach –80 °C within 2.5 h. After standard workup, crude products were isolated, by filtration through silica gel pad (hexane/ethyl acetate 6:1), as a yellow oil. Diastereomers were separated by an additional column (silica gel, hexane/ethyl acetate 9:1) to give (3*S*)-**3b** (133 mg, 32%, >95% purity) and (3*R*)-**3b** (78 mg, 19%) as colourless oils.

(3*S*,3a'*S*,4'*S*,6a'*S*)-2-(2',2'-Dimethyltetrahydrofuro[3,4-*d*][1,3]dioxol-4'-yl)-4-(2-trimethylsilylethoxy)-3-phenyl-3,6-dihydro-2*H*-[1,2]oxazine ((3*S*)-3b):

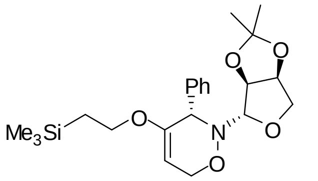

$[\alpha]_D^{22}$ = +107.2 (*c* 1.03, $CHCl_3$); ^{1}H NMR ($CDCl_3$, 700 MHz): δ = –0.15 (s, 9H, $SiMe_3$), 0.79 (ddd, *J* = 5.8, 8.7, 14.4 Hz, 1H, CH_2Si), 0.85 (ddd, *J* = 7.2, 9.0, 14.4 Hz, 1H, CH_2Si), 1.31, 1.41 (2 s, 3H each, 2 Me), 3.68 (td, *J* ≈ 7.2, 9.2 Hz, 1H, 4-OCH_2), 3.79 (dt, *J* ≈ 5.8, 9.2 Hz, 1H, 4-OCH_2), 4.02 (d, *J* = 9.6 Hz, 1H, 6'-H), 4.24 (dd, *J* = 3.5, 9.6 Hz, 1H, 6'-H), 4.28 (dd_{br}, *J* ≈ 3.7, 13.7 Hz, 1H, 6-H), 4.39 (s, 1 H, 4'-H), 4.60 (dt, *J* ≈ 2.0, 13.7 Hz, 1H, 6-H), 4.80 (s_{br}, 1 H, 3-H), 4.82 (dt, *J* ≈ 1.6, 4.2 Hz, 1H, 5-H), 4.86–4.89 (m, 2H, 3a'-H, 6a'-H), 7.26–7.31, 7.34–7.37 (2 m, 5H, Ph) ppm; ^{13}C NMR ($CDCl_3$, 126 MHz): δ = –1.6 (q, $SiMe_3$), 16.8 (t, CH_2Si), 24.6, 26.3 (2 q, 2 Me), 63.7 (d, C-3), 64.8 (t, 4-OCH_2-), 67.4 (t, C-6), 76.6 (t, C-6'), 81.2, 84.3 (2 d, C-3a', C-6a'), 92.2 (d, C-5), 94.8 (d, C-4'), 111.6 (s, C-2'), 127.8, 128.2, 129.9, 136.5 (3 d, s, Ph), 154.3 (s, C-4) ppm; IR (ATR): $\tilde{\nu}$ = 3065–2850 (=C-H, C-H), 1730 (C=C), 1210, 1060 (C-O) cm^{-1}; ESI-TOF (*m*/*z*): calcd. for $C_{22}H_{33}NNaO_5Si$ $[M + Na]^+$: 442.2025; found: 442.2014.

(3*R*,3a'*S*,4'*S*,6a'*S*)-2-(2',2'-Dimethyltetrahydrofuro[3,4-*d*][1,3]dioxol-4'-yl)-4-(2-trimethylsilylethoxy)-3-phenyl-3,6-dihydro-2*H*-[1,2]oxazine ((3*R*)-3b):

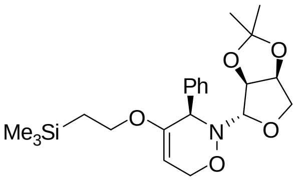

$[\alpha]_D^{22}$ = –55.7 (*c* 1.25, $CHCl_3$); ^{1}H NMR ($CDCl_3$, 500 MHz): δ = –0.13 (s, 9H, $SiMe_3$), 0.79 (ddd, *J* = 5.8, 8.9, 14.3 Hz, 1H, CH_2Si), 0.85 (ddd, *J* = 7.1, 9.2, 14.3 Hz, 1H, CH_2Si), 1.33, 1.44 (2 s, 3H each, 2 Me), 3.68 (td, *J* ≈ 7.1, 9.2 Hz, 1H, 4-OCH_2), 3.81 (td, *J* ≈ 5.8, 9.2 Hz, 1H, 4-OCH_2), 3.89 (d, *J* = 9.9 Hz, 1H, 6'-H), 4.03 (dd, *J* = 4.0, 9.9 Hz, 1H, 6'-H), 4.39 (ddd, *J* = 1.7, 3.1, 14.2 Hz, 1H, 6-H), 4.44 (s, 1H, 3-H), 4.51 (ddd, *J* = 1.3, 2.4, 14.2 Hz, 1H, 6-H), 4.73 (s, 1H, 4'-H), 4.80 (dd, *J* = 4.0, 6.1 Hz, 1H, 6a'-H), 4.84 (t, *J* ≈ 3.0 Hz, 1H, 5-H), 5.03 (d, *J* = 6.1 Hz, 1H, 3a'-H), 7.26–7.31, 7.36-7.39 (2 m, 5H, Ph) ppm; ^{13}C NMR ($CDCl_3$, 126 MHz): δ = –1.5 (q, $SiMe_3$), 16.9 (t, CH_2Si), 24.8, 26.3 (2 q, 2 Me), 63.7 (d, C-3), 64.6 (t, 4-OCH_2), 65.2 (t, C-6), 74.5 (t, C-6'), 81.1 (d, C-6a'), 81.6 (d, C-3a'), 91.8 (d, C-5), 96.4 (d, C-4'), 112.0 (s, C-2'), 127.6, 128.1, 129.1, 138.3 (3 d, s, Ph), 152.2 (s, C-4) ppm; IR (ATR): $\tilde{\nu}$ = 3060–2845 (=C-H, C-H), 1730 (C=C), 1105, 1055 (C-O) cm^{-1}; ESI-TOF (*m*/*z*): calcd. for $C_{22}H_{33}NNaO_5Si$ [M + Na]$^+$: 442.2025; found: 442.2010; Anal. calcd. for $C_{22}H_{33}NO_5Si$ (419.6): C 62.98, H 7.97, N 3.34; found: C 62.99, H 7.99, N 3.35.

Preparation of 1,2-oxazines (3*S*)-3c and (3*R*)-3c

By the procedure similar to that for methoxyallene (Procedure 1), benzyloxyallene (719 mg, 4.92 mmol) in THF (45 mL) was treated with *n*-BuLi (2.5 M in hexanes; 1.8 mL, 4.5 mmol) at –40 °C, followed by slow addition of nitrone **1a** (540 mg, 2.05 mmol) in dry THF (10 mL) at –130 °C. The mixture was allowed to reach –80 °C

within 1.5 h. After standard workup, crude products were separated on a column (silica gel, hexane/ethyl acetate 6:1) to give enriched fractions of (3*S*)-**3c** (235 mg) and (3*R*)-**3c** (394 mg) as pale yellow oils. Each fraction was additionally chromatographed (silica gel, hexane/acetone 6:1, and 5:1, resp.) to give pure (3*S*)-**3c** (182 mg, 21%) and (3*R*)-**3c** (371 mg, 44%) as colourless oils.

(3*S*,3a'*S*,4'*S*,6a'*S*)-4-Benzyloxy-2-(2',2'-dimethyltetrahydrofuro[3,4-*d*][1,3]dioxol-4'-yl)-3-phenyl-3,6-dihydro-2*H*-[1,2]oxazine ((3*S*)-3c):

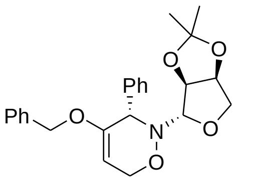

$[\alpha]_D^{22}$ = +109.8 (*c* 1.40, $CHCl_3$); ^{1}H NMR ($CDCl_3$, 700 MHz): δ = 1.31, 1.41 (2 s, 3H each, 2 Me), 4.03 (d, *J* = 9.6 Hz, 1H, 6'-H), 4.23-4.27 (m, 2H, 6'-H, 6-H), 4.44 (s, 1H, 4'-H), 4.59 (dt, *J* ≈ 2.0, 13.8 Hz, 1H, 6-H), 4.70, 4.77 (2 d, *J* = 12.3 Hz, 2H, Bn), 4.87–4.89 (m, 2H, 3a'-H, 6a'-H), 4.90 (dt, *J* ≈ 1.6, 4.3 Hz, 1H, 5-H), 4.91 (s_{br}, 1H, 3-H), 7.00–7.02, 7.20–7.25, 7.29–7.34, 7.40–7.42 (4 m, 10H, 2 Ph) ppm; ^{13}C NMR ($CDCl_3$, 175 MHz): δ = 24.6, 26.3 (2 q, 2 Me), 63.6 (d, C-3), 67.2 (t, C-6), 69.0 (t, Bn), 76.6 (t, C-6'), 81.2, 84.3 (2 d, C-3a', C-6a'), 93.6 (d, C-5), 94.8 (d, C-4'), 111.7 (s, C-2'), 126.5, 127.4, 128.0, 128.2*, 129.8, 136.4, 136.9 (5 d, 2 s, 2 Ph), 153.7 (s, C-4) ppm, *higher intensity; IR (ATR): $\tilde{\nu}$ = 3090–2850 (=C-H, C-H), 1675 (C=C), 1202, 1090, 1070, 1055 (C-O) cm^{-1}; ESI-TOF (*m*/*z*): calcd. for $C_{24}H_{27}NNaO_5$ [M + Na]$^+$: 432.1787; found: 432.1792. Anal. calcd. for $C_{24}H_{27}NO_5$ (409.5): C 70.40, H 6.65, N 3.42; found: C 68.70, H 6.58, N 3.44.

(3*R*,3a'*S*,4'*S*,6a'*S*)-4-Benzyloxy-2-(2',2'-dimethyltetrahydrofuro[3,4-*d*][1,3]dioxol-4'-yl)-3-phenyl-3,6-dihydro-2*H*-[1,2]oxazine ((3*R*)-3c):

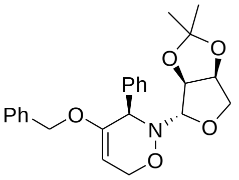

$[\alpha]_D^{22}$ = –51.3 (*c* 1.57, $CHCl_3$); ^1H NMR ($CDCl_3$, 500 MHz): δ = 1.35, 1.46 (2 s, 3H each, 2 Me), 3.91 (d, *J* = 9.9 Hz, 1H, 6'-H), 4.05 (dd, *J* = 4.0, 9.9 Hz, 1H, 6'-H), 4.40 (ddd, *J* = 1.7, 3.3, 14.3 Hz, 1H, 6-H), 4.53 (ddd, *J* = 1.7, 2.4, 14.3 Hz, 1H, 6-H), 4.57 (s, 1H, 3-H), 4.72 (d, *J* = 12.1 Hz, 1H, Bn), 4.76 (s, 1H, 4'-H), 4.80 (d, *J* = 12.1 Hz, 1H, Bn), 4.82 (dd, *J* = 4.0, 6.1 Hz, 1H, 6a'-H), 4.96 (t, *J* ≈ 3.0 Hz, 1H, 5-H), 5.05 (d, *J* = 6.1 Hz, 1H, 3a'-H), 7.06–7.08, 7.22–7.35, 7.41–7.44 (3 m, 10H, 2 Ph) ppm; ^{13}C NMR ($CDCl_3$, 126 MHz): δ = 24.8, 26.3 (2 q, 2 Me), 63.6 (d, C-3), 65.1 (t, C-6), 69.0 (t, Bn), 74.5 (t, C-6'), 81.0 (d, C-6a'), 81.6 (d, C-3a'), 93.2 (d, C-5), 96.5 (d, C-4'), 112.0 (s, C-2'), 126.7, 127.6, 127.7, 128.2, 128.3, 129.0 136.7 138.2 (6 d, 2 s, 2 Ph), 152.0 (s, C-4) ppm; IR (ATR): $\tilde{\nu}$ = 3090–2845 (=C-H, C-H), 1675 (C=C), 1220, 1205, 1090 (C-O) cm^{-1}; ESI-TOF (*m*/*z*): calcd. for $C_{24}H_{27}NNaO_5$ $[M + Na]^+$: 432.1787; found: 432.1766. Anal. calcd. for $C_{24}H_{27}NO_5$ (409.5): C 70.40, H 6.65, N 3.42; found: C 70.41, H 6.63, N 3.41.

Isolation of by-products 4 and 5

Compounds **4** (8 mg, 1%) and **5** (38 mg, 3%) were isolated as colourless oils from the reaction of lithiated methoxyallene (2.4 equiv.) generated from methoxyallene (640 mg, 0.77 mL, 9.13 mmol) and *n*-BuLi (2.5 M in hexanes; 3.5 mL, 8.75 mmol) with nitrone **1a** (960 mg, 3.65 mmol) at –78 °C, after standard workup (see Procedure 1) and additional purification on column. Diene **4** undergoes fast

decomposition either during purification on silica or storage. Major products (3*S*)-**3a** (303 mg) and (3*R*)-**3a** (92 mg) were isolated in 25% and 8% yield, respectively.

(*Z*)-2-Methoxy-1-phenylbuta-1,3-diene (4) [3,4]:

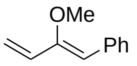

CC (silica gel, hexane/dichloromethane 7:3). ^{1}H NMR ($CDCl_3$, 500 MHz): δ = 3.77 (s, 3 H, OMe), 5.22 (dt, *J* ≈ 2.1, 11.0 Hz, 1 H, 4-H), 5.75 (dd, *J* = 2.1, 17.1 Hz, 1 H, 4-H), 5.84 (s, 1 H, 1-H), 6.63 (dd, *J* = 11.0, 17.1 Hz, 1 H, 3-H), 7.17–7.24, 7.29-7.34 (2 m, 5 H, Ph) ppm; ^{13}C NMR ($CDCl_3$, 126 MHz): δ = 54.8 (q, OMe), 103.9 (d, C-1), 116.0 (t, C-4), 125.8, 128.1, 129.4, 136.6 (3 d, s, Ph), 129.6 (d, C-3), 154.0 (s, C-2) ppm.

(4’*S*,5’*S*)-2-(2’,2’-Dimethyl-5’-hydroxymethyl-[1,3]dioxolan-4’-yl)-4-methoxy-3-methyl-5-phenyl-1*H*-pyrrole (5):

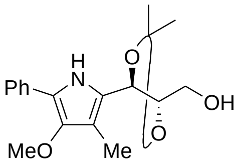

CC (silica gel, hexane/ethyl acetate 4:1). $[\alpha]_D^{22}$ = –43.1 (*c* 0.80, $CHCl_3$); ^{1}H NMR ($CDCl_3$, 500 MHz): δ = 1.49, 1.62 (2 s, 3H each, 2 Me), 2.08 (s, 3H, 3-Me), 2.11 (dd, *J* ≈ 4.0, 8.1 Hz, 1H, OH), 3.45 (ddd, *J* = 3.8, 8.1, 12.1 Hz, 1H, 5’-CH_2-), 3.61 (dt, *J* = 4.0, 12.1 Hz, 1H, 5’-CH_2-), 3.72 (s, 3H, OMe), 4.33 (dt, *J* = 3.8, 7.3 Hz, 1H, 5’-H), 5.37 (d, *J* = 7.3 Hz, 1H, 4’-H), 7.15-7.19, 7.34–7.38, 7.60-7.64 (3 m, 5H, Ph), 8.98 (s_{br}, 1 H, NH) ppm; ^{13}C NMR ($CDCl_3$, 126 MHz): δ = 7.6 (q, 3-Me), 24.3, 27.1 (2 q, 2 Me), 61.3 (q, OMe), 62.2 (t, 5’-CH_2-), 71.7 (d, C-4’), 77.5 (d, C-5’), 107.8 (s, C-2’), 112.4, 119.3, 119.4 (3 s, C-2, C-3, C-5), 123.9, 125.5, 128.7, 131.9 (3 d, s, Ph), 143.9 (s, C-4) ppm; IR (ATR): $\tilde{\nu}$ = 3470-3340 (O-H, N-H), 3080–2830 (=C-H, C-H),

1210, 1035 (C-O) cm^{-1}; ESI-TOF (*m*/*z*): calcd. for $C_{18}H_{23}NNaO_4$ [M + Na]$^+$: 340.1525; found: 340.1566; Anal. calcd. for $C_{18}H_{23}NO_4$ (317.4): C 68.17, H 7.30, N 4.41; found: C 68.19, H 7.39, N 4.37.

Hydroxylation of (3*S*)-3b

Following the general Procedure 2, compound (3*S*)-**3b** (289 mg, 0.69 mmol) in THF (40 mL) was treated with a solution of BF_3·THF (1 M in THF, 3.6 mL, 3.6 mmol). After oxidative workup, the crude mixture was chromatographed (silica gel, hexane/ethyl acetate 2:1) to yield compounds **8** (106 mg, 35%, first eluted) as colourless crystals and **9** (175 mg, 58%) as a colourless oil.

(3*S*,4*S*,5*S*,3'a*S*,4'*S*,6'a*S*)-2-(2',2'-Dimethyltetrahydrofuro[3,4-*d*][1,3]dioxol-4'-yl)-4-(2-trimethylsilylethoxy)-3-phenyl-[1,2]oxazinan-5-ol (8):

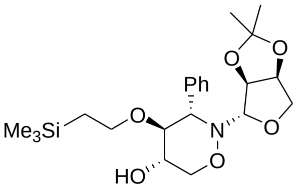

mp 121-125 °C; $[\alpha]_D^{22}$ = +131.8 (*c* 1.35, $CHCl_3$); ^1H NMR ($CDCl_3$, 500 MHz): δ = –0.22 (s, 9H, $SiMe_3$), 0.50 (ddd, *J* = 5.4, 11.7, 13.5 Hz, 1H, CH_2Si), 0.69 (ddd, *J* = 5.5, 11.9, 13.5 Hz, 1H, CH_2Si), 1.29, 1.35 (2 s, 3H each, 2 Me), 2.36 (d_{br}, *J* = 3.8 Hz, 1H, OH), 2.72 (ddd, *J* = 5.4, 9.5, 11.9 Hz, 1H, 4-OCH_2), 3.11 (ddd, *J* = 5.5, 9.5, 11.7 Hz, 1H, 4-OCH_2), 3.47 (dd_{br}, *J* ≈ 8.2, 9.4 Hz, 1H, 4-H), 3.63–3.71 (m, 2H, 5-H, 6-H), 3.92 (d, *J* = 9.4 Hz, 1H, 3-H), 3.94 (d, *J* = 9.4 Hz, 1H, 6'-H), 4.08 (dd_{br}, *J* ≈ 4.0, 9.5 Hz, 1H, 6-H), 4.19 (dd, *J* = 4.4, 9.4 Hz, 1H, 6'-H), 4.46 (s, 1H, 4'-H), 4.81 (dd, *J* = 4.4, 6.1 Hz, 1H, 6a'-H), 4.87 (d, *J* = 6.1 Hz, 1H, 3a'-H), 7.26–7.34, 7.37–7.42 (2 m, 5H, Ph) ppm; ^{13}C NMR ($CDCl_3$, 126 MHz): δ = –1.7 (q, $SiMe_3$), 18.7 (t, CH_2Si), 24.5,

26.3 (2 q, 2 Me), 67.9 (d, C-3), 69.8 (t, 4-OCH_2-), 70.6 (d, C-5), 71.4 (t, C-6), 77.4 (t, C-6'), 81.4 (d, C-6a'), 84.5 (d, C-3a'), 85.6 (d, C-4), 94.9 (d, C-4'), 111.7 (s, C-2'), 128.3, 128.7*, 137.1 (2 d, s, Ph) ppm, *higher intensity; IR (ATR): $\tilde{\nu}$ = 3400 (O-H), 3035–2870 (=C-H, C-H), 1050 (C-O) cm^{-1}; ESI-TOF (*m*/*z*): calcd. for $C_{22}H_{35}NNaO_6Si$ $[M + Na]^+$: 460.2131; found: 460.2149; Anal. calcd. for $C_{22}H_{35}NO_6Si$ (437.6): C 60.38, H 8.06, N 3.20; found: C 60.57, H 8.10, N 3.21.

(3*S*,4*R*,5*R*,3'a*S*,4'*S*,6'a*S*)-2-(2',2'-Dimethyltetrahydrofuro[3,4-*d*][1,3]dioxol-4'-yl)-4-(2-trimethylsilylethoxy)-3-phenyl-[1,2]oxazinan-5-ol (9):

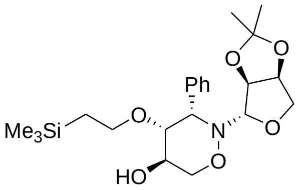

$[\alpha]_D^{22}$ = +122.6 (*c* 1.12, $CHCl_3$); ^{1}H NMR ($CDCl_3$, 500 MHz): δ = –0.11 (s, 9H, $SiMe_3$), 0.67 (ddd, *J* = 4.9, 10.9, 13.8 Hz, 1H, CH_2Si), 0.76 (ddd, *J* = 6.3, 11.4, 13.8 Hz, 1H, CH_2Si), 1.29, 1.36 (2 s, 3H each, 2 Me), 2.30 (s_{br}, 1H, OH), 2.95 (ddd, *J* = 4.9, 9.5, 11.4 Hz, 1H, 4-OCH_2), 3.27 (m_c, 1H, 4-H), 3.31 (ddd, *J* = 6.3, 9.5, 10.9 Hz, 1H, 4-OCH_2), 3.70 (s_{br}, 1H, 5-H), 3.82 (d, *J* = 12.1 Hz, 1H, 6-H), 3.95 (d, *J* = 9.4 Hz, 1H, 6'-H), 4.21 (dd, *J* = 4.6, 9.4 Hz, 1H, 6'-H), 4.40 (dd, *J* = 1.2, 12.1 Hz, 1H, 6-H), 4.44 (d, *J* = 2.4 Hz, 1H, 3-H), 4.64 (s, 1H, 4'-H), 4.82 (t_{br}, *J* ≈ 5.2 Hz, 1H, 6a'-H), 4.96 (d, *J* = 6.1 Hz, 1H, 3a'-H), 7.24–7.31, 7.43–7.46 (2 m, 5H, Ph) ppm; ^{13}C NMR ($CDCl_3$, 126 MHz): δ = –1.5 (q, $SiMe_3$), 18.3 (t, CH_2Si), 24.6, 26.3 (2 q, 2 Me), 62.8 (d, C-3), 66.4 (d, C-5), 69.1 (t, 4-OCH_2-), 71.1 (t, C-6), 77.6 (t, C-6'), 78.2 (d, C-4), 81.2 (d, C-6a'), 84.6 (d, C-3a'), 95.7 (d, C-4'), 111.6 (s, C-2'), 127.8, 128.1, 129.5, 136.6 (3 d, s, Ph) ppm; IR (ATR): $\tilde{\nu}$ = 3450 (O-H), 3065–2845 (=C-H, C-H), 1090, 1065 (C-O) cm^{-1}; ESI-TOF (*m*/*z*): calcd. for $C_{22}H_{35}NNaO_6Si$ $[M + Na]^+$: 460.2131; found: 460.2128;

Anal. calcd. for $C_{22}H_{35}NO_6Si$ (437.6): C 60.38, H 8.06, N 3.20; found: C 60.36, H 8.09, N 3.25.

Hydroxylation of (3*R*)-3a

By the procedure similar to that for (3*S*)-**3a** (Procedure 2), compound (3*R*)-**3a** (810 mg, 2.43 mmol) in THF (60 mL) was treated with a solution of BF_3·THF (1 M in THF, 9.8 mL, 9.8 mmol). After standard oxidative workup, the crude mixture was chromatographed (silica gel, hexane/ethyl acetate 1:1) to yield an enriched fraction of **10** (186 mg, first eluted) and pure **11** (568 mg, 66%). Additional chromatography purification of **10** (silica gel, dichloromethane/acetone 9:1) yielded an analytically pure sample (109 mg, 13%). Both alcohols were isolated as colourless oils.

(3*R*,4*R*,5*R*,3a'*S*,4'*S*,6a'*S*)-2-(2',2'-Dimethyltetrahydrofuro[3,4-*d*][1,3]dioxol-4'-yl)-4-methoxy-3-phenyl-[1,2]oxazinan-5-ol (10):

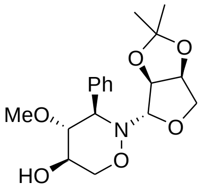

$[\alpha]_D^{22}$ = –79.4 (*c* 1.08, $CHCl_3$); ^{1}H NMR ($CDCl_3$, 500 MHz): δ = 1.29, 1.32 (2 s, 3H each, 2 Me), 2.70 (d, *J* = 2.1 Hz, 1H, OH), 2.89 (s, 3H, OMe), 3.37 (t, *J* ≈ 8.8 Hz, 1H, 4-H), 3.64 (d, *J* = 9.1 Hz, 1H, 3-H), 3.63-3.69 (m_{br}, 1H, 5-H), 3.79 (t, *J* ≈ 10.9 Hz, 1H, 6-H), 3.88 (d, *J* = 9.6 Hz, 1H, 6'-H), 4.04 (dd, *J* = 5.7, 11.2 Hz, 1H, 6-H), 4.17 (dd, *J* = 4.3, 9.6 Hz, 1H, 6'-H), 4.45 (s, 1H, 4'-H), 4.75 (dd, *J* = 4.3, 6.1 Hz, 1H, 6a'-H), 4.99 (d, *J* = 6.1 Hz, 1H, 3a'-H), 7.27–7.37, 7.41–7.45 (2 m, 5H, Ph) ppm; ^{13}C NMR ($CDCl_3$, 126 MHz): δ = 24.7, 26.3 (2 q, 2 Me), 60.8 (q, OMe), 69.6 (d, C-3), 70.6* (t, C-6, and d, C-5), 75.5 (t, C-6'), 78.8 (d, C-3a'), 81.2 (d, C-6a'), 87.8 (d, C-4), 96.5 (d, C-4'),

111.9 (s, C-2’), 128.5, 129.0*, 136.8 (2 d, s, Ph) ppm, *higher intensity; IR (ATR): $\tilde{\nu}$ = 3455 (O-H), 3035–2835 (=C-H, C-H), 1105, 1055 (C-O) cm^{-1}; Anal. calcd. for $C_{18}H_{25}NO_6$ (351.4): C 61.52, H 7.17, N 3.99; found: C 61.53, H 7.19, N 3.79.

(3*R*,4*S*,5*S*,3a’*S*,4’*S*,6a’*S*)-2-(2’,2’-Dimethyltetrahydrofuro[3,4-*d*][1,3]dioxol-4’-yl)-4-methoxy-3-phenyl-[1,2]oxazinan-5-ol (11):

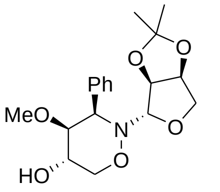

$[\alpha]_D^{22}$ = +4.4 (*c* 0.97, $CHCl_3$); ^{1}H NMR ($CDCl_3$, 500 MHz): δ = 1.30, 1.34 (2 s, 3H each, 2 Me), 2.32 (d, *J* = 5.4 Hz, 1H, OH), 3.14 (s, 3H, OMe), 3.30 (t_{br}, *J* ≈ 4.1 Hz, 1H, 4-H), 3.77 (dd, *J* = 4.5, 11.9 Hz, 1H, 6-H), 3.81 (d, *J* = 9.7 Hz, 1H, 6’-H), 3.89 (m_c, 1H, 5-H), 4.08 (dd, *J* = 4.3, 9.7 Hz, 1H, 6’-H), 4.28 (d, *J* = 3.5 Hz, 1H, 3-H), 4.38 (dd, *J* = 2.8, 11.9 Hz, 1H, 6-H), 4.67 (s, 1H, 4’-H), 4.76 (dd, *J* = 4.3, 6.1 Hz, 1H, 6a’-H), 4.98 (d, *J* = 6.1 Hz, 1H, 3a’-H), 7.26–7.33, 7.44–7.48 (2 m, 5H, Ph) ppm; ^{13}C NMR ($CDCl_3$, 126 MHz): δ = 24.7, 26.3 (2 q, 2 Me), 58.7 (q, OMe), 64.4 (d, C-3), 65.3 (d, C-5), 70.7 (t, C-6), 75.3 (t, C-6’), 80.6 (d, C-3a’), 81.1 (d, C-6a’), 81.3 (d, C-4), 97.8 (d, C-4’), 111.8 (s, C-2’), 127.7, 128.1, 129.2, 137.1 (3 d, s, Ph) ppm; IR (ATR): $\tilde{\nu}$ = 3440 (O-H), 3090–2830 (=C-H, C-H), 1095, 1050 (C-O) cm^{-1}; ESI-TOF (*m*/*z*): calcd. for $C_{18}H_{25}NNaO_6$ $[M + Na]^+$: 374.1580; found: 374.1577; Anal. calcd. for $C_{18}H_{25}NO_6$ (351.4): C 61.52, H 7.17, N 3.99; found: C 61.47, H 7.15, N 3.88.

(3*S*,4*R*,5*R*)-4-Methoxy-3-phenyl-[1,2]oxazinan-5-ol (13):

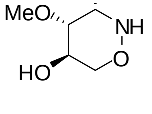

By a procedure similar to that for **6** (Procedure 3), 1,2-oxazine **7** (255 mg, 0.72 mmol) was dissolved in 1N HCl in MeOH (8 mL) and heated at 40 °C for 4 h (TLC monitoring, hexane/AcOEt 1:2, potassium permanganate stain). After workup and purification on a column (silica gel, dichloromethane/methanol 40:1) compound **13** (127 mg, 83%) was isolated as a colourless solid.

mp 112-113 °C; $[\alpha]_D^{22}$ = +47.9 (*c* 1.00, $CHCl_3$); ^{1}H NMR ($CDCl_3$, 500 MHz): δ = 3.19 (s, 3H, OMe), 3.40 (t_{br}, *J* = 1.5 Hz, 1H, 4-H), 3.87 (ddd_{br}, *J* = 12.1, 2.1, 0.9 Hz, 1H, 6-H), 3.89 (dd, *J* = 3.7, 2.2 Hz, 1H, 5-H), 4.25 (dd, *J* = 12.1, 1.1 Hz, 1H, 6-H), 4.58 (d, *J* = 2.0 Hz, 1H, 3-H), 7.27–7.36 (m, 5H, Ph) ppm; ^{13}C NMR ($CDCl_3$, 126 MHz): δ = 59.0 (q, OMe), 60.3 (d, C-3), 65.2 (d, C-5), 71.2 (t, C-6), 78.2 (d, C-4), 127.5, 127.6, 128.3, 137.3 (3d, s, Ph) ppm; IR (ATR): $\tilde{\nu}$ = 3390–3220 (O-H, N-H), 3060–2830 (=C-H, C-H), 1090 (C-O) cm^{-1}; ESI-TOF (*m*/*z*): calcd. for $C_{11}H_{13}NO_3$ $[M + H]^+$: 210.1130; found: 210.1128; Anal. calcd. for $C_{11}H_{15}NO_3$ (209.2): C 63.14, H 7.23, N 6.69; found: C 63.15, H 7.13, N 6.72.

(3*S*,4*S*,5*S*)-3-Phenyl-[1,2]oxazine-4,5-diol (14):

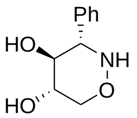

To a solution of 1,2-oxazine **8** (100 mg, 0.23 mmol) in EtOH (8.0 mL) acidic ion exchange resin DOWEX-50 (1.26 g; washed several times with EtOH before usage) was added. The suspension was vigorously stirred at 50 °C for 10 d, and then decanted. The resin was washed with three portions of ammonia in methanol solution (7 N, *ca*. 5.0 mL each) and the organics combined. After evaporation of the solvents, compound **14** (35 mg, 78%) was isolated as a colourless solid.

mp 153-154 °C; $[\alpha]_D^{22}$ = +33.8 (*c* 1.05, CH_3OH); ^{1}H NMR (CD_3OD, 500 MHz, 50 °C): δ = 3.60–3.69 (m, 3H, 4-H, 5-H, 6-H), 3.85 (d, *J* = 9.2 Hz, 1H, 3-H), 4.02–4.08 (m, 1H, 6-H), 7.27–7.31, 7.32–7.36, 7.38–7.41 (3 m, 5H, Ph) ppm; ^{13}C NMR (CD_3OD, 126 MHz, 50 °C): δ = 69.2 (d, C-3), 72.6 (d, C-5), 74.3 (t, C-6), 77.1 (d, C-4), 129.2, 129.5, 129.9, 137.6 (3 d, s, Ph) ppm; IR (neat): $\tilde{\nu}$ = 3460, 3150 (O-H, N-H), 3085–2885 (=C-H, C-H), 1030 (C-O) cm^{-1}; ESI-TOF (*m*/*z*): calcd. for $C_{10}H_{13}NNaO_3$ [M + Na]$^+$: 218.0793; found: 218.0843; Anal. calcd. for $C_{10}H_{13}NO_3$ (195.2): C 61.53, H 6.71, N 7.18; found: C 61.45, H 6.67, N 7.13.

Synthesis of 14 by demethylation of 12

To a solution of 1,2-oxazine **12** (59 mg, 0.28 mmol) in CH_2Cl_2 (4.4 mL) a solution of BBr_3 (1 M in CH_2Cl_2, 0.85 mL, 0.85 mmol) was slowly added at –78 °C and stirred for 1 h, and then at room temperature overnight. The mixture was diluted with MeOH (3 mL) and the solvents were removed. The crude mixture was flash chromatographed (silica gel, dichloromethane/methanol 20:1) to give an enriched fraction of **14** (33 mg). Additional purification on a column (silica gel, dichloromethane/methanol 25:1) furnished **14** (10 mg, 18%) as a colourless solid.

(3*S*,4*R*,5*R*)-3-Phenyl-[1,2]oxazine-4,5-diol (15):

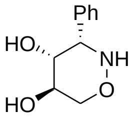

Following the general Procedure 3, compound **9** (160 mg, 0.37 mmol) in 1 N HCl in MeOH (10 mL) was heated overnight at 40 °C. The crude product was purified on a column (silica gel, dichloromethane/methanol 30:1) to give *N*-unsubstituted 1,2-oxazine derivative (89 mg, 82%) as a colourless oil. $[\alpha]_D^{22}$ = 25.7 (*c* 0.68, $CHCl_3$); ^{1}H

NMR ($CDCl_3$, 500 MHz): δ = –0.12 (s, 9H, $SiMe_3$), 0.68 (ddd, *J* = 5.6, 10.5, 13.8 Hz, 1H, CH_2Si), 0.74 (ddd, *J* = 6.3, 10.7, 13.8 Hz, 1H, CH_2Si), 3.07 (ddd, *J* = 5.6, 9.7, 10.6 Hz, 1H, 4-OCH_2), 3.40 (ddd, *J* = 6.3, 9.7, 10.5 Hz, 1H, 4-OCH_2), 3.43 (m_c, 1H, 4-H), 3.81 (m_c, 1H, 5-H), 3.86 (d_{br}, *J* ≈ 12.4 Hz, 1H, 6-H), 4.23 (dd, *J* = 1.2, 12.4 Hz, 1H, 6-H), 4.58 (d, *J* = 1.9 Hz, 1H, 3-H), 7.24–7.30 (m, 5H, Ph) ppm; ^{13}C NMR ($CDCl_3$, 126 MHz): δ = –1.5 (q, $SiMe_3$), 18.4 (t, CH_2Si), 60.1 (d, C-3), 66.0 (d, C-5), 68.7 (t, 4-OCH_2), 71.4 (t, C-6), 76.1 (d, C-4), 127.40, 127.44, 128.1, 137.6 (3 d, s, Ph) ppm; IR (ATR): $\tilde{\nu}$ = 3485–3230 (O-H, N-H), 3090–2850 (=C-H, C-H), 1245, 1085 (C-O) cm^{-1}; ESI-TOF (*m*/*z*): calcd. for $C_{15}H_{25}NO_3Si$ $[M + Na]^+$: 318.1501; found: 318.1504. To a solution of this partially deprotected 1,2-oxazine (80 mg, 0.27 mmol) in EtOH (7.0 mL), acidic ion exchange resin DOWEX-50 (533 mg; washed several times with EtOH before usage) was added. The suspension was vigorously stirred at 50 °C for 4 d, and then decanted. The resin was washed with three portions of ammonia in methanol solution (7 N, *ca*. 4.0 mL each) and the organics were combined. After evaporation of the solvents compound **15** (49 mg, 92%) was isolated as a colourless solid.

mp 190-192 °C; $[\alpha]_D^{22}$ = +65.6 (*c* 1.26, CH_3OH); ^1H NMR (CD_3OD, 500 MHz): δ = 3.70 (dt, *J* ≈ 1.7, 3.7 Hz, 1H, 5-H), 3.74 (m_c, 1H, 4-H), 3.89 (ddd_{br}, *J* ≈ 0.9, 1.7, 12.5 Hz, 1H, 6-H), 4.25 (dd, *J* = 1.5, 12.5 Hz, 1H, 6-H), 4.57 (d, *J* = 1.6 Hz, 1H, 3-H), 7.24–7.27, 7.30–7.35 (2 m, 5H, Ph) ppm; ^{13}C NMR (CD_3OD, 126 MHz): δ = 61.7 (d, C-3), 69.3 (d, C-5), 69.5 (d, C-4), 71.3 (t, C-6), 128.3, 128.7, 129.2, 139.6 (3 d, s, Ph) ppm; IR (neat): $\tilde{\nu}$ = 3480, 3170 (O-H, N-H), 3065–2850 (=C-H, C-H), 1070, 1005 (C-O) cm^{-1}; ESI-TOF (*m*/*z*): calcd. for $C_{10}H_{13}NNaO_3$ $[M + Na]^+$: 218.0793; found: 218.0787; Anal. calcd. for $C_{10}H_{13}NO_3$ (195.2): C 61.53, H 6.71, N 7.18; found: C 61.57, H 6.69, N 7.18.

(3*R*,4*R*,5*R*)-4-Methoxy-3-phenyl-[1,2]oxazinan-5-ol (*ent*-12):

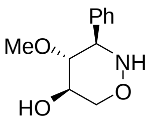

Following the Procedure 4 compound **10** (60 mg, 0.17 mmol) was dissolved in 1 N HCl in MeOH (2 mL). Standard workup and purification on a column (silica gel, dichloromethane/methanol 40:1) furnished *ent*-**12** (30 mg, 84%) as a colourless solid. mp 110-112 °C; $[\alpha]_D^{22}$ = –61.5 (*c* 1.18, $CHCl_3$); The spectroscopic data correspond with those of compound **12**. Anal. calcd. for $C_{11}H_{15}NO_3$ (209.2): C 63.14, H 7.23, N 6.69; found: C 63.12, H 7.24, N 6.61.

(3*R*,4*S*,5*S*)-4-Methoxy-3-phenyl-[1,2]oxazinan-5-ol (*ent*-13):

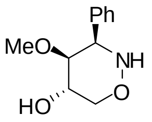

Following the Procedure 4 compound **11** (289 mg, 0.82 mmol) was dissolved in methanolic HCl (1 N, 9.5 mL). Standard workup and purification on a column (silica gel, dichloromethane/methanol 40:1) yielded *ent*-**13** (159 mg, 92%) as a colourless solid.

mp 110-113 °C; $[\alpha]_D^{22}$ = –48.8 (*c* 1.10, $CHCl_3$); The spectroscopic data correspond with those of compound **13**. Anal. calcd. for $C_{11}H_{15}NO_3$ (209.2): C 63.14, H 7.23, N 6.69; found: C 63.17, H 7.32, N 6.54.

(2*R*,3*R*,4*S*)-4-Amino-3-methoxy-4-phenylbutane-1,2-diol (17):

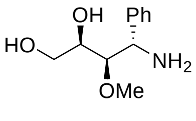

Following the Procedure 4 compound **13** (76 mg, 0.36 mmol) in THF (4 mL) was reacted with SmI_2 (*ca*. 0.1 M solution in THF, 11 mL, ~1.1 mmol). After workup and filtration through a short silica gel pad (dichloromethane/methanol 15:1), compound **17** (73 mg, 95%) was isolated as a colourless oil.

$[\alpha]_D^{22}$ = +17.1 (*c* 1.12, MeOH); ^{1}H NMR ($CDCl_3/CD_3OD$ 20:1, 500 MHz): δ = 3.31 (s, 3H, OMe), 3.58 (t_{br}, $J \approx 3.3$ Hz, 1H, 3-H), 3.62 (dd, J = 4.8, 11.5 Hz, 1H, 1-H), 3.69 (dd, J = 5.6, 11.5 Hz, 1H, 1-H), 3.88 (td, $J \approx 2.8$, 5.2 Hz, 1H, 2-H), 4.57 (d, J = 3.7 Hz, 1H, 4-H), 7.30–7.39, 7.46–7.49 (2 m, 5H, Ph) ppm; ^{13}C NMR ($CDCl_3/CD_3OD$ 20:1, 126 MHz): δ = 56.1 (d, C-4), 61.5 (q, OMe), 62.0 (t, C-1), 71.6 (d, C-2), 82.2 (d, C-3), 127.1, 128.8, 129.1, 136.7 (3 d, s, Ph) ppm; IR (ATR): $\tilde{\nu}$ = 3530–3245 (O-H, N-H), 3035–2855 (=C-H, C-H), 1080 (C-O) cm^{-1}; ESI-TOF (*m*/*z*): calcd. for $C_{11}H_{18}NO_3$ $[M + H]^+$: 212.1287; found: 212.1282.

(3*S*,4*R*,5*R*,3a'*S*,4'*S*,6a'*S*)-5-Benzyloxy-2-(2',2'-dimethyltetrahydrofuro[3,4-*d*][1,3]dioxol-4'-yl)-4-methoxy-3-phenyl-[1,2]oxazine (18):

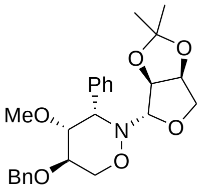

A solution of **7** (261 mg, 0.74 mmol) in DMF (3 mL) was added to NaH (60% in mineral oil; 46 mg, 1.04 mmol) at 0 °C. Benzyl bromide (160 mg, 0.89 mmol) was added and the solution was stirred at room temperature overnight. Then H_2O (6 mL) was added, the aqueous layer was extracted with Et_2O (3 × 25 mL), and the combined organics dried with $MgSO_4$. After filtration, the solvent was removed in

vacuo. The crude product was purified by column chromatography (silica gel, hexane/ethyl acetate, 5:1) to give compound **18** as a colourless oil (278 mg, 85%). $[\alpha]_D^{22}$ = +111.3 (*c* 0.83, $CHCl_3$); ^{1}H NMR ($CDCl_3$, 500 MHz): δ = 1.29, 1.36 (2 s, 3H each, 2 Me), 3.08 (s, 3H, OMe), 3.27 (m_c, 1H, 4-H), 3.48 (m_c, 1H, 5-H), 3.94 (d, *J* = 9.3 Hz, 1H, 6'-H), 4.00 (d_{br}, *J* ≈ 12.6 Hz, 1H, 6-H), 4.23 (dd, *J* = 1.6, 12.6 Hz, 1H, 6-H), 4.31 (dd, *J* = 4.6, 9.3 Hz, 1H, 6'-H), 4.54 (d, *J* = 2.1 Hz, 1H, 3-H), 4.62 (d, *J* = 12.3 Hz, 1H, Bn), 4.63 (s, 1H, 4'-H), 4.69 (d, *J* = 12.3 Hz, 1H, Bn), 4.81 (t_{br}, *J* ≈ 5.2 Hz, 1H, 6a'-H), 4.96 (d, *J* = 6.1 Hz, 1H, 3a'-H), 7.24–7.33, 7.35–7.41, 7.45–7.48 (3 m, 10H, 2 Ph) ppm; ^{13}C NMR ($CDCl_3$, 126 MHz): δ = 24.5, 26.3 (2 q, 2 Me), 59.1 (q, OMe), 63.2 (d, C-3), 68.0 (t, C-6), 70.8 (t, Bn), 71.6 (d, C-5), 77.6 (t, C-6'), 78.8 (d, C-4), 81.4 (d, C-6a'), 84.6 (d, C-3a'), 95.6 (d, C-4'), 111.4 (s, C-2'), 127.5, 127.7, 127.8, 128.2, 128.5, 129.6, 137.0, 138.2 (6 d, 2 s, 2 Ph) ppm; IR (ATR): $\tilde{\nu}$ = 3090–2825 (=C-H, C-H), 1105, 1090, 1065, 1055 (C-O) cm^{-1}; ESI-TOF (*m*/*z*): calcd. for $C_{25}H_{31}NNaO_6$ $[M + Na]^+$: 464.2044; found: 464.2054; Anal. calcd. for $C_{25}H_{31}NO_6$ (441.5): C 68.01, H 7.08, N 3.17; found: C 68.23, H 7.24, N 2.99.

Reaction of 1,2-oxazine 18 with SmI_2

Following the general Procedure 4, compound **18** (105 mg, 0.24 mmol) in THF (3 mL) was added to SmI_2 solution (*ca*. 0.1 M in THF, 10 mL, ~1.0 mmol) and stirred overnight. After standard workup, the crude mixture was purified on column (silica gel, dichloromethane/methanol 40:1 gradient to 10:1) to give unconsumed **18** (5 mg, 5%), an enriched fractions of **19** (40 mg), and diol **20** (18 mg, 17%). Additional purification of the second fraction (silica gel, hexane/ethyl acetate 1:2) yielded compound **20** (19 mg, 18%). All compounds were isolated as colourless oils.

(2*R*,3*R*,3a'*S*,4*S*,4'*S*,6a'*S*)-2-Benzyloxy-4-(2,2-dimethyltetrahydrofuro[3,4-d][1,3]dioxol-ylamino)-3-methoxy-4-phenylbutan-1-ol (19):

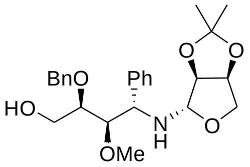

$[\alpha]_D^{22}$ = +66.8 (*c* 1.06, $CHCl_3$); ^{1}H NMR ($CDCl_3$, 500 MHz): δ = 1.27, 1.38 (2 s, 3H each, 2 Me), 3.29 (s, 3H, OMe), 3.49 (dd, *J* = 3.1, 5.5 Hz, 1H, 3-H), 3.62 (dt, *J* ≈ 4.2, 5.5 Hz, 1H, 2-H), 3.73 (dd, *J* = 3.7, 12.1 Hz, 1H, 1-H), 3.78 (dd, *J* = 4.5, 12.1 Hz, 1H, 1-H), 3.95 (d, *J* = 10.6 Hz, 1H, 6'-H), 4.00 (dd, *J* = 3.6, 10.6 Hz, 1H, 6'-H), 4.17 (d, *J* = 3.1 Hz, 1H, 4-H), 4.43 (d, *J* = 6.0 Hz, 1H, 3a'-H), 4.47 (s, 1H, 4'-H), 4.58, 4.67 (2 d, *J* = 11.7 Hz, 2H, Bn), 4.81 (dd, *J* = 3.6, 6.0 Hz, 1H, 6a'-H), 7.27–7.36 (m, 10H, 2 Ph) ppm; ^{13}C NMR ($CDCl_3$, 126 MHz): δ = 24.6, 26.1 (2 q, 2 Me), 56.4 (d, C-4), 60.1 (t, C-1), 60.2 (q, OMe), 71.4 (t, C-6'), 72.2 (t, Bn), 78.1 (d, C-2), 80.7 (d, C-6a'), 85.4 (d, C-3), 85.8 (d, C-3a'), 91.7 (d, C-4'), 112.2 (s, C-2'), 127.5, 127.67, 127.73, 127.8, 128.4, 128.7, 138.3, 140.5 (6 d, 2 s, 2 Ph) ppm; IR (ATR): $\tilde{\nu}$ = 3455–3345 (O-H, N-H), 3090–2830 (=C-H, C-H), 1095, 1060 (C-O) cm^{-1}; ESI-TOF (*m*/*z*): calcd. for $C_{25}H_{34}NO_6$ $[M + H]^+$: 444.2381; found: 444.2372.

(2R,3R,4S,4'R,5'S)-2-Benzyloxy-4-[(5-hydroxymethyl-2,2-dimethyl-[1,3]dioxolan-4-ylmethyl)amino]-3-methoxy-4-phenylbutan-1-ol (20):

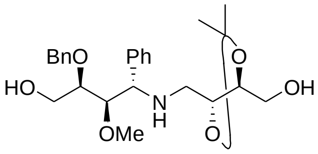

$[\alpha]_D^{22}$ = +38.5 (*c* 1.07, $CHCl_3$); ^{1}H NMR ($CDCl_3$, 500 MHz): δ = 1.34, 1.43 (2 s, 3H each, 2 Me), 2.49 (dd, *J* = 5.8, 12.2 Hz, 1H, N-CH_2), 2.58 (dd, *J* = 4.6, 12.2 Hz, 1H, N-CH_2), 3.37 (s, 3H, OMe), 3.45 (dd_{br}, *J* ≈ 4.1, 8.2 Hz, 1H, 2-H), 3.47–3.52 (m, 2H, 3-

H, 5'-CH_2), 3.61 (dd, *J* = 5.2, 12.1 Hz, 1H, 5'-CH_2), 3.72–3.79 (m, 2H, 1-H), 3.97 (d, *J* = 4.6 Hz, 1H, 4-H), 4.17 (dt, *J* ≈ 4.9, 6.7 Hz, 1H, 5'-H), 4.26 (td, *J* ≈ 4.8, 5.8 Hz, 1H, 4'-H), 4.49, 4.68 (2 d, *J* = 11.8 Hz, 2H, Bn), 7.27–7.36 (m, 10H, 2 Ph) ppm; ^{13}C NMR ($CDCl_3$, 126 MHz): δ = 25.0, 27.4 (2 q, 2 Me), 46.1 (t, N-CH_2), 59.7 (t, C-1), 60.49 (t, 5'-CH_2), 60.53 (q, OMe), 61.83 (d, C-4), 71.8 (t, Bn), 75.8 (d, C-4'), 77.32 (d, C-2), 77.34 (d, C-5'), 85.7 (d, C-3), 108.2 (s, C-2'), 127.70, 127.71, 127.74, 127.76, 128.4, 128.7, 138.3, 140.2 (6 d, 2 s, 2 Ph) ppm; IR (ATR): $\tilde{\nu}$ = 3465–3300 (O-H, N-H), 3090–2825 (=C-H, C-H), 1090, 1050 (C-O) cm^{-1}; ESI-TOF (*m*/*z*): calcd. for $C_{25}H_{36}NO_6$ [M + H]$^+$: 446.2543; found: 446.2564.

(3*S*,4*R*,5*R*)-4-Methoxy-3-phenyl-5-trimethylsilanyloxy-[1,2]oxazinane (21):

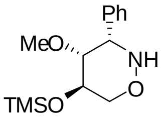

To a solution of 1,2-oxazine **13** (70 mg, 0.33 mmol) in dry CH_2Cl_2 (1.5 mL) were added dropwise at 0 °C imidazole (36 mg, 0.52 mmol), DMAP (2 mg, 5 mol%) and TMSCl (63 µL, 54 mg, 0.50 mmol) in CH_2Cl_2 (3 mL). The reaction mixture was allowed to reach room temperature and stirred overnight. Then CH_2Cl_2 (2 mL) was added followed by H_2O (2 mL), and the layers were separated. The aqueous layer was extracted with three portions of CH_2Cl_2 (5 mL each), the combined organics were washed with brine, dried over Na_2SO_4, and then filtered, and the solvents were removed under reduced pressure. Purification by column chromatography (silica gel, hexane/ethyl acetate 2:1) yielded **21** (85 mg, 90%) as a colourless oil.

$[\alpha]_D^{22}$ = –13.8 (*c* 1.35, THF); ^{1}H NMR ($CDCl_3$, 400 MHz): δ = 0.20 (s, 9H, $SiMe_3$), 3.22 (s, 3H, OMe), 3.25 (m_c, 1H, 4-H), 3.78 (dt_{br}, *J* ≈ 1.4, 12.3 Hz, 1H, 6-H), 3.87 (dt, *J* ≈ 1.7, 3.5 Hz, 1H, 5-H), 4.16 (dd, *J* = 1.4, 12.3 Hz, 1H, 6-H), 4.63 (d, *J* = 1.6 Hz, 1H, 3-

H), 7.25–7.38 (m, 5H, Ph) ppm; ^{13}C NMR ($CDCl_3$, 126 MHz): δ = 0.05 (q, $SiMe_3$), 58.9 (q, OMe), 60.0 (d, C-3), 65.4 (d, C-5), 71.3 (t, C-6), 78.9 (d, C-4), 127.4, 127.6, 128.2, 137.9 (3 d, s, Ph) ppm; IR (ATR): $\tilde{\nu}$ = 3280–3225 (N-H), 3090–2825 (=C-H, C-H), 1250, 1090 (C-O) cm^{-1}; ESI-TOF (*m/z*): calcd. for $C_{14}H_{23}NNaO_3Si$ $[M + Na]^+$: 304.1345; found: 304.1339; Anal. calcd. for $C_{14}H_{23}NO_3Si$ (281.4): C 59.75, H 8.24, N 4.98; found: C 59.73, H 8.26, N 4.98.

Reaction of 21 with SmI_2 and attempted cyclisation with MsCl

Following the general Procedure 4, compound **21** (168 mg, 0.60 mmol) in THF (5 mL) was added to a solution of SmI_2 (*ca*. 0.1 M in THF, 18 mL, ~1.8 mmol). After workup and drying over $MgSO_4$, the crude product was filtered and the solvents were removed to give a colourless oil (160 mg). This was dissolved in CH_2Cl_2 (30 mL), triethylamine (1 mL, 7.0 mmol) and methanesulfonyl chloride (150 µL, 1.94 mmol) was slowly added at 0 °C. The reaction mixture was allowed to reach room temperature and stirred overnight, then quenched with H_2O and extracted with several portions of CH_2Cl_2, and the combined organics were dried ($MgSO_4$). Crude products were separated on a column (silica gel, hexane/ethyl acetate) to give compounds **23** (16 mg, 7%), **24** (63 mg, 25%), **25** (10 mg, 5%) as colourless oils and compound **26** (68 mg, 35%) as a colourless solid.

(2*R*,3*R*,4*S*)-4-methylsulfonylamino-3-methoxy-4-phenyl-1,2-*O*-bis(trimethylsilyl)-butan-1,2-diol (23):

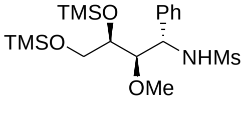

$[\alpha]_D^{22}$ = +36.9 (*c* 1.24, $CHCl_3$); ^1H NMR ($CDCl_3$, 500 MHz): δ = 0.12, 0.14 (2 s, 9H each, 2 $SiMe_3$), 2.56 (s, 3H, NMs), 3.26 (s, 3H, OMe), 3.43 (dd, *J* = 3.1, 5.9 Hz, 1H, 3-H), 3.66 (dd, *J* = 3.0, 10.3 Hz, 1H, 1-H), 3.79-3.85 (m, 2H, 1-H, 2-H), 4.72 (dd, *J* = 3.1, 8.3 Hz, 1H, 4-H), 5.41 (d, *J* = 8.3 Hz, 1H, NH), 7.29–7.40 (m, 5H, Ph) ppm; ^{13}C NMR ($CDCl_3$, 126 MHz): δ = –0.5, 0.3, (2 q, 2 $SiMe_3$), 42.0 (q, NMs), 57.1 (d, C-4), 61.2 (q, OMe), 64.1 (t, C-1), 73.9 (d, C-2), 85.4 (d, C-3), 127.0, 127.9, 128.8, 140.2 (3 d, s, Ph) ppm; IR (ATR): $\tilde{\nu}$ = 3290 (N-H), 3065–2830 (=C-H, C-H), 1320 (S=O), 1250, 1150, 1085 (C-O) cm^{-1}; ESI-TOF (*m*/*z*): calcd. for $C_{18}H_{35}NNaO_5SSi_2$ $[M + Na]^+$: 456.1672; found: 456.1678.

(2*R*,3*R*,4*S*)-2-*O*-Methylsulfonyl-4-methylsulfonylamino-3-methoxy-4-phenyl-1-*O*-trimethylsilylbutan-1,2-diol (24):

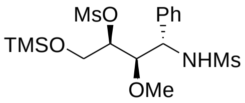

$[\alpha]_D^{22}$ = +28.8 (*c* 1.25, $CHCl_3$); ^1H NMR ($CDCl_3$, 500 MHz): δ = 0.19 (s, 9H, $SiMe_3$), 2.58 (s, 3H, NMs), 3.07 (s, 3H, OMs), 3.17 (s, 3H, OMe), 3.67 (dd, *J* = 1.7, 7.7 Hz, 1H, 3-H), 4.02 (dd, *J* = 2.1, 12.7 Hz, 1H, 1-H), 4.16 (dd, *J* = 4.4, 12.7 Hz, 1H, 1-H), 4.71 (dd, *J* = 1.7, 10.1 Hz, 1H, 4-H), 4.77 (ddd, *J* = 2.1, 4.4, 7.7 Hz, 1H, 2-H), 5.61 (d, *J* = 10.1 Hz, 1H, NH), 7.32–7.43 (m, 5H, Ph) ppm; ^{13}C NMR ($CDCl_3$, 126 MHz): δ = –0.6 (q, $SiMe_3$), 38.1 (q, OMs), 42.0 (q, NMs), 56.5 (d, C-4), 61.6 (q, OMe), 61.7 (t, C-1), 83.9 (d, C-3), 84.2 (d, C-2), 126.6, 128.3, 129.1, 139.0 (3 d, s, Ph) ppm; IR (ATR): $\tilde{\nu}$ = 3285 (N-H), 3060–2840 (=C-H, C-H), 1320 (S=O), 1175, 1155 (C-O) cm^{-1}; ESI-TOF (*m*/*z*): calcd. for $C_{16}H_{29}NNaO_7S_2Si$ $[M + Na]^+$: 462.1052; found: 462.1052.

(2*S*,3*R*,4*R*)-3-Methoxy-4-trimethylsilyloxy-1-methylsulfonyl-2-phenylpyrrolidine (25):

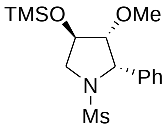

$[\alpha]_D^{22}$ = +98.5 (*c* 0.41, $CHCl_3$); ^{1}H NMR ($CDCl_3$, 500 MHz): δ = 0.19 (s, 9H, $SiMe_3$), 2.75 (s, 3H, NMs), 2.95 (s, 3H, OMe), 3.55 (dt, *J* ≈ 1.4, 11.9 Hz, 1H, 5-H), 3.65 (ddd, *J* = 0.9, 2.5, 5.1 Hz, 1H, 3-H), 3.78 (dd, *J* = 3.4, 11.9 Hz, 1H, 5-H), 4.24 (m_c, 1H, 4-H), 4.90 (d, *J* = 5.1 Hz, 1H, 2-H), 7.25–7.29, 7.31–7.39 (2 m, 5H, Ph) ppm; ^{13}C NMR ($CDCl_3$, 126 MHz): δ = –0.1 (q, $SiMe_3$), 36.4 (q, NMs), 55.3 (t, C-5), 58.9 (q, OMe), 65.7 (d, C-2), 73.4 (d, C-4), 88.6 (d, C-3), 127.5, 128.0, 128.1, 136.8 (3 d, s, Ph) ppm; IR (ATR): $\tilde{\nu}$ = 3065–2830 (=C-H, C-H), 1340 (S=O), 1155, 1110, 1020 (C-O) cm^{-1}; ESI-TOF (*m*/*z*): calcd. for $C_{15}H_{25}NNaO_4SSi$ [M + Na]$^+$: 366.1171; found: 366.1171.

(2*S*,3*R*,4*R*)-3-Methoxy-2-phenyl-4-methylsulfonyloxy-1-methylsulfonyl-pyrrolidine (26):

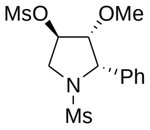

$[\alpha]_D^{22}$ = +57.1 (*c* 1.03, $CHCl_3$); ^{1}H NMR ($CDCl_3$, 500 MHz): δ = 2.66 (s, 3H, NMs), 3.06 (s, 3H, OMe), 3.14 (s, 3H, OMs), 3.89 (dd, *J* = 3.8, 12.9 Hz, 1H, 2-H), 4.07–4.11 (m, 2H, 2-H, 4-H), 5.06 (d, *J* = 5.5 Hz, 1H, 5-H), 5.12 (dt, *J* ≈ 2.3, 4.3 Hz, 1H, 3-H), 7.30–7.39 (m, 5H, Ph) ppm; ^{13}C NMR ($CDCl_3$, 126 MHz): δ = 39.1 (q, OMs), 40.1 (q, NMs), 51.6 (t, C-2), 59.1 (q, OMe), 65.0 (d, C-5), 79.6 (d, C-3), 85.6 (d, C-4), 128.26, 128.29, 128.4, 135.1 (3 d, s, Ph) ppm; IR (ATR): $\tilde{\nu}$ = 3020–2840 (=C-H, C-H), 1325

(S=O), 1175, 1145 (C-O) cm^{-1}; ESI-TOF (*m*/*z*): calcd. for $C_{13}H_{19}NNaO_6S_2$ [M + Na]$^+$: 372.0551; found: 372.0552; Anal. calcd. for $C_{13}H_{19}NO_6S_2$ (349.4): C 44.68, H 5.48, N 4.01; found: C 44.70, H 5.56, N 4.11.

(3*S*,4*R*,5*R*)-5-(*tert*-Butyldimethylsilyloxy)-4-methoxy-3-phenyl-[1,2]oxazinane (27):

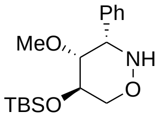

By a similar procedure to that for TMSCl, to 1,2-oxazine **13** (713 mg, 3.41 mmol) in dry CH_2Cl_2 (25 mL) were added dropwise at 0 °C imidazole (525 mg, 7.71 mmol), DMAP (21 mg, 0.17 mmol, 5 mol %) and TBSCl (1.035 g, 6.87 mmol) in CH_2Cl_2 (10 mL). The reaction mixture was stirred for five days at room temperature. After workup and purification through a column (silica gel, hexane/ethyl acetate 3:1) compound **27** (995 mg, 90%) was isolated as a colourless oil.

$[\alpha]_D^{22}$ = +4.0 (*c* 0.92, $CHCl_3$); ^{1}H NMR ($CDCl_3$, 500 MHz): δ = 0.136, 0.140 (2 s, 3H each, $SiMe_2$), 0.96 (s, 9H, Si^{t}Bu), 3.21 (s, 3H, OMe), 3.25 (m_c, 1H, 4-H), 3.78 (dt, *J* ≈ 1.5, 12.3 Hz, 1H, 6-H), 3.88 (dt, *J* ≈ 1.7, 3.5 Hz, 1H, 5-H), 4.13 (dd, *J* = 1.4, 12.3 Hz, 1H, 6-H), 4.64 (d, *J* = 1.8 Hz, 1H, 3-H), 7.25–7.33 (m, 5H, Ph) ppm; ^{13}C NMR ($CDCl_3$, 126 MHz): δ = –4.9, –4.7 (2 q, $SiMe_2$), 18.1, 25.8 (s, q, Si^{t}Bu), 58.9 (q, OMe), 60.0 (d, C-3), 65.6 (d, C-5), 71.3 (t, C-6), 78.9 (d, C-4), 127.4, 127.5, 128.2, 138.1 (3 d, s, Ph) ppm; IR (ATR): $\tilde{\nu}$ = 3290 (N-H), 3065-2825 (=C-H, C-H), 1255, 1120, 1095 (C-O) cm^{-1}; ESI-TOF (*m*/*z*): calcd. for $C_{17}H_{30}NO_3Si$ [M + H]$^+$: 324.1995; found: 324.2010.

(2*R*,3*R*,4S)-4-Amino-2-*O*-(*tert*-butyldimethylsilyl)-3-methoxy-4-phenyl-butane-1,2-diol (28):

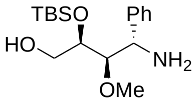

Following the general Procedure 4, compound **27** (500 mg, 1.54 mmol) in THF (25 mL) was added to a solution of SmI_2 (*ca*. 0.1 M in THF, 57 mL, ~5.7 mmol). After workup and filtration through a short silica gel pad (dichloromethane/methanol 15:1), compound **28** (463 mg, 92%) was isolated as a colourless oil.

$[\alpha]_D^{22}$ = +27.0 (*c* 0.97, $CHCl_3$); ^{1}H NMR ($CDCl_3$, 400 MHz): δ = 0.07, 0.12 (2 s, 3H each, $SiMe_2$), 0.92 (s, 9H, Si^{t}Bu), 3.27 (s, 3H, OMe), 3.37 (dd, *J* = 0.9, 5.6 Hz, 1H, 3-H), 3.57 (dd, *J* = 4.4, 12.3 Hz, 1H, 1-H), 3.83 (dd, *J* = 1.5, 12.3 Hz, 1H, 1-H), 3.91 (ddd, *J* = 1.5, 4.4, 5.6 Hz, 1H, 2-H), 4.46 (s_{br}, 1H, 4-H), 7.25–7.30, 7.32–7.41 (2 m, 5H, Ph) ppm; ^{13}C NMR ($CDCl_3$, 126 MHz): δ = –4.9, –4.6 (2 q, $SiMe_2$), 18.1, 25.9 (s, q, Si^{t}Bu), 52.1 (d, C-4), 59.7 (q, OMe), 61.2 (t, C-1), 70.6 (d, C-2), 87.0 (d, C-3), 126.2, 127.1, 128.6, 145.4 (3 d, s, Ph) ppm; IR (ATR): $\tilde{\nu}$ = 3460–3250 (O-H, N-H), 3065–2855 (=C-H, C-H), 1255, 1130, 1090 (C-O) cm^{-1}; ESI-TOF (*m*/*z*): calcd. for $C_{17}H_{32}NO_3Si$ $[M + H]^+$: 326.2146; found: 326.2137.

(2*S*,3*R*,4*R*)-4-(*tert*-Butyldimethylsilyloxy)-3-methoxy-1-methylsulfonyl-2-phenylpyrrolidine (29):

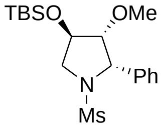

To a solution of amino alcohol **28** (86 mg, 0.26 mmol) in CH_2Cl_2 (4 mL), triethylamine (147 µL, 105 mg, 1.04 mmol) followed by methanesulfonyl chloride (41 µL, 61 mg, 0.53 mmol) were added at 0 °C. After stirring at room temperature overnight, H_2O (8

mL) was added. The two layers were separated, and the aqueous layer was extracted with a few portions of CH_2Cl_2, the combined extracts were dried with $MgSO_4$, and the solvents were removed in vacuo. The crude mixture was purified by column chromatography (silica gel, hexane/EtOAc, 4:1) to furnish compound **29** as a colourless oil (56 mg, 55 %).

$[\alpha]_D^{22}$ = +88.1 (*c* 1.19, $CHCl_3$); ^{1}H NMR ($CDCl_3$, 500 MHz): δ = 0.14, 0.15 (2 s, 3H each, $SiMe_2$), 0.93 (s, 9H, Si^{t}Bu), 2.75 (s, 3H, NMs), 2.96 (s, 3H, OMe), 3.63 (dd_{br}, *J* ≈ 1.1, 11.8 Hz, 1H, 5-H), 3.63 (dd, *J* = 2.4, 5.0 Hz, 1H, 3-H), 3.78 (dd, *J* = 3.5, 11.8 Hz, 1H, 5-H), 4.26–4.27 (m, 1H, 4-H), 4.90 (d, *J* = 5.0 Hz, 1H, 2-H), 7.25–7.39 (m, 5H, Ph) ppm; ^{13}C NMR ($CDCl_3$, 126 MHz): δ = –4.8* (q, $SiMe_2$), 18.1, 25.7 (s, q, Si^{t}Bu), 36.9 (q, NMs), 55.3 (t, C-5), 58.9 (q, OMe), 65.7 (d, C-2), 73.8 (d, C-4), 88.8 (d, C-3), 127.6, 128.0, 128.2, 136.7 (3 d, s, Ph) ppm, *double intensity; IR (ATR): $\tilde{\nu}$ = 3065–2830 (=C-H, C-H), 1345 (S=O), 1160, 1120, 1020 (C-O) cm^{-1}; ESI-TOF (*m*/*z*): calcd. for $C_{18}H_{31}NNaO_4SSi$ $[M + Na]^+$: 408.1635; found: 408.1611.

(2*S*,3*R*,4*R*)-4-(*tert*-Butyldimethylsilyloxy)-3-methoxy-2-phenylpyrrolidine (30):

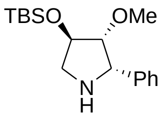

To a solution of freshly prepared, crude amino alcohol **28** (350 mg, 1.07 mmol) in CH_2Cl_2 (20 mL), were added triethylamine (166 µL, 121 mg, 1.21 mmol), CBr_4 (414 mg, 1.25 mmol), and PPh_3 (328 mg, 1.25 mmol) at 0 °C. The resulting mixture was stirred overnight at room temperature and quenched with aqueous NaOH soln. (1 N, ca. 20 mL). The layers were separated, the aqueous layer was extracted with a few portions of Et_2O, and the organics were combined, dried over $MgSO_4$ and filtered, and the solvents were removed under reduced pressure. The crude mixture

was filtered through a short silica gel pad to give enriched **30** (210 mg), with the triphenylphosphine oxide as the main inpurity. Additional column chromatography (silica gel, diethylether) furnished **30** (110 mg, 33%) as a colourless oil.

$[\alpha]_D^{22}$ = +29.7 (*c* 1.11, $CHCl_3$); ^1H NMR ($CDCl_3$, 500 MHz): δ = 0.11, 0.13 (2 s, 3H each, $SiMe_2$), 0.92 (s, 9H, Si^tBu), 2.83 (dd, *J* = 3.0, 11.6 Hz, 1H, 5-H), 3.09 (s, 3H, OMe), 3.47 (dd, *J* = 5.5, 11.6 Hz, 1H, 5-H), 3.58 (dd, *J* = 1.2, 4.1 Hz, 1H, 3-H), 4.31 (ddd, *J* = 1.2, 3.0, 5.5 Hz, 1H, 4-H), 4.34 (d, *J* = 4.1 Hz, 1H, 2-H), 7.22–7.26, 7.30–7.34, 7.38–7.41 (3 m, 5H, Ph) ppm; ^{13}C NMR ($CDCl_3$, 126 MHz): δ = –4.73, –4.68 (2 q, $SiMe_2$), 18.0, 25.8 (s, q, Si^tBu), 54.4 (t, C-5), 57.9 (q, OMe), 64.3 (d, C-2), 76.4 (d, C-4), 89.7 (d, C-3), 126.8, 127.8, 127.9, 138.9 (3 d, s, Ph) ppm; IR (ATR): $\tilde{\nu}$ = 3315 (N-H), 3090–2825 (=C-H, C-H), 1105 (C-O) cm^{-1}; ESI-TOF (*m*/*z*): calcd. for $C_{17}H_{30}NO_2Si$ $[M + H]^+$: 308.2040; found: 308.2055.

Synthesis of pyrrolidine derivative 30 by demesylation of 29

To a solution of mesylated pyrrolidine **29** (50 mg, 0.13 mmol) in THF (8 mL), a freshly prepared solution of LDA [by treatment of diisopropylamine (1.0 equiv.) with BuLi (1.0 equiv.) at –78 °C in THF and stirring for 30 min] was added (0.70 mmol added in three portions, ~0.23 mmol each every 4 h) at room temperature and stirred for 16 h. The reaction mixture was quenched with sat. NH_4Cl solution followed by the addition of EtOAc. The two layers were separated, and the aqueous layer was extracted with CH_2Cl_2. The combined organics were dried over $MgSO_4$, filtered and concentrated. Compound **30** (5 mg, 13%) was isolated by chromatography (silica gel, dichloromethane/methanol 30:1) as a light orange oil.

Attempted cyclisation of 28 with *p*TsCl

To a solution of freshly prepared unpurified amino alcohol **28** (501 mg, 1.54 mmol) in dry CH_2Cl_2 (35 mL), triethylamine (1.24 mL, 893 mg, 8.82 mmol) followed by pTsCl (656 mg, 3.44 mmol) was slowly added at 0 °C, and stirred at room temperature overnight. Then the reaction was quenched with H_2O, the resulting solution was extracted with a few portions of CH_2Cl_2, the combined organics were dried over Na_2SO_4 and filtered, and the solvents were removed. The crude mixture was chromatographed on silica (hexane/ethyl acetate 3:1 gradient to 1:1) to furnish an inseparable ~1:1 mixture of *N*,*O*-tosylated compounds (136 mg, 14%, first eluted fraction, see Results and Discussion part) and ~4:1 mixture of tosylamides (301 mg, 41%). The latter fraction was additionally purified (silica gel, hexane/ethyl acetate 3:1) to yield analytically pure **31** (177 mg, 24%) as a colourless solid.

(2*R*,3*R*,4*S*)-2-(*tert*-Butyldimethylsilyl)-3-methoxy-4-phenyl-4-(*p*-toluenesulfonylamino)butan-1-ol (31):

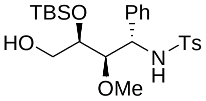

mp 124-126 °C; $[\alpha]_D^{22}$ = +22.5 (*c* 0.98, $CHCl_3$); ^{1}H NMR ($CDCl_3$, 500 MHz): δ = 0.07, 0.08 (2 s, 3H each, $SiMe_2$), 0.89 (s, 9H, Si^{t}Bu), 2.00 (dd_{br}, *J* ≈ 3.1, 8.8 Hz, 1H, OH), 2.29 (s, 3H, Me), 3.07 (s, 3H, OMe), 3.36 (dd, *J* = 1.5, 7.6 Hz, 1H, 3-H), 3.65 (dt_{br}, *J* ≈ 2.5, 12.0 Hz, 1H, 1-H), 3.77 (ddd_{br}, *J* = 3.3, 8.8, 12.0 Hz, 1H, 1-H), 3.81 (dt, *J* ≈ 3.3, 7.6 Hz, 1H, 2-H), 4.63 (dd_{br}, *J* ≈ 1.5, 9.5 Hz, 1H, 4-H), 5.55 (d, *J* = 9.5 Hz, 1H, NH), 7.01 (d, *J* = 8.2 Hz, 2H, Ts), 7.03–7.11 (m, 5H, Ph), 7.46 (d, *J* = 8.2 Hz, 2H, Ts) ppm; ^{13}C NMR ($CDCl_3$, 126 MHz): δ = –4.8, –4.6 (2 q, $SiMe_2$), 18.0 (s, Si^{t}Bu), 21.3 (q, Me), 25.8 (q, Si^{t}Bu), 56.6 (d, C-4), 61.3 (q, OMe), 63.7 (t, C-1), 73.3 (d, C-2), 86.1 (d, C-3),

126.5, 126.9, 127.0 128.1, 129.1, 137.7, 139.4, 142.9 (5 d, 3 s, Ph, Ts) ppm; IR (ATR): $\tilde{\nu}$ = 3580-3175 (O-H, N-H), 3035–2825 (=C-H, C-H), 1335 (S=O), 1155, 1090, 1050 (C-O) cm^{-1}; ESI-TOF (*m*/*z*): calcd. for $C_{24}H_{37}NNaO_5SSi$ $[M + Na]^+$: 502.2054; found: 502.2041. Anal. calcd. for $C_{24}H_{37}NO_5SSi$ (479.7): C 60.09, H 7.77, N 2.92; found: C 60.09, H 7.79, N 2.93.

(3*R*,4*R*,5*S*)-4-Methoxy-5-phenylpyrrolidin-3-ol (32):

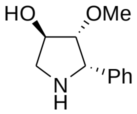

Pyrrolidine **30** (51 mg, 0.16 mmol) was dissolved in 1 N HCl in MeOH (8 mL) and stirred at room temperature for three days. The reaction mixture was quenched with excess solid $NaHCO_3$, the inorganics were filtered off and the solvents removed to dryness. The crude mixture was chromatographed on silica gel (dichloromethane/methanol 9:1) to give **32** (23 mg, 72%) as colourless crystals.

mp 192-195 °C; $[\alpha]_D^{22}$ = +48.3 (*c* 0.88, CH_3OH); ^1H NMR (CD_3OD, 500 MHz): δ = 3.29 (d_{br}, $J \approx 12.5$ Hz, 1H, 2-H), 3.32 (s, 3H, OMe), 3.75 (dd, J = 4.8, 12.5 Hz, 1H, 2-H), 3.93 (dd_{br}, $J \approx 0.9$, 3.2 Hz, 1H, 4-H), 4.60 (dt_{br}, $J \approx 0.9$, 4.8 Hz, 1H, 3-H), 4.85 (d, J = 3.2 Hz, 1H, 5-H), 7.41–7.48, 7.51–7.54 (2 m, 5H, Ph) ppm; ^{13}C NMR ($CDCl_3$, 126 MHz): δ = 53.0 (t, C-2), 58.5 (q, OMe), 65.4 (d, C-5), 72.5 (d, C-3), 87.4 (d, C-4), 129.7, 129.9, 130.3, 132.3 (3 d, s, Ph) ppm; IR (neat): $\tilde{\nu}$ = 3400–3250 (O-H, N-H), 3100–2805 (=C-H, C-H), 1100 (C-O) cm^{-1}; ESI-TOF (*m*/*z*): calcd. for $C_{11}H_{16}NO_2$ $[M + H]^+$: 194.1181; found: 194.1153.

References

1. Pérez, M.; Canoa, P.; Gómez, G.; Teijeira, M.; Fall, Y. *Synthesis* **2005,** 411–414. doi:10.1055/s-2004-834946
2. Helms, M.; Schade, W.; Pulz, R.; Watanabe, T.; Al-Harrasi, A.; Fišera, L.; Hlobilová, I.; Zahn, G.; Reissig, H.-U. *Eur. J. Org. Chem.* **2005,** 1003–1019. doi:10.1002/ejoc.200400627
3. Krijnen, E. S.; Zuilhof, H.; Lodder, G. *J. Org. Chem.* **1994,** *59,* 8139–8150. doi:10.1021/jo00105a035
4. Kucerovy, A.; Neuenschwander, K.; Meinreb, S. M. *Synthetic Commun.* **1983,** *13,* 875–879. doi:10.1080/00397918308059540
