# Supplementary material for: Carbohydrate-auxiliary assisted preparation of enantiopure 1,2-oxazine derivatives and aminopolyols
Source: Beilstein J Org Chem. 2012 Apr 30;8:662–74. doi: 10.3762/bjoc.8.74 (PMC3388852; doi:10.3762/bjoc.8.74)
Supplement: File 2 — 1H NMR and 13C NMR spectra of synthesised compounds. [file Beilstein_J_Org_Chem-08-662-s002.pdf]

Supporting Information File 2

for

Carbohydrate-auxiliary assisted preparation of enantiopure 1,2-oxazine derivatives and aminopolyols

Marcin Jasiński[1,2], Dieter Lentz[1,§] and Hans-Ulrich Reissig*[,1]

Address: [1]Institut für Chemie und Biochemie, Freie Universität Berlin, Takustr. 3, D-14195 Berlin, Germany and [2]Department of Organic and Applied Chemistry, University of Łódź, Tamka 12, PL-91-403 Poland

Email: Hans-Ulrich Reissig* - hreissig@chemie.fu-berlin.de

* Corresponding author

§ Responsible for X-ray crystal structure determination

^{1}H NMR and ^{13}C NMR spectra of synthesised compounds

Compound (3*S*)-3a:

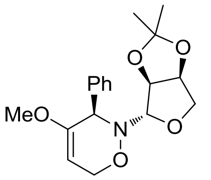

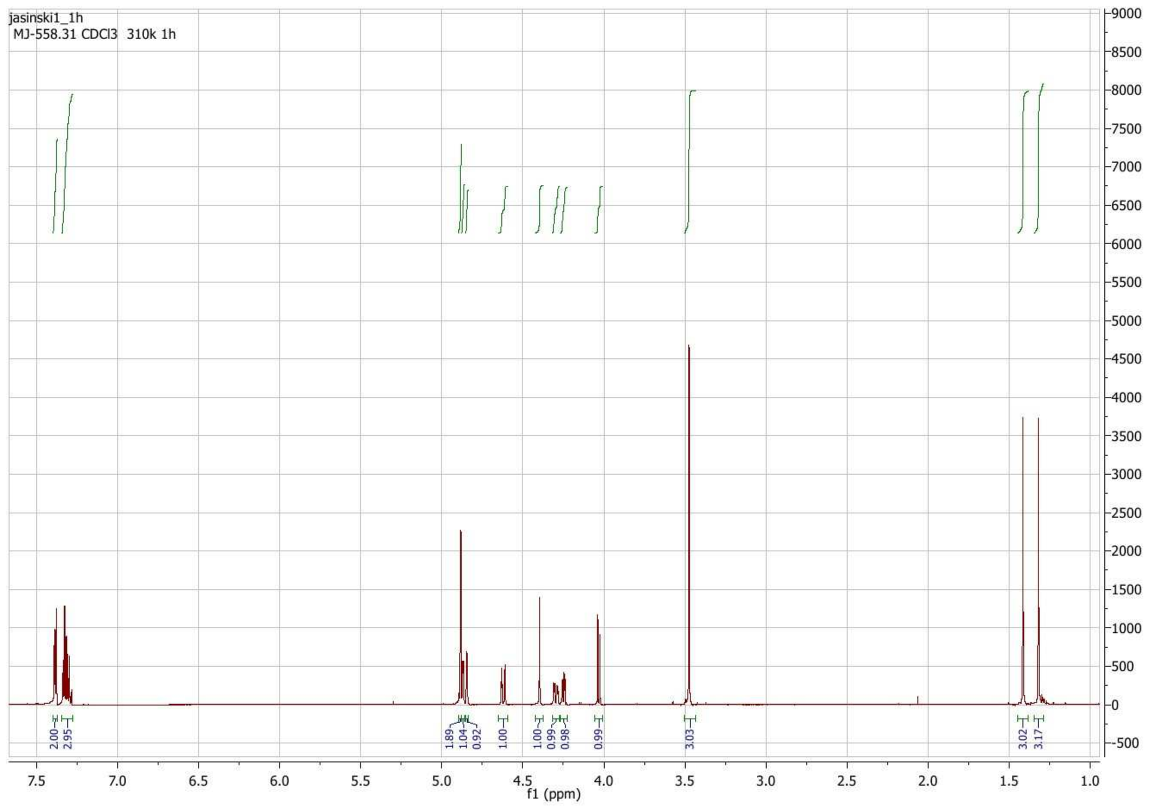

jasinski1_1h
MJ-558.31 CDCl3 310k 1h
f1 (ppm)
2.00
2.95
1.89
1.04
0.92
1.00
1.00
0.99
0.98
0.99
3.03
3.02
3.17

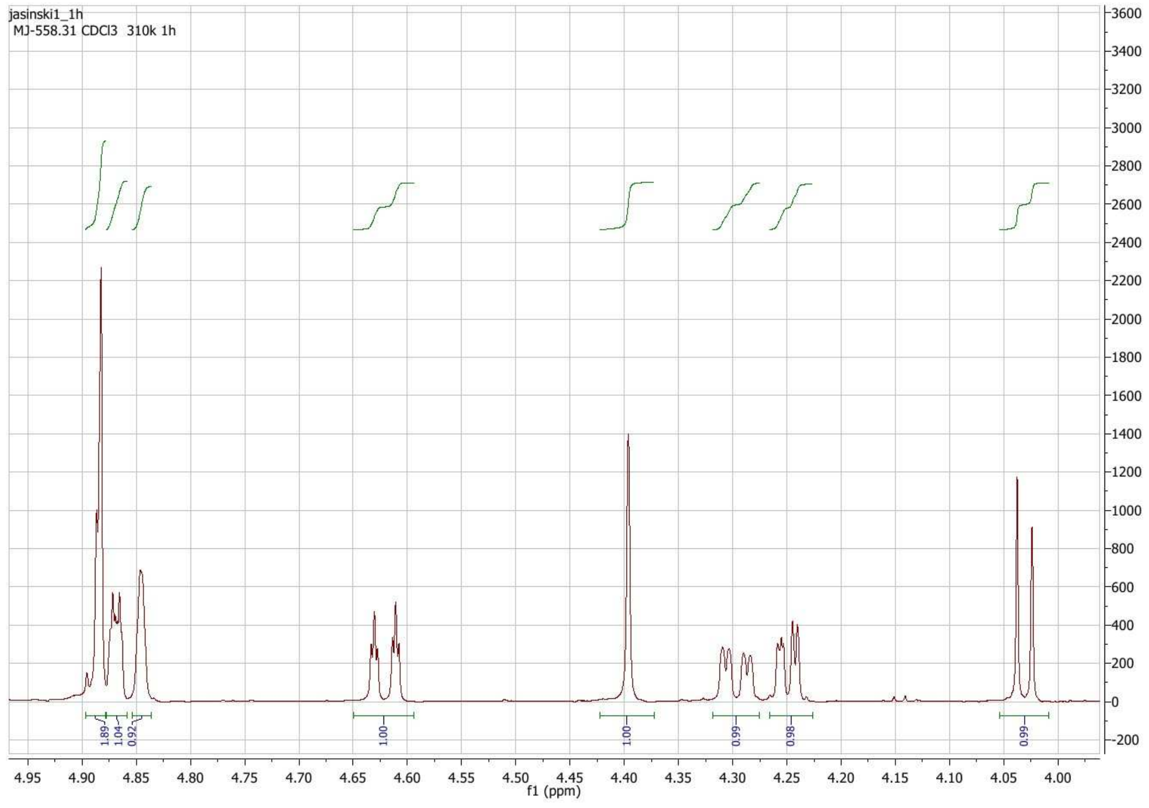

jasinski1_1h
MJ-558.31 CDCl3 310k 1h
f1 (ppm)
1.89
1.04
0.92
1.00
1.00
0.99
0.98
0.99

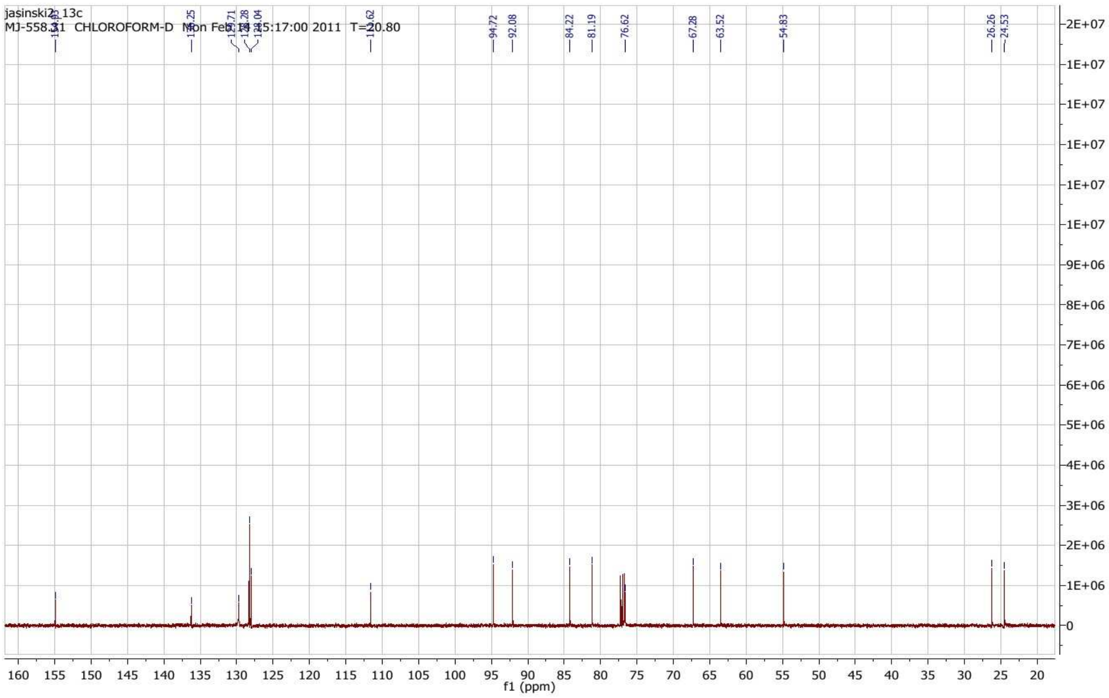

Compound (3*R*)-3a:

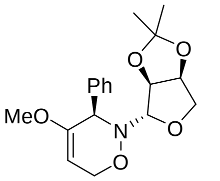

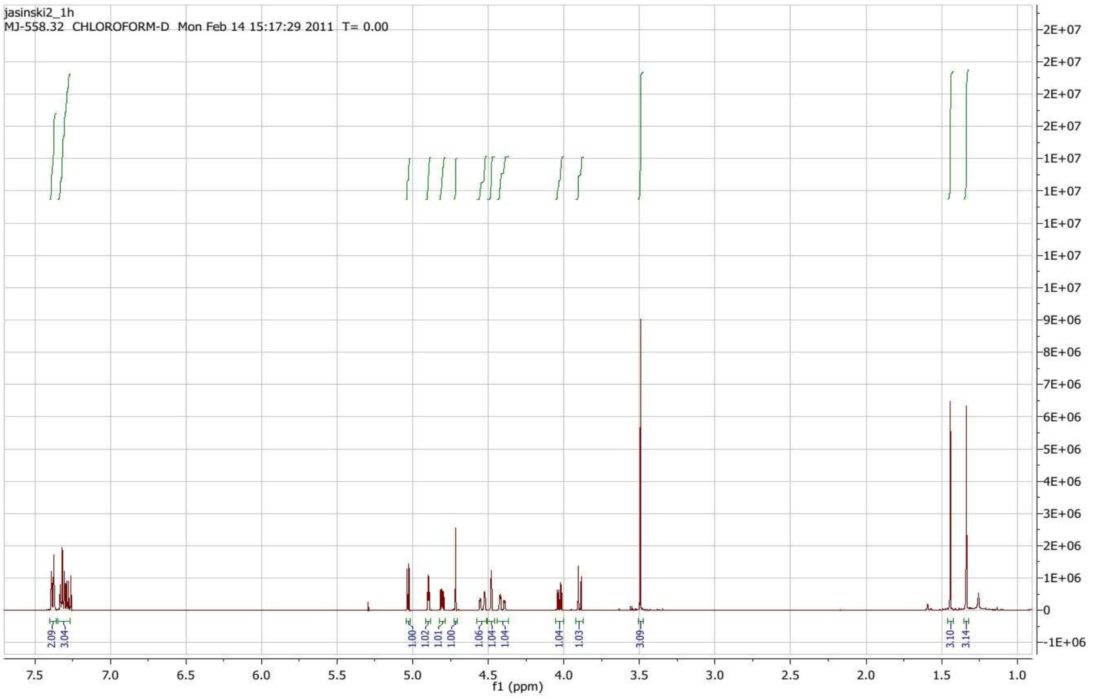

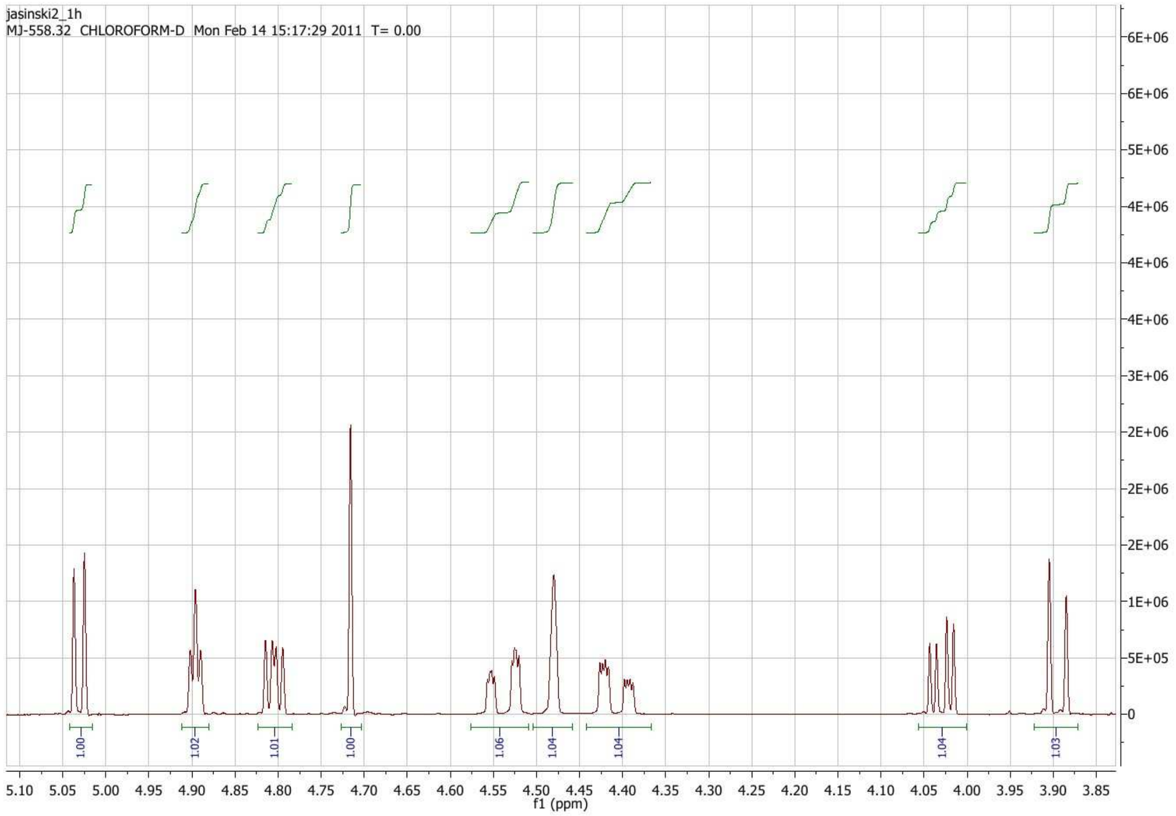
jasinski2_1h
MJ-558.32 CHLOROFORM-D Mon Feb 14 15:17:29 2011 T= 0.00
1.00
1.02
1.01
1.00
1.06
1.04
1.04
1.04
1.03
f1 (ppm)

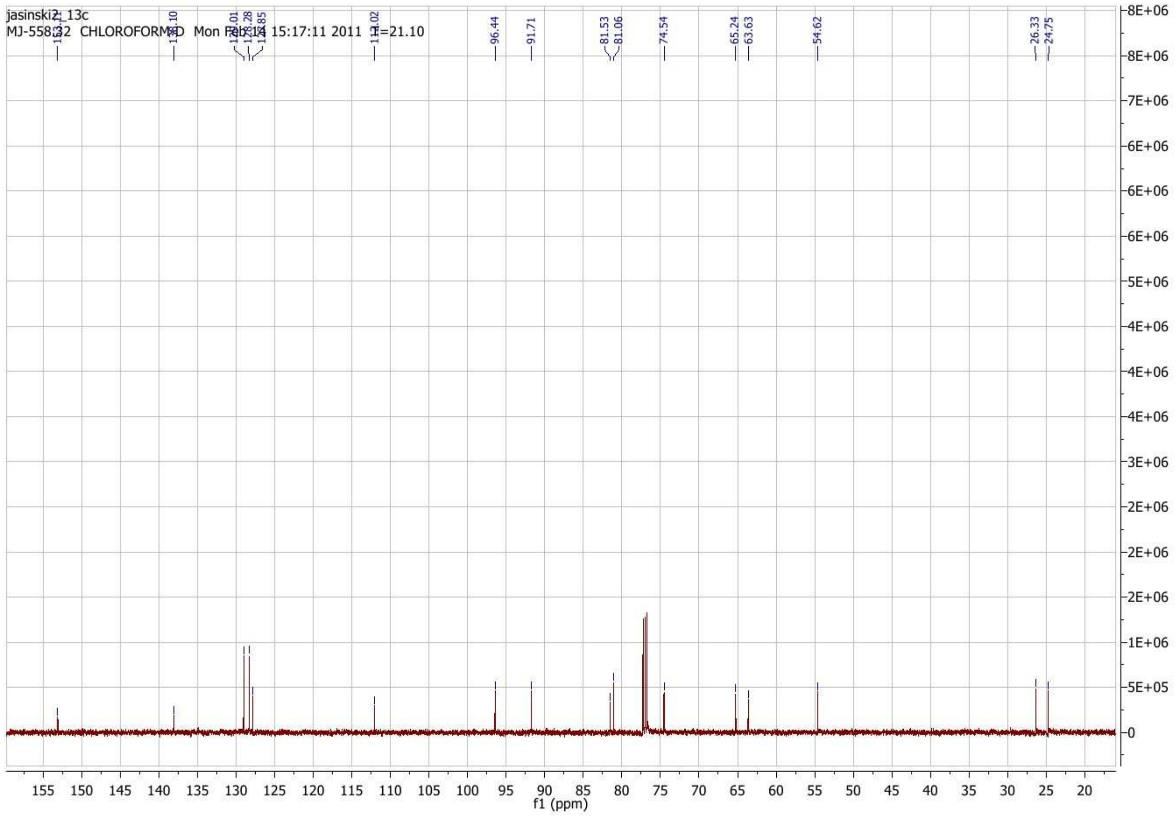
jasinski2_13c
MJ-558.32 CHLOROFORM-D Mon Feb 14 15:17:11 2011 T=21.10
96.44
91.71
81.53
81.06
74.54
65.24
63.63
54.62
26.33
24.75
f1 (ppm)

Compound (3*S*)-3b:

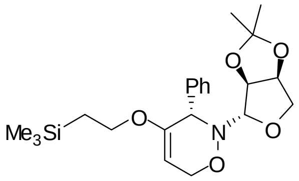

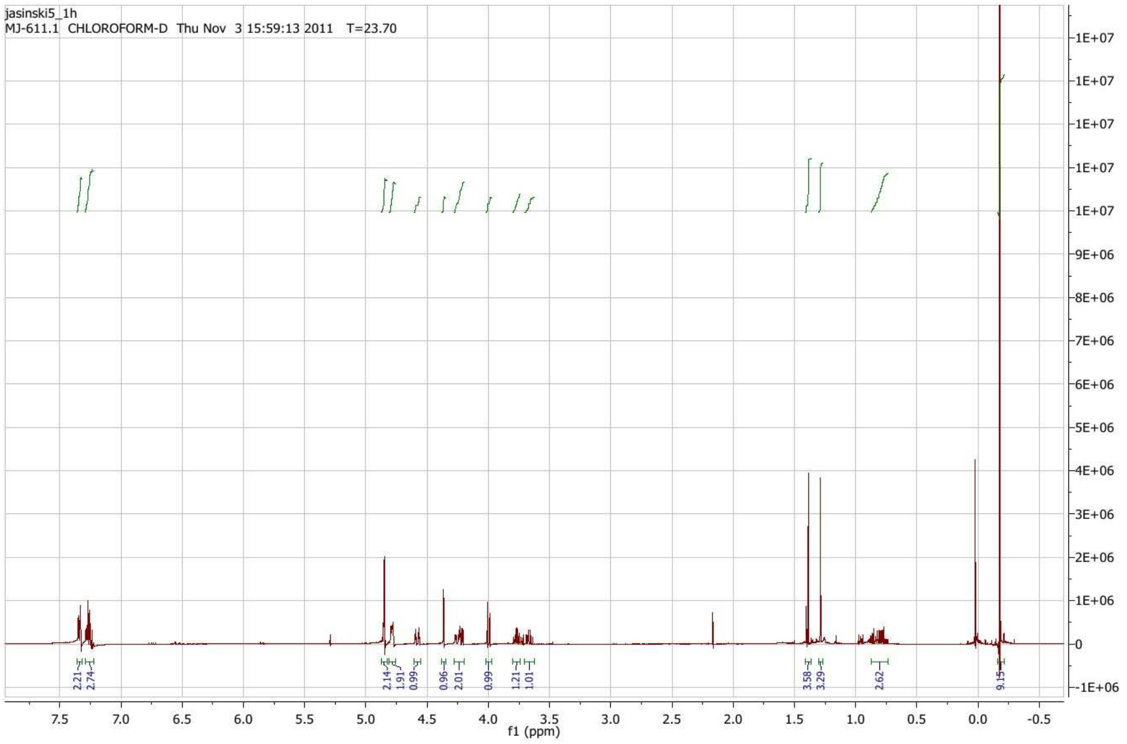

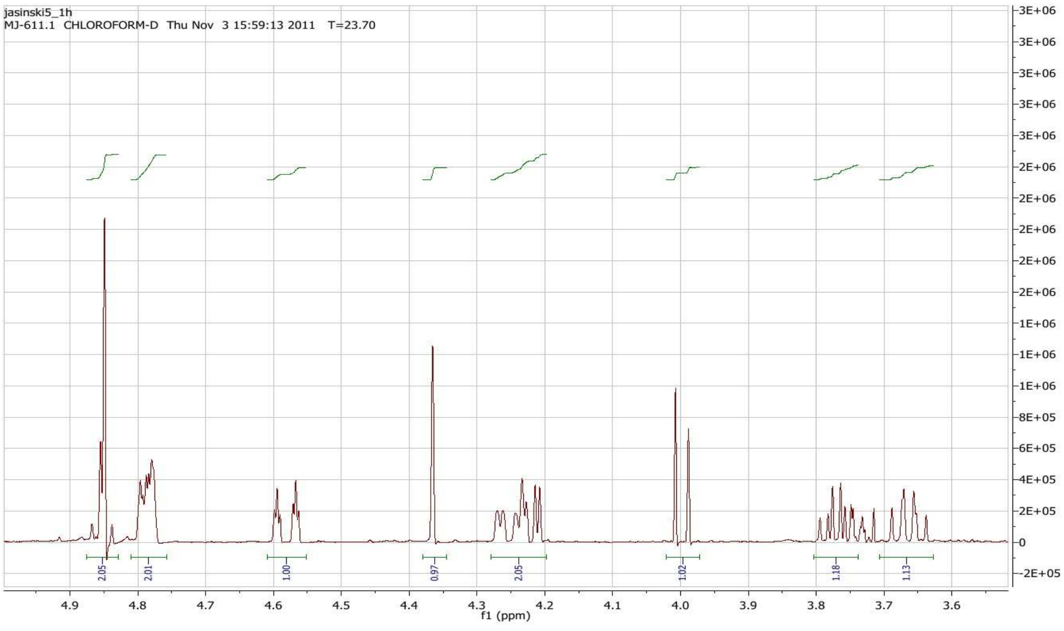

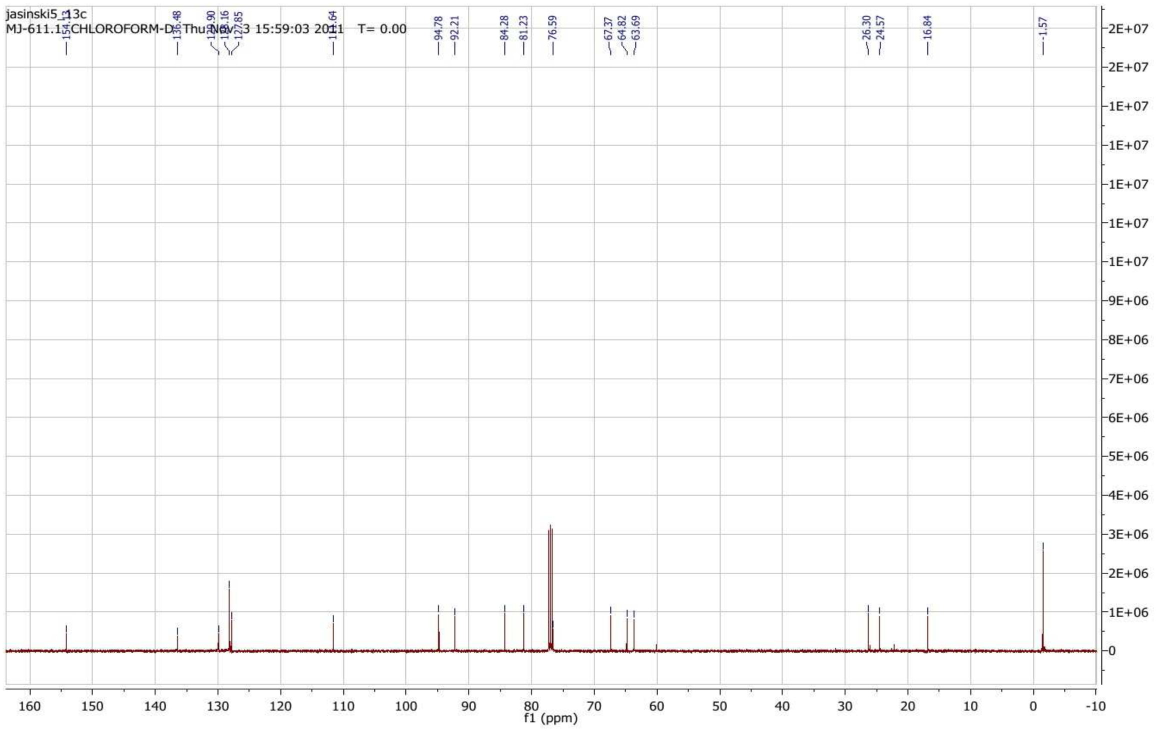

Compound (3*R*)-3b:

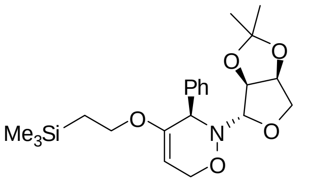

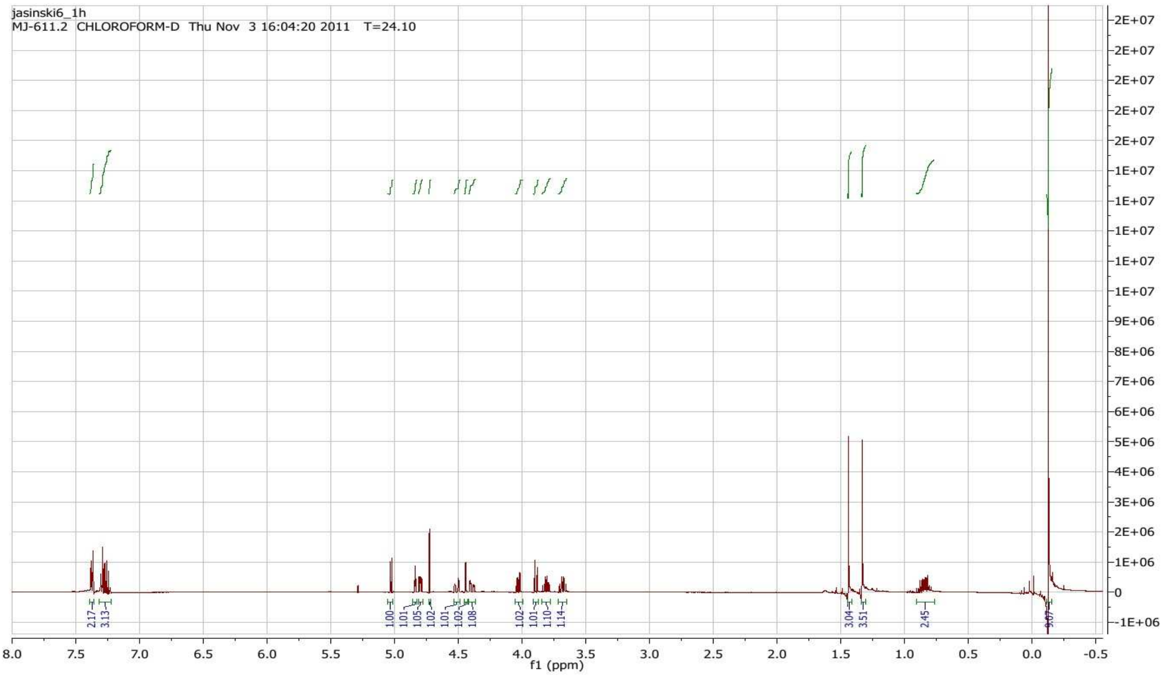

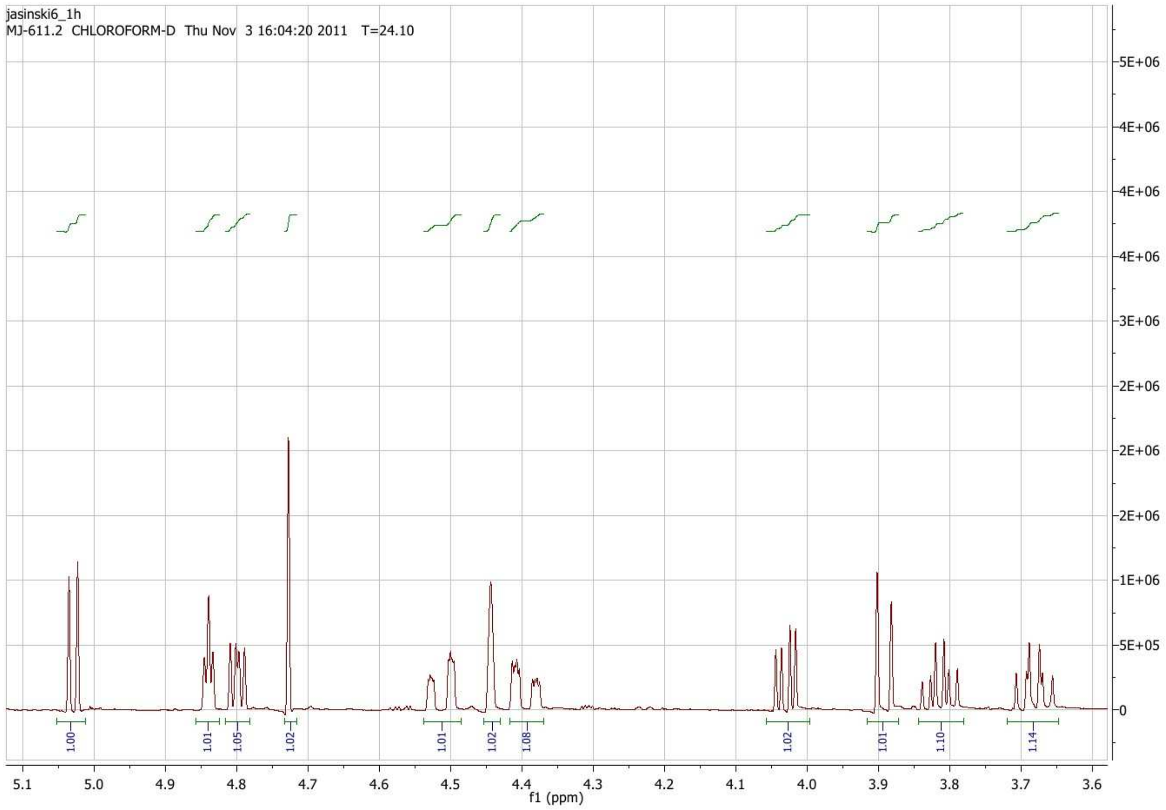
jasinski6_1h
MJ-611.2 CHLOROFORM-D Thu Nov 3 16:04:20 2011 T=24.10
f1 (ppm)

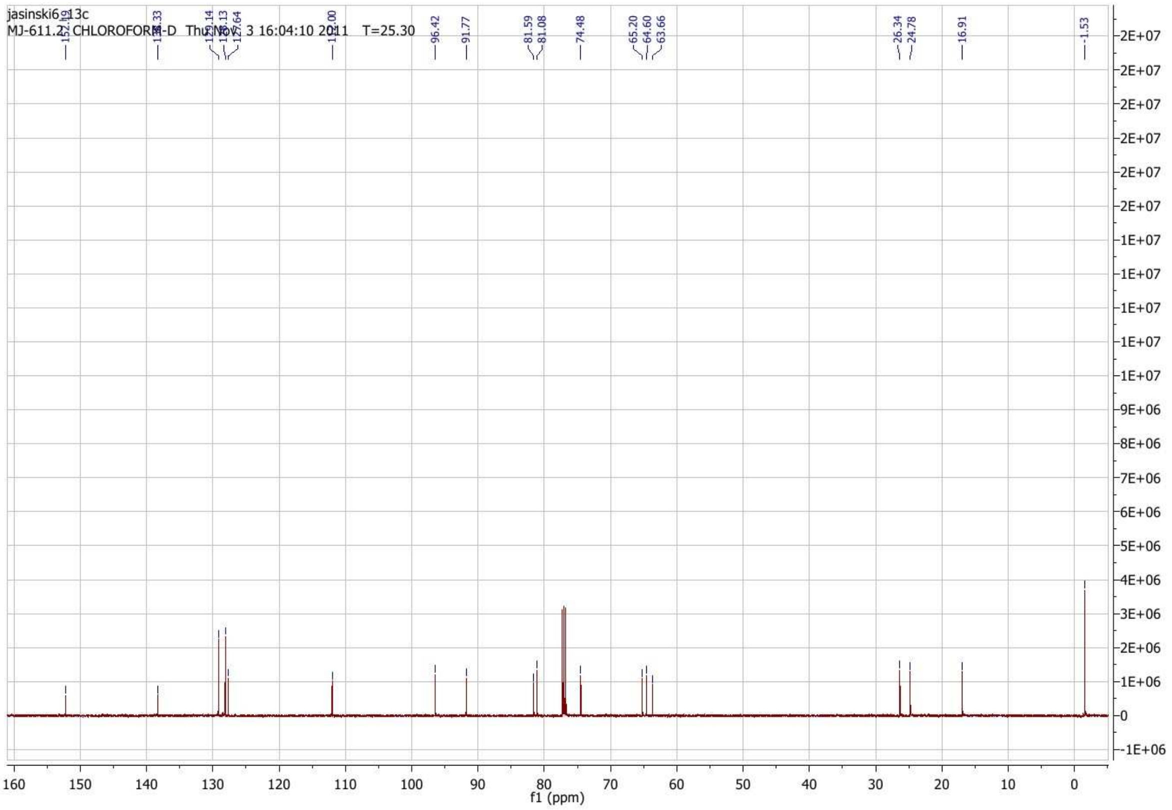
jasinski6_13c
MJ-611.2 CHLOROFORM-D Thu Nov 3 16:04:10 2011 T=25.30
96.42
91.77
81.59
81.08
74.48
65.20
64.60
63.66
26.34
24.78
16.91
-1.53
f1 (ppm)

Compound (3*S*)-3c:

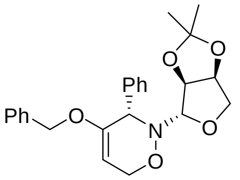

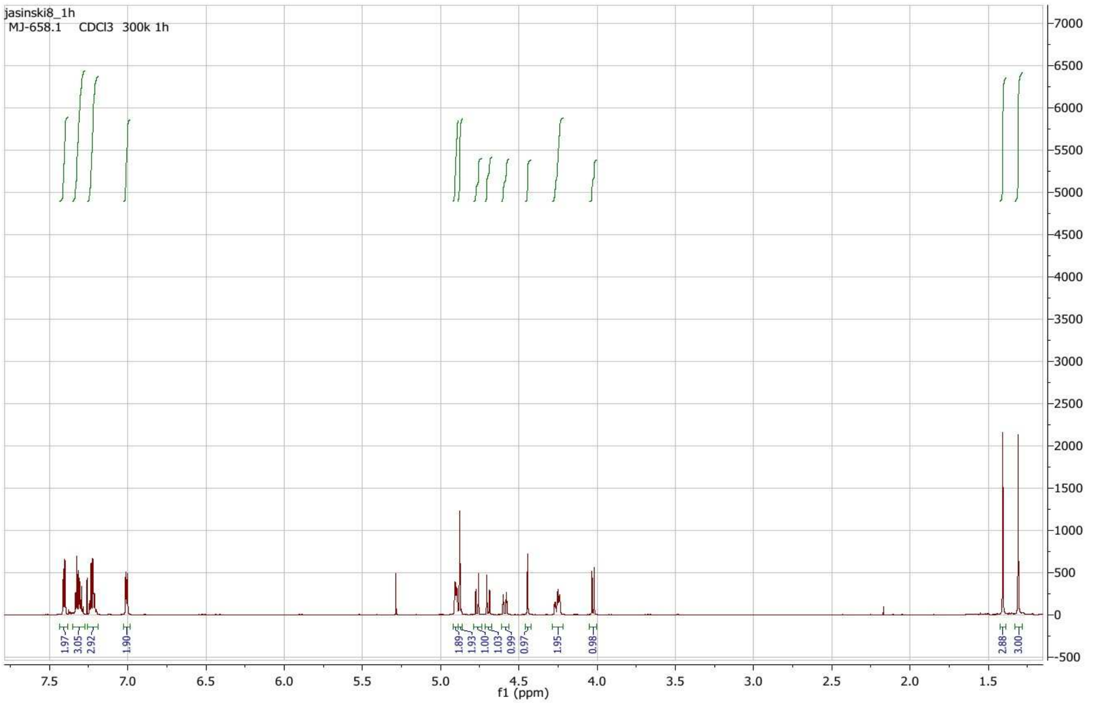

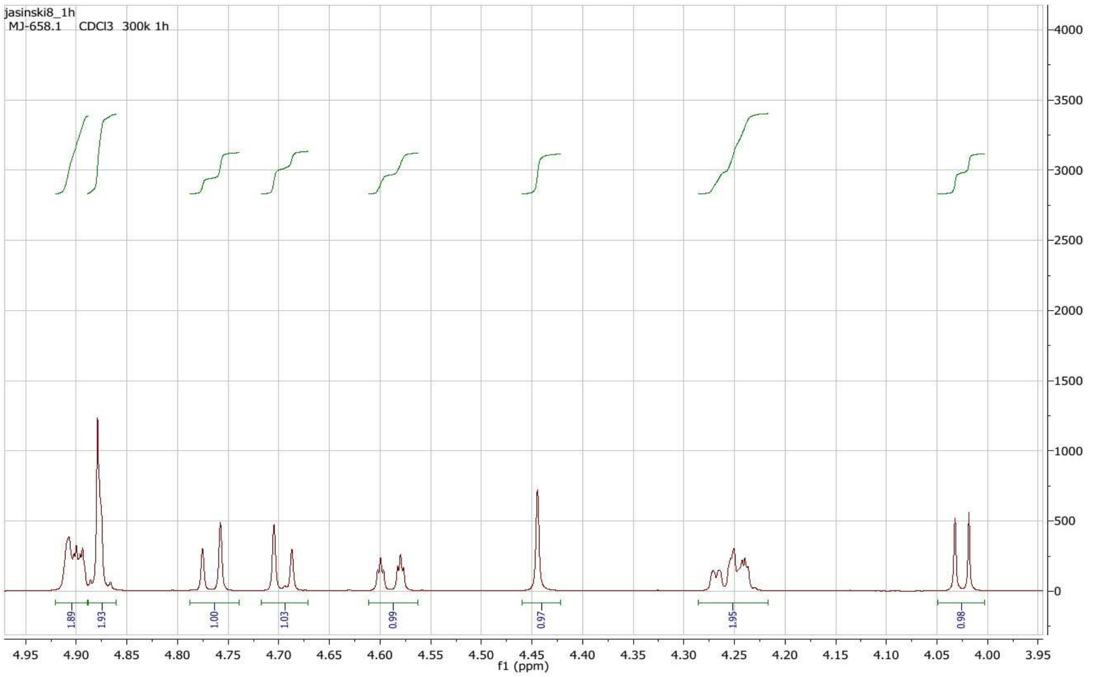

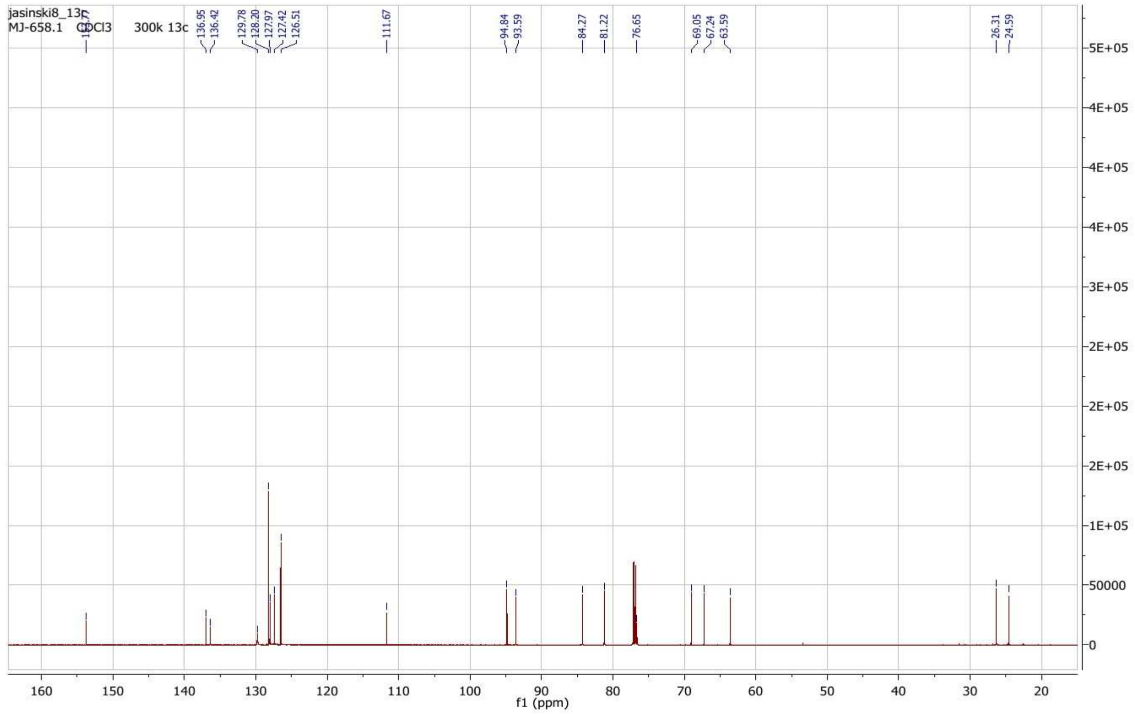

Compound (3*R*)-3c:

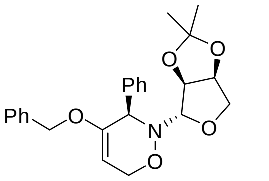

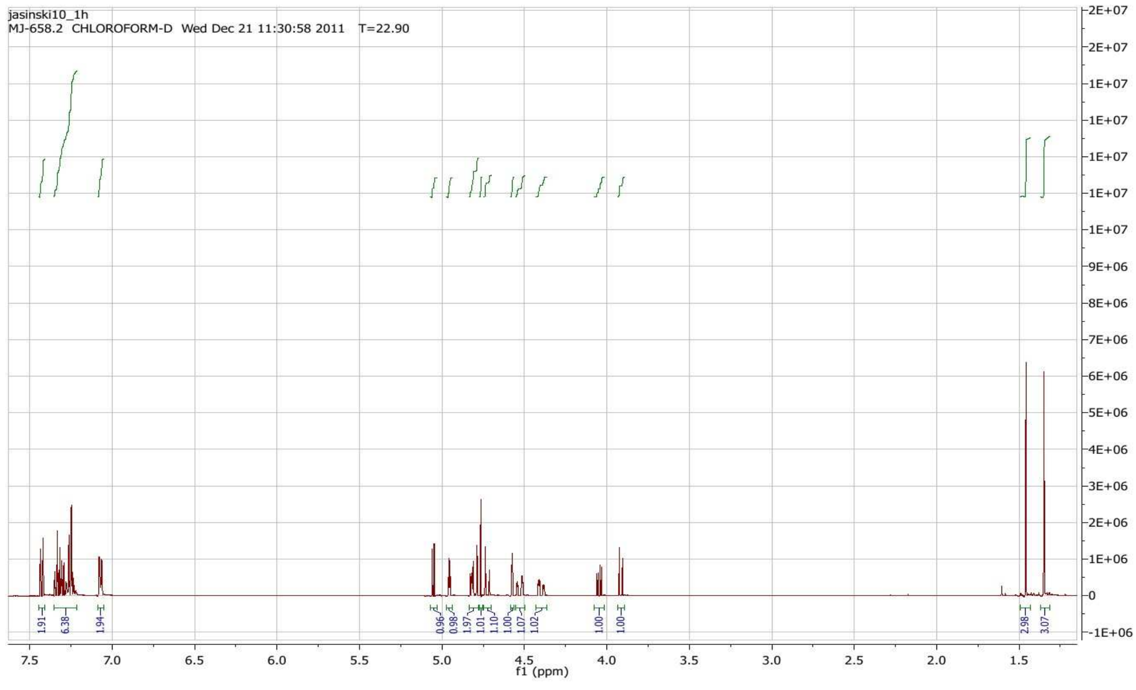

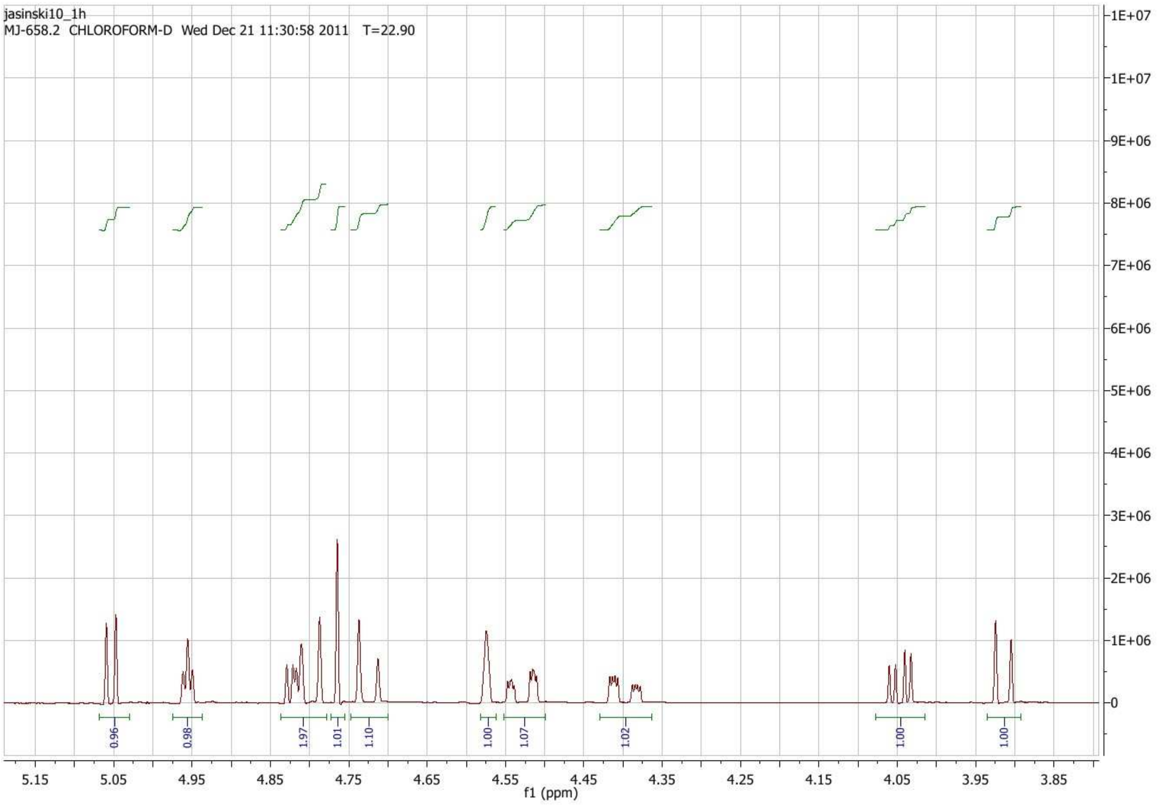
jasinski10_1h
MJ-658.2 CHLOROFORM-D Wed Dec 21 11:30:58 2011 T=22.90
0.96
0.98
1.97
1.01
1.10
1.00
1.07
1.02
1.00
1.00
f1 (ppm)

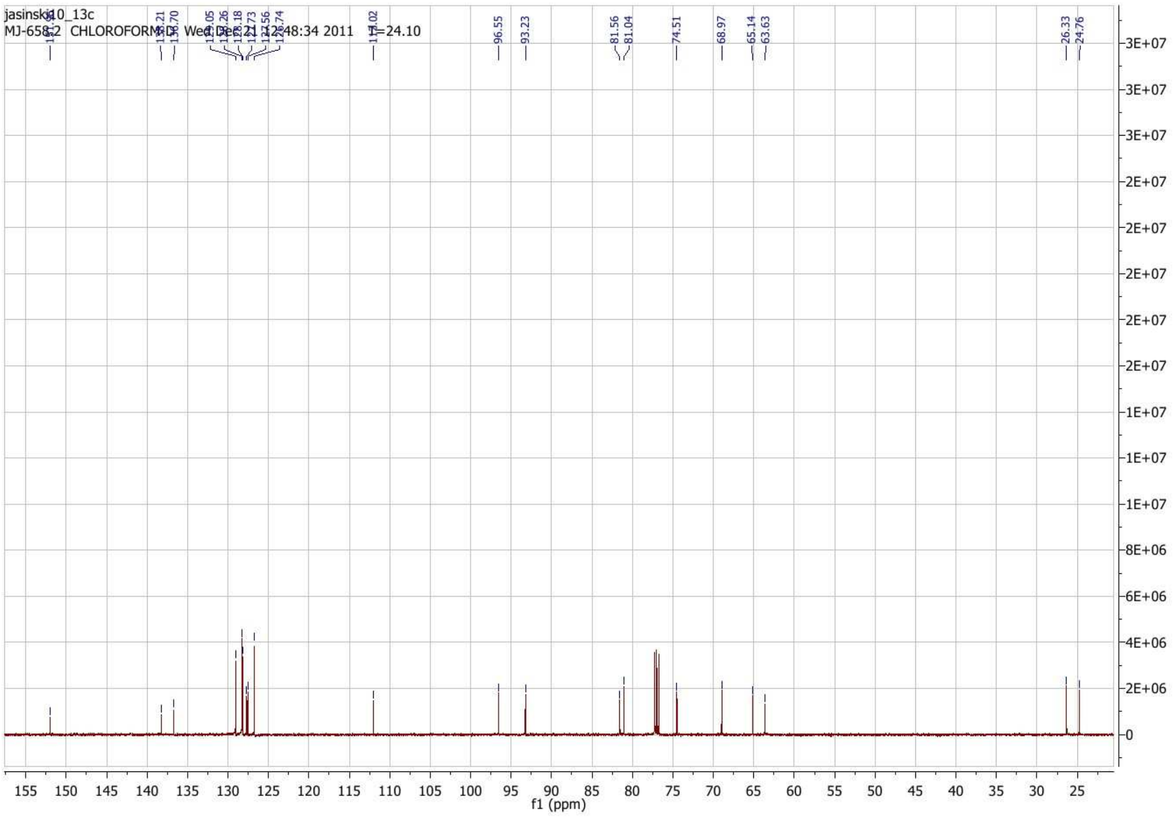
jasinski10_13c
MJ-658.2 CHLOROFORM-D Wed Dec 21 12:48:34 2011 T=24.10
96.55
93.23
81.56
81.04
74.51
68.97
65.14
63.63
26.33
24.76
f1 (ppm)

Compound 5:

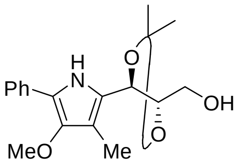
Ph
H
N
O
OH
O
MeO
Me

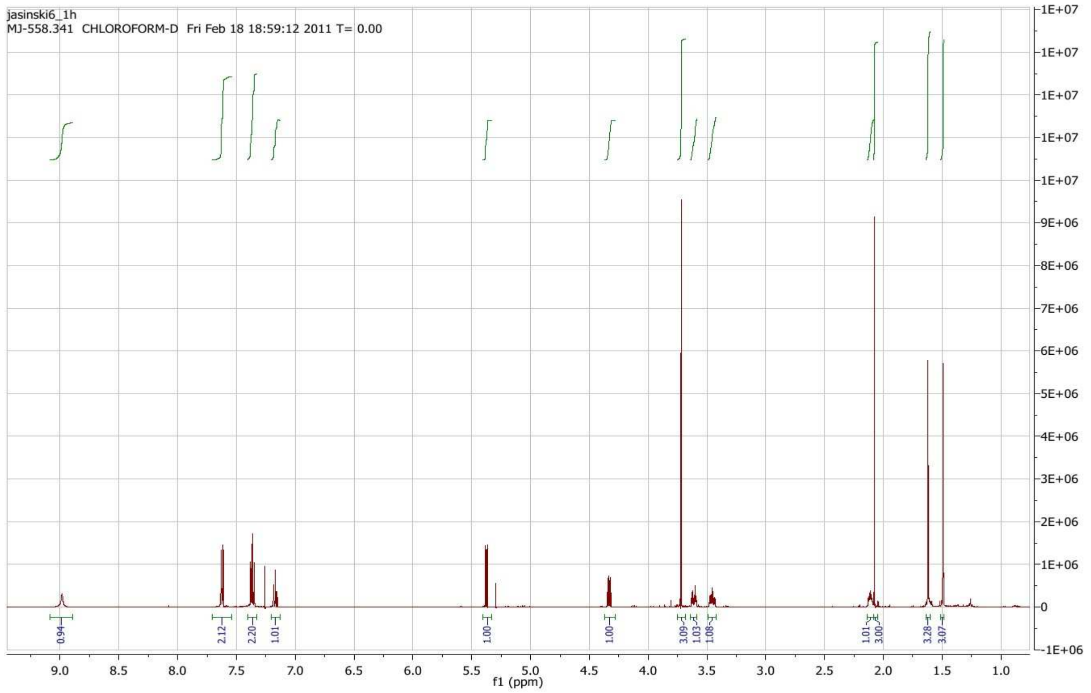
jasinski6_1h
MJ-558.341 CHLOROFORM-D Fri Feb 18 18:59:12 2011 T= 0.00
f1 (ppm)

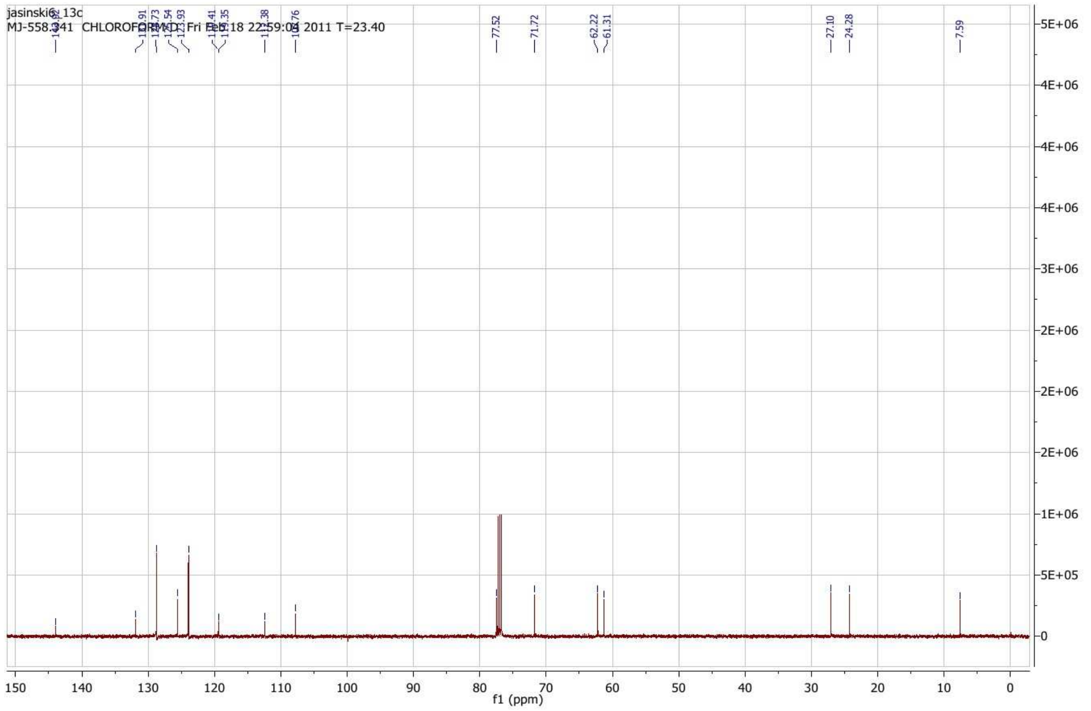
jasinski6_13c
77.52
71.72
62.22
61.31
27.10
24.28
7.59
f1 (ppm)

Compound 6:

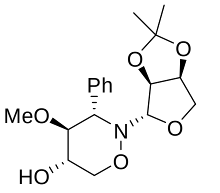
Ph
MeO
N
O
HO
O
O
O

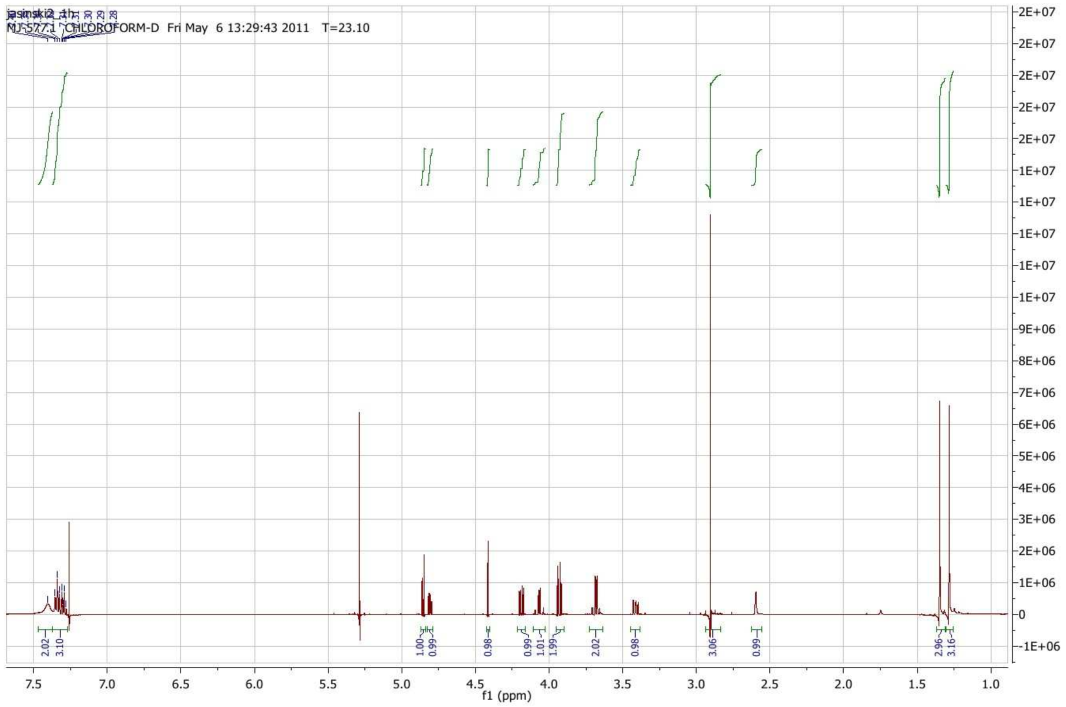
jasinski2_1h
MJ-577.1 CHLOROFORM-D Fri May 6 13:29:43 2011 T=23.10
f1 (ppm)

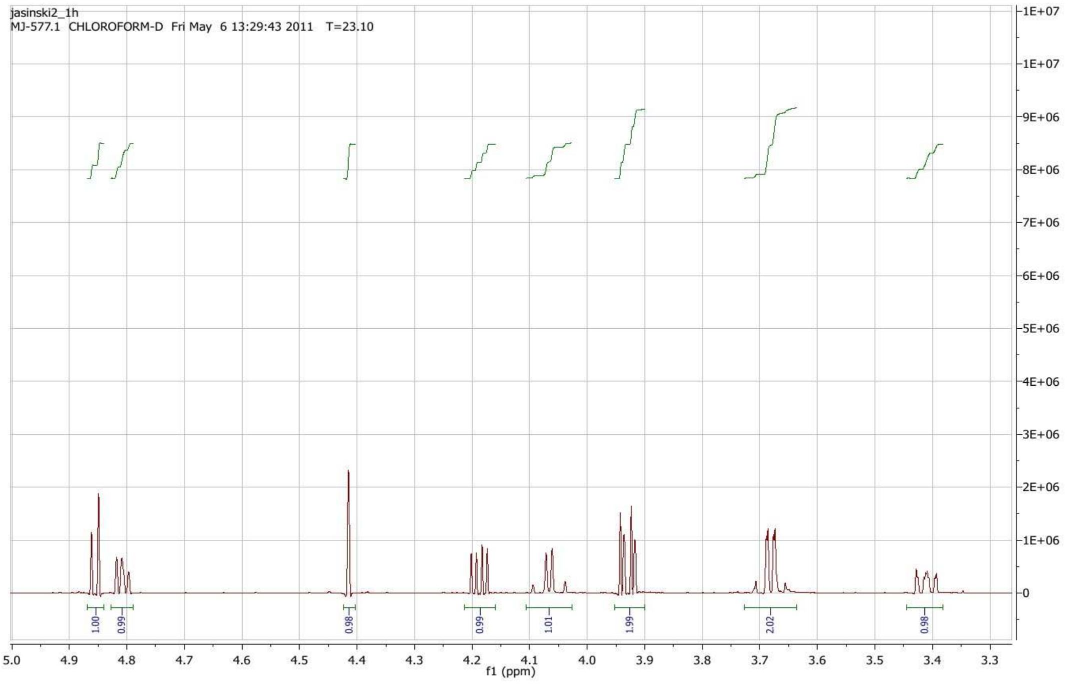
jasinski2_1h
MJ-577.1 CHLOROFORM-D Fri May 6 13:29:43 2011 T=23.10
f1 (ppm)

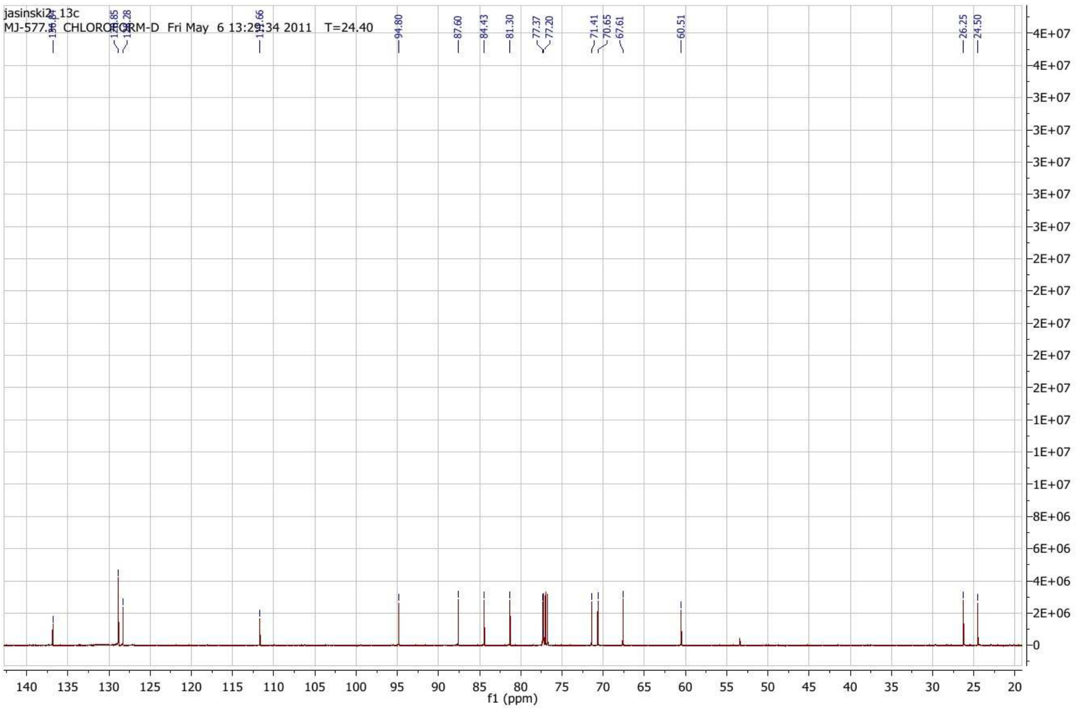
jasinski2_13c
MJ-577.1 CHLOROFORM-D Fri May 6 13:29:34 2011 T=24.40
f1 (ppm)

Compound 7:

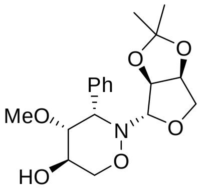
Ph
MeO
HO
N
O
O
O
O

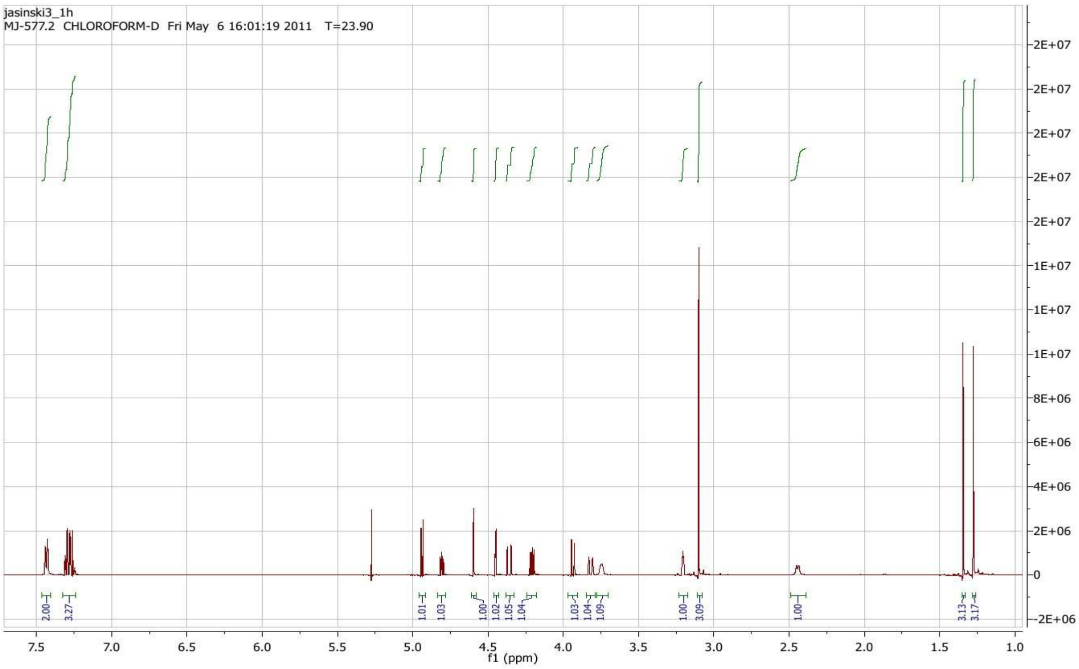
jasinski3_1h
MJ-577.2 CHLOROFORM-D Fri May 6 16:01:19 2011 T=23.90
f1 (ppm)

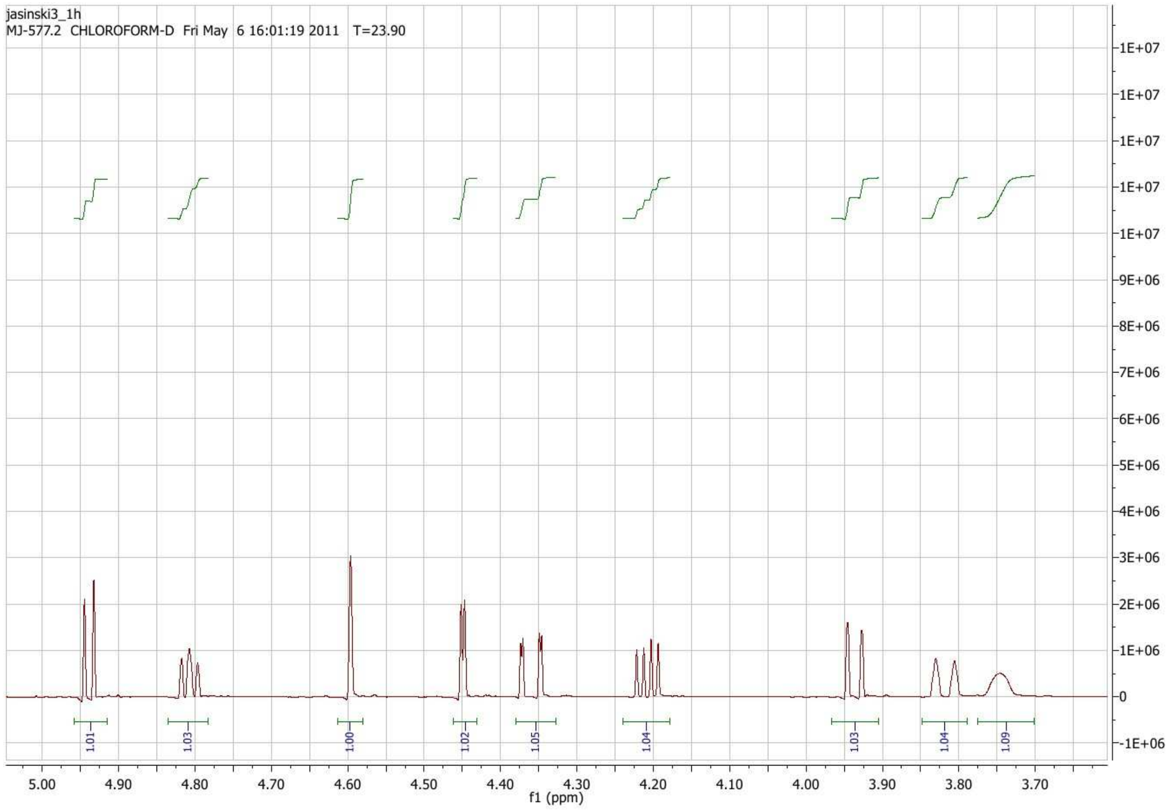
jasinski3_1h
MJ-577.2 CHLOROFORM-D Fri May 6 16:01:19 2011 T=23.90
1.01
1.03
1.00
1.02
1.05
1.04
1.03
1.04
1.09
5.00 4.90 4.80 4.70 4.60 4.50 4.40 4.30 4.20 4.10 4.00 3.90 3.80 3.70
f1 (ppm)

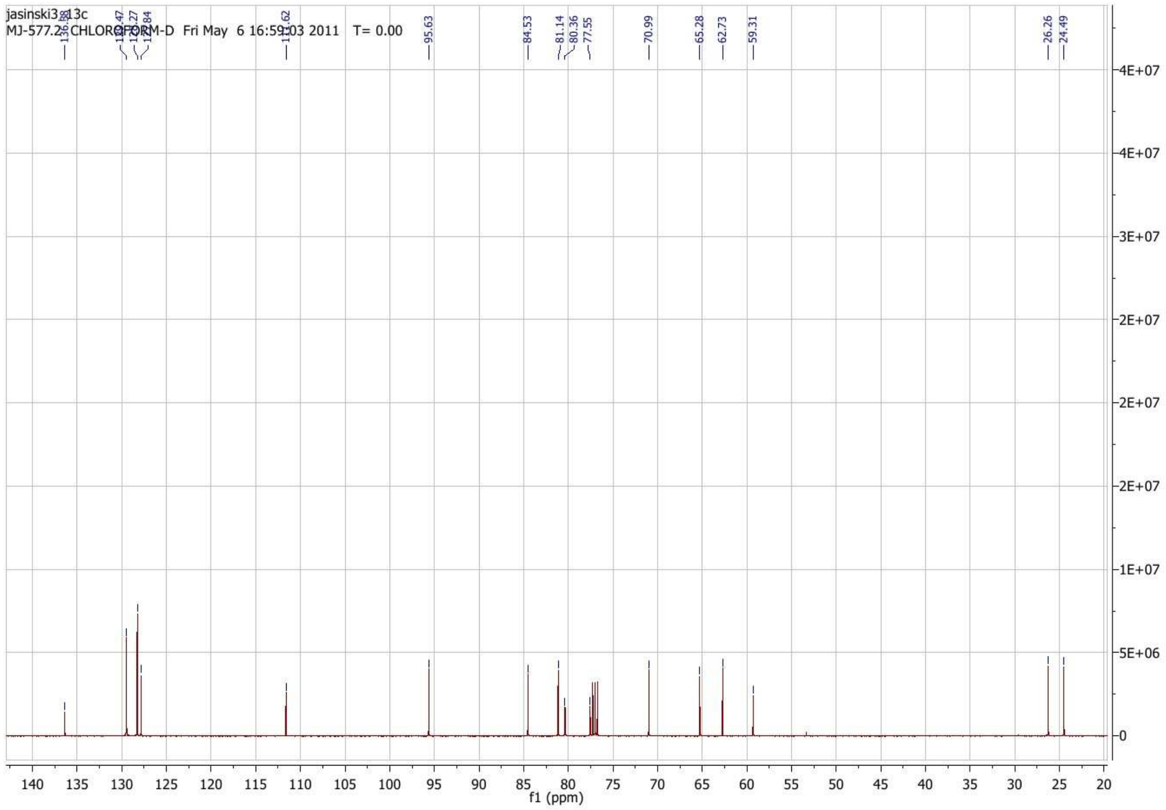
jasinski3_13c
MJ-577.2 CHLOROFORM-D Fri May 6 16:59:03 2011 T= 0.00
95.63
84.53
81.14
80.36
77.55
70.99
65.28
62.73
59.31
26.26
24.49
140 135 130 125 120 115 110 105 100 95 90 85 80 75 70 65 60 55 50 45 40 35 30 25 20
f1 (ppm)

Compound 8:

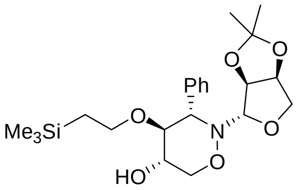

Ph
O
O
O
N
O
O
Me3Si
HO
O

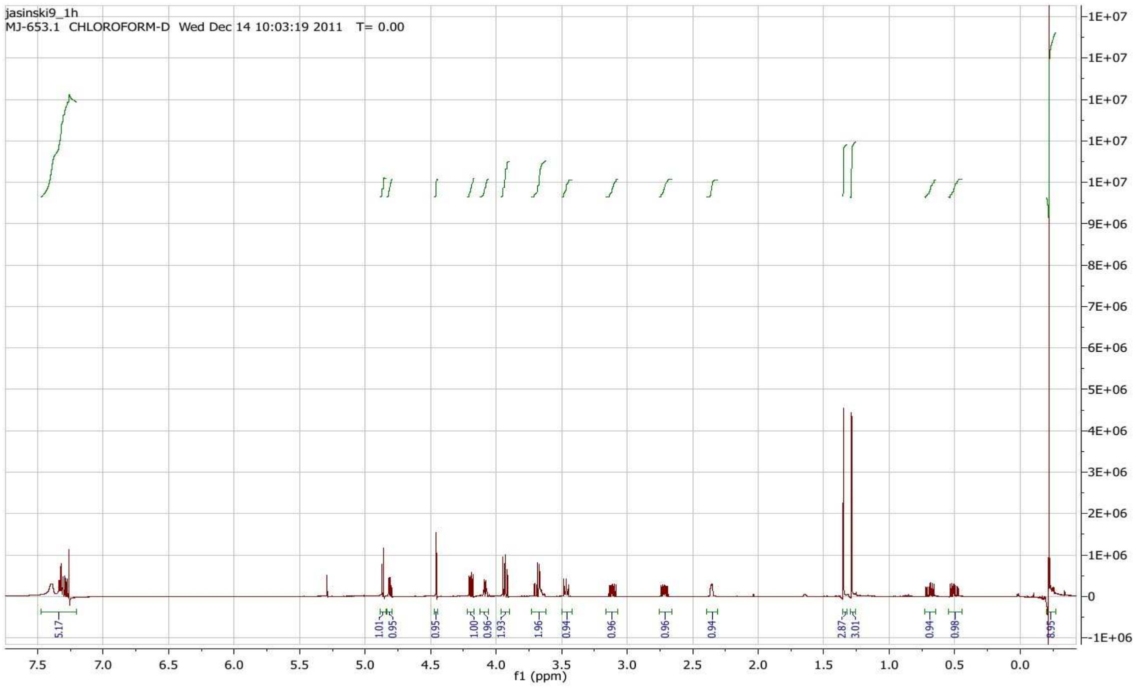

jasinski9_1h
MJ-653.1 CHLOROFORM-D Wed Dec 14 10:03:19 2011 T= 0.00
f1 (ppm)

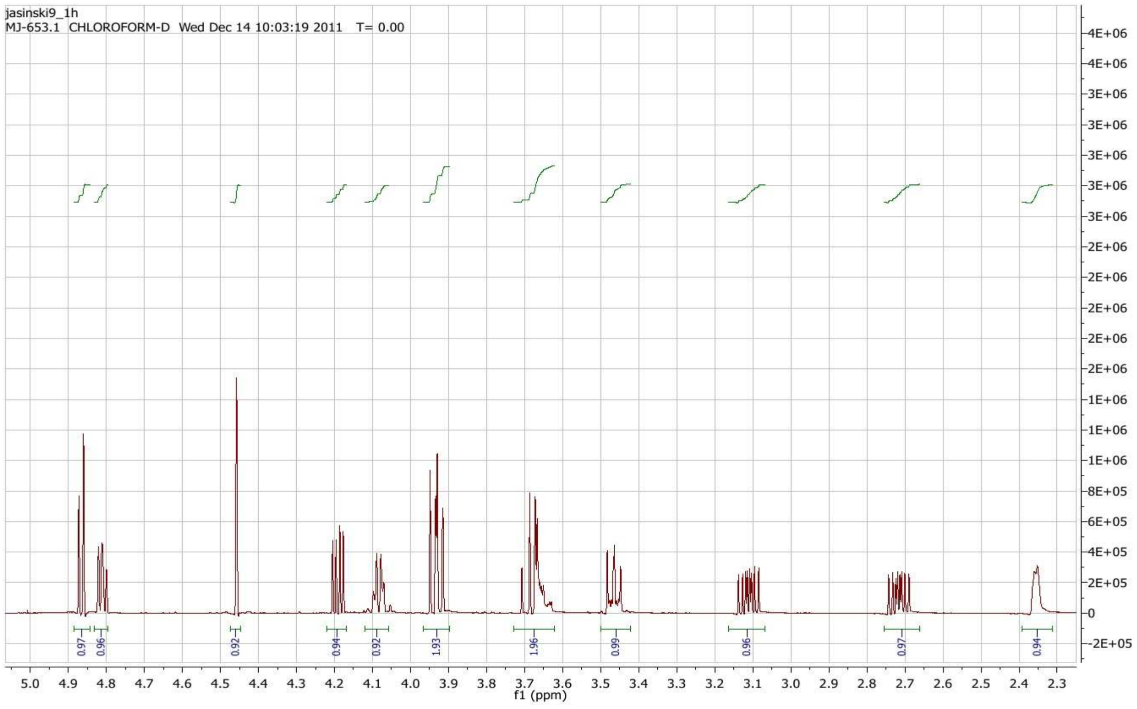

jasinski9_1h
MJ-653.1 CHLOROFORM-D Wed Dec 14 10:03:19 2011 T= 0.00
f1 (ppm)

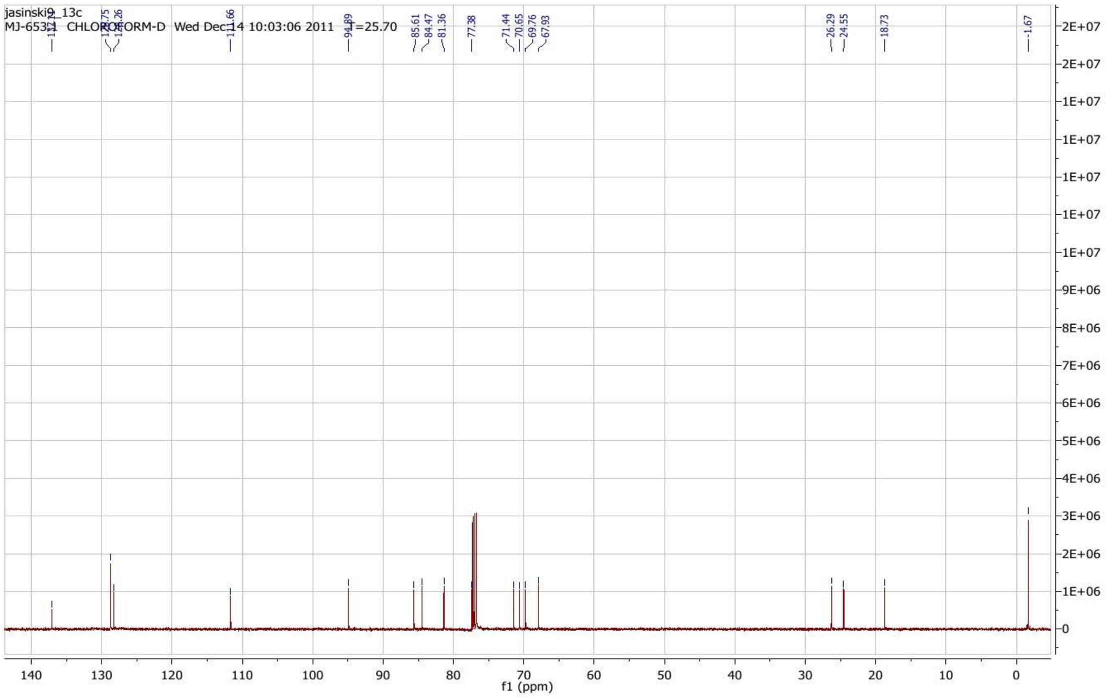
jasinski9_13c
MJ-653.1 CHLOROFORM-D Wed Dec 14 10:03:06 2011 T=25.70
111.66
94.89
85.61
84.47
81.36
77.38
71.44
70.65
69.76
67.93
26.29
24.55
18.73
-1.67
f1 (ppm)

Compound 9:

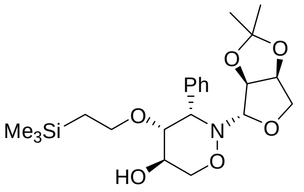
Ph
Me3Si
O
N
O
HO
O
O
O

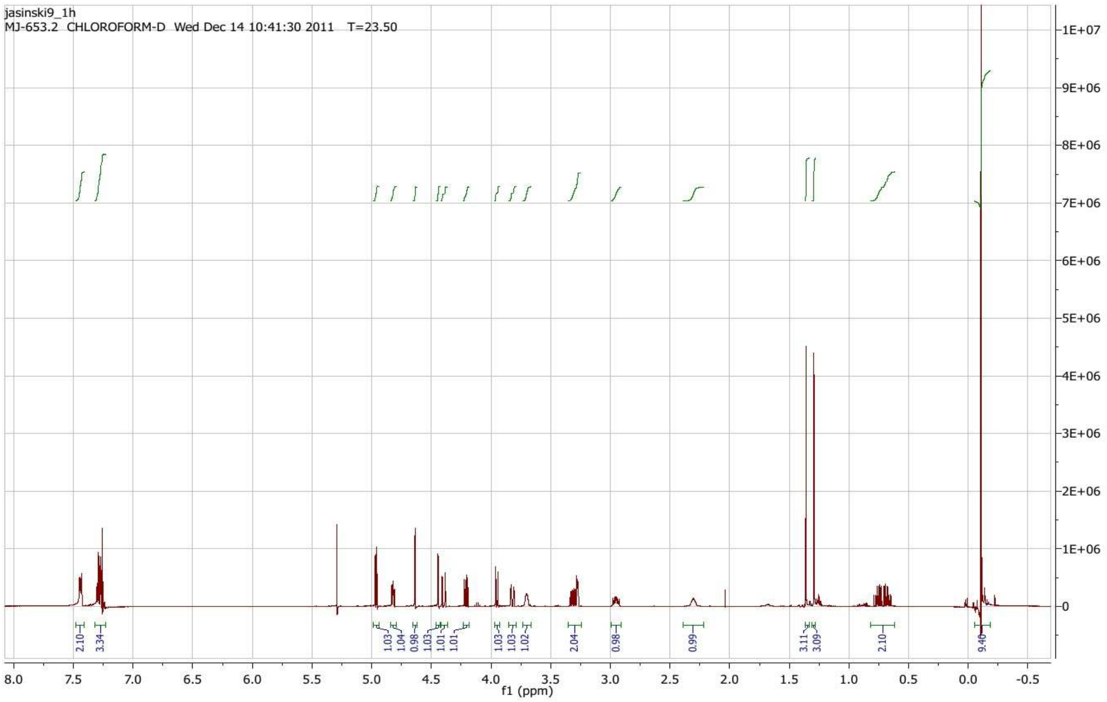
jasinski9_1h
MJ-653.2 CHLOROFORM-D Wed Dec 14 10:41:30 2011 T=23.50
f1 (ppm)

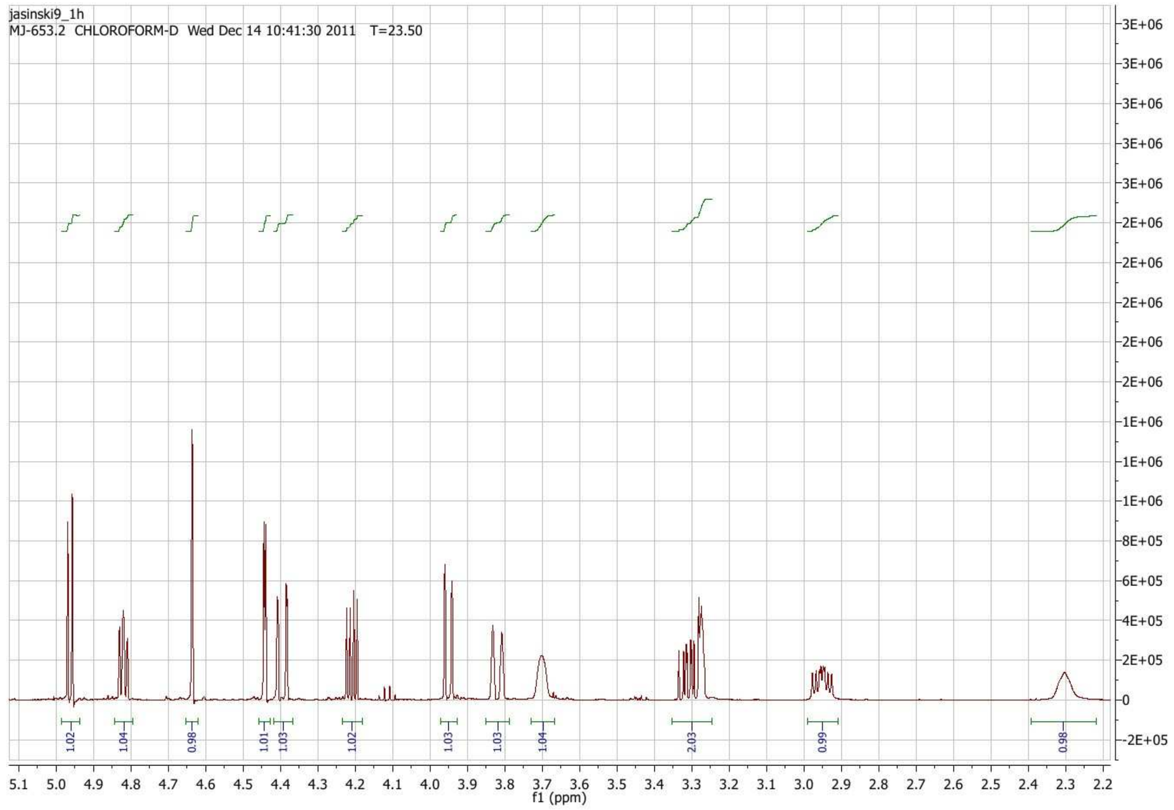
jasinski9_1h
MJ-653.2 CHLOROFORM-D Wed Dec 14 10:41:30 2011 T=23.50
1.02
1.04
0.98
1.01
1.03
1.02
1.03
1.03
1.04
2.03
0.99
0.98
f1 (ppm)

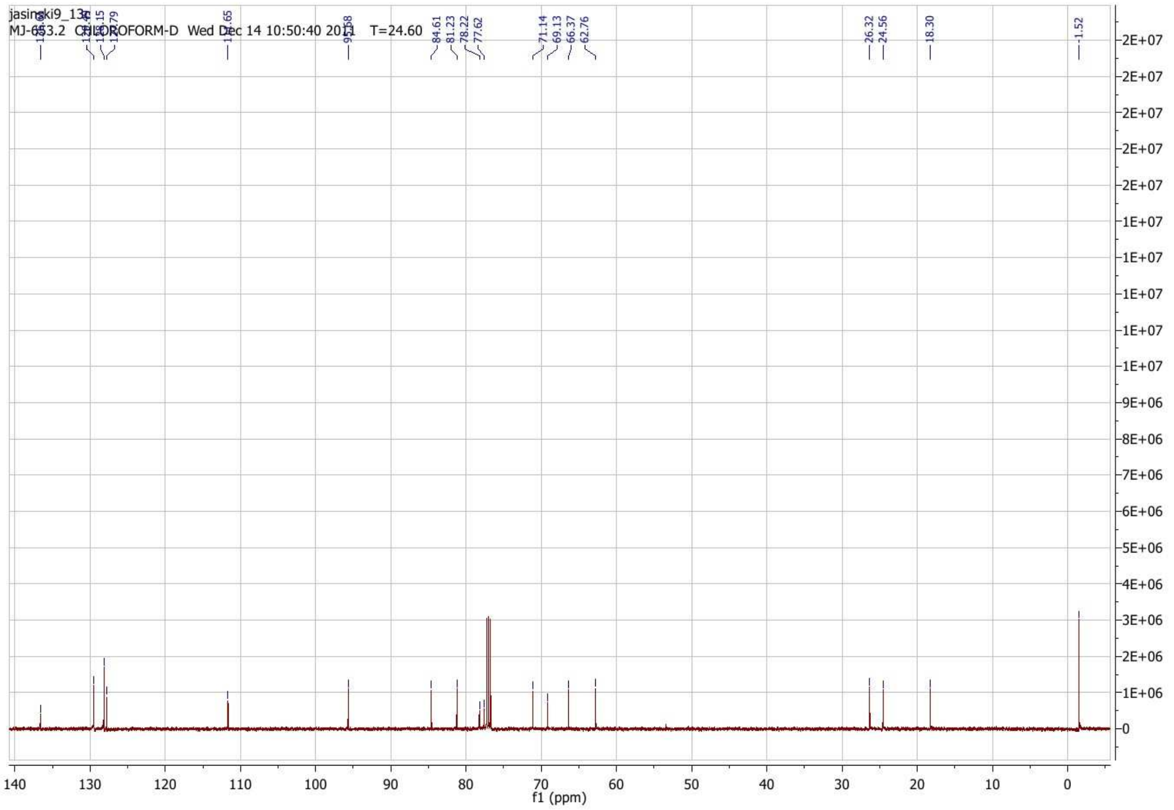
jasinski9_13c
MJ-653.2 CHLOROFORM-D Wed Dec 14 10:50:40 2011 T=24.60
84.61
81.23
78.22
77.62
71.14
69.13
66.37
62.76
26.32
24.56
18.30
-1.52
f1 (ppm)

Compound 10:

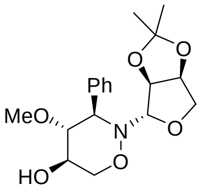
Ph
MeO
N
O
HO
O
O
O

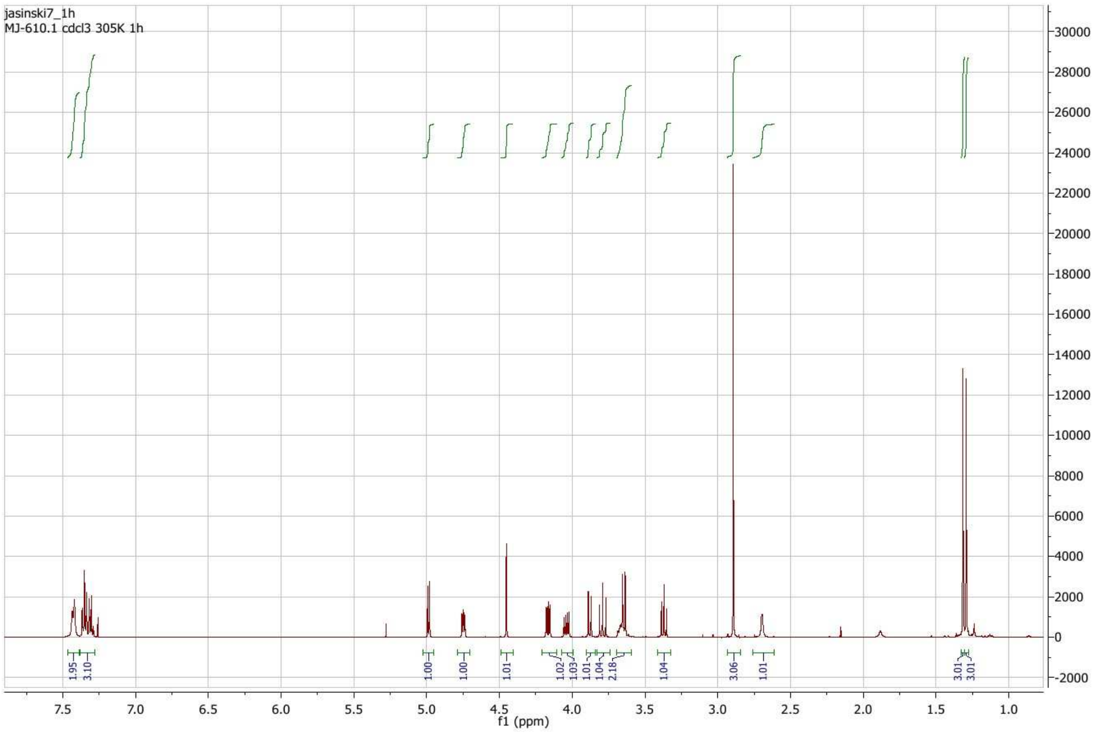
jasinski7_1h
MJ-610.1 cdcl3 305K 1h
f1 (ppm)

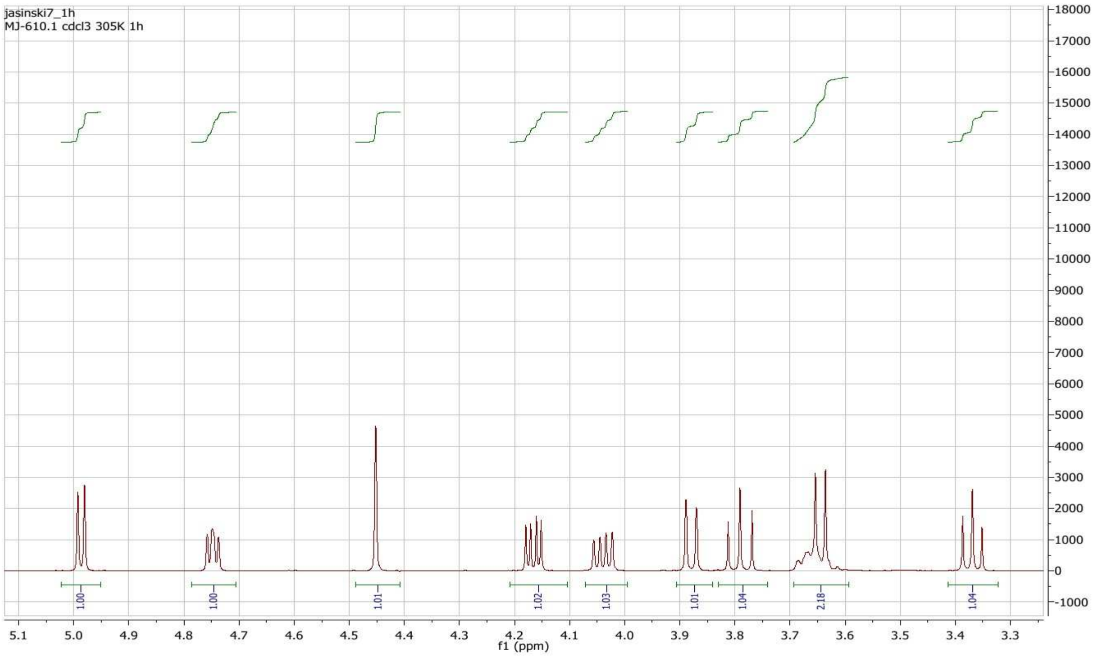
jasinski7_1h
MJ-610.1 cdcl3 305K 1h
f1 (ppm)

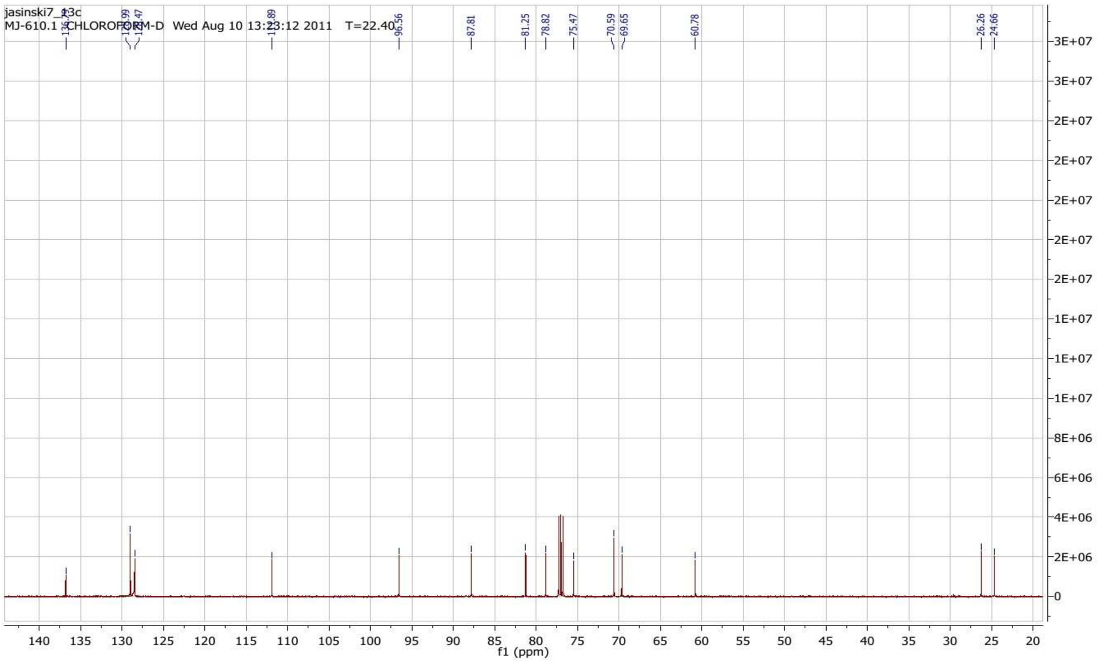
jasinski7_13c
MJ-610.1 CHLOROFORM-D Wed Aug 10 13:23:12 2011 T=22.40
96.56
87.81
81.25
78.82
75.47
70.59
69.65
60.78
26.26
24.66
f1 (ppm)

Compound 11:

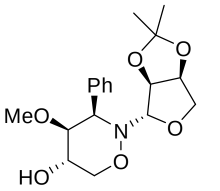
Ph
MeO
N
O
HO
O
O
O

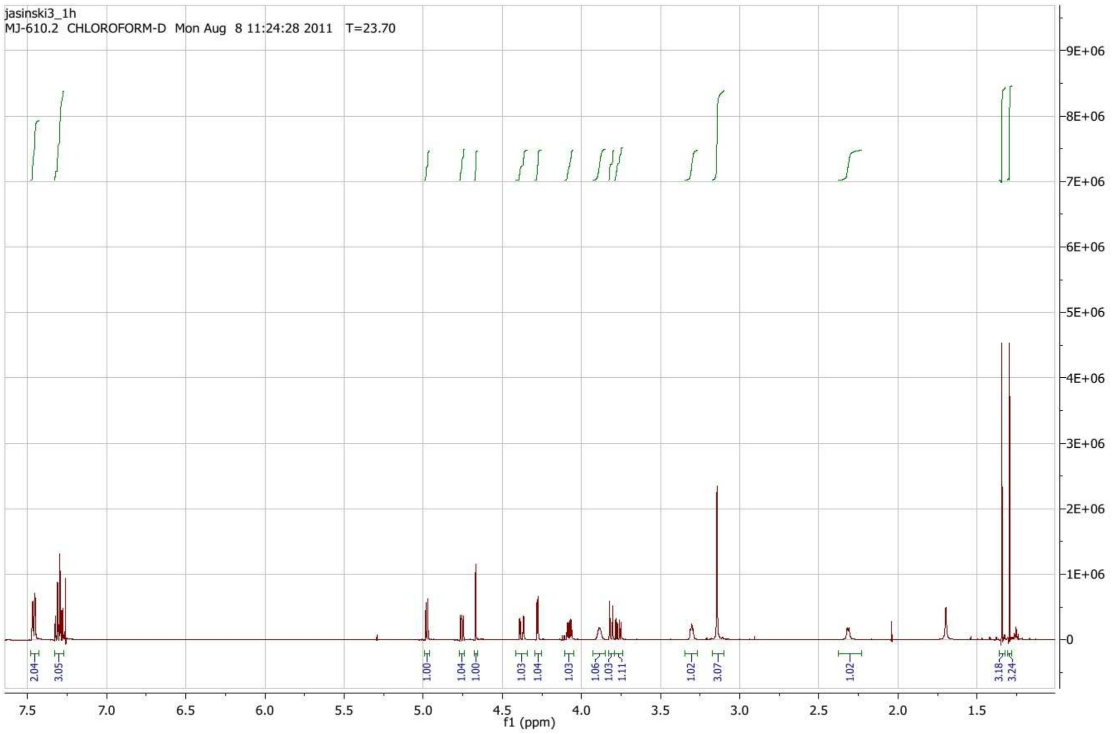
jasinski3_1h
MJ-610.2 CHLOROFORM-D Mon Aug 8 11:24:28 2011 T=23.70
2.04
3.05
1.00
1.04
1.00
1.03
1.04
1.03
1.06
1.03
1.11
1.02
3.07
1.02
3.18
3.24
f1 (ppm)

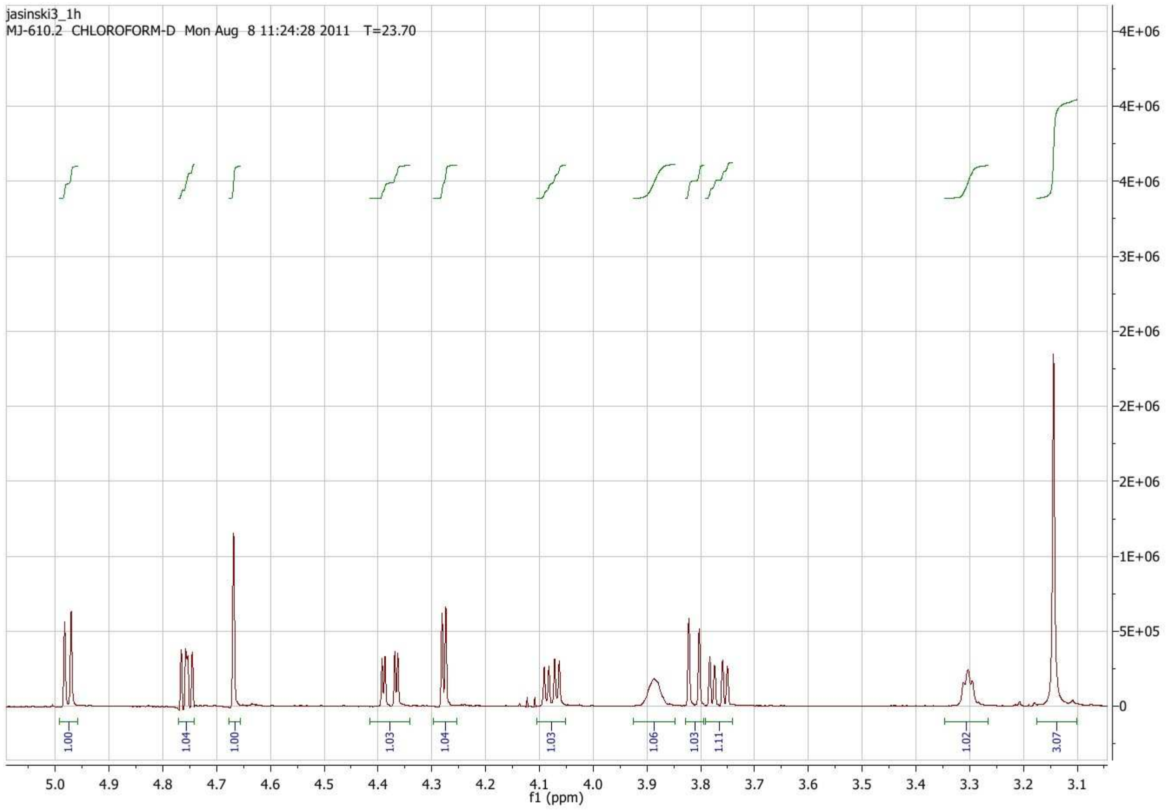

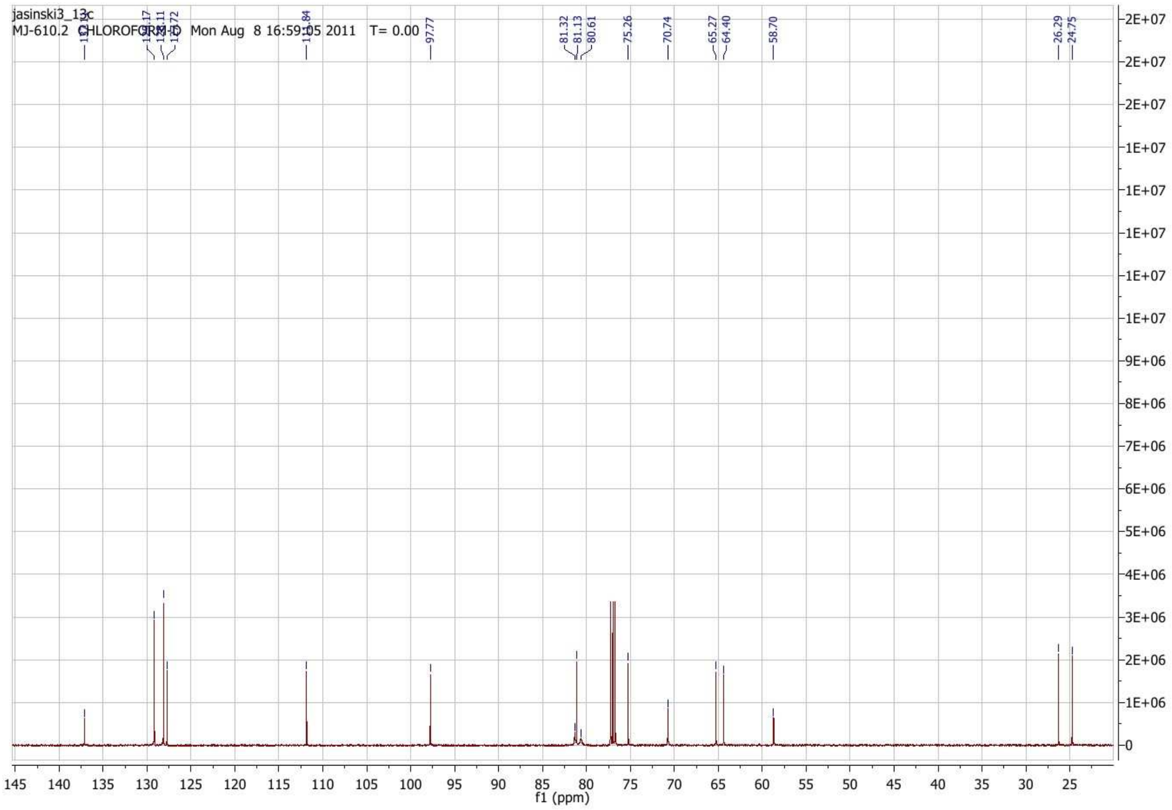

Compound 12:

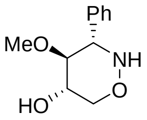
Ph
MeO
NH
O
HO

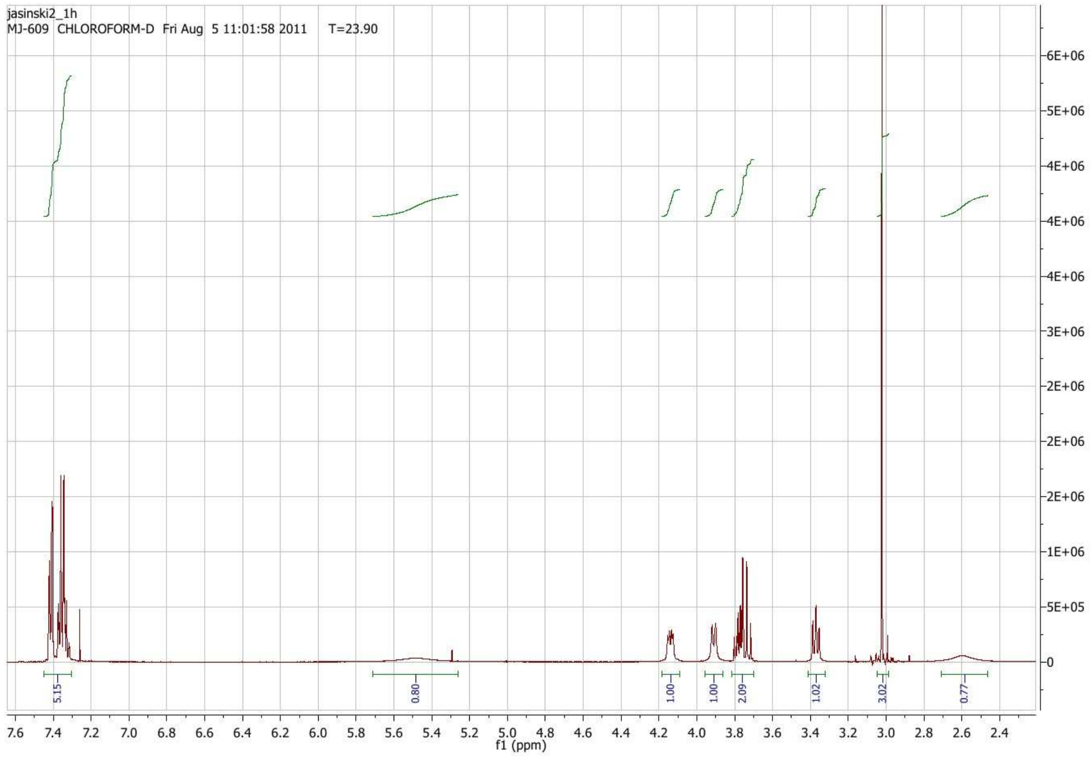
jasinski2_1h
MJ-609 CHLOROFORM-D Fri Aug 5 11:01:58 2011 T=23.90
5.15
0.80
1.00
1.00
2.09
1.02
3.02
0.77
f1 (ppm)

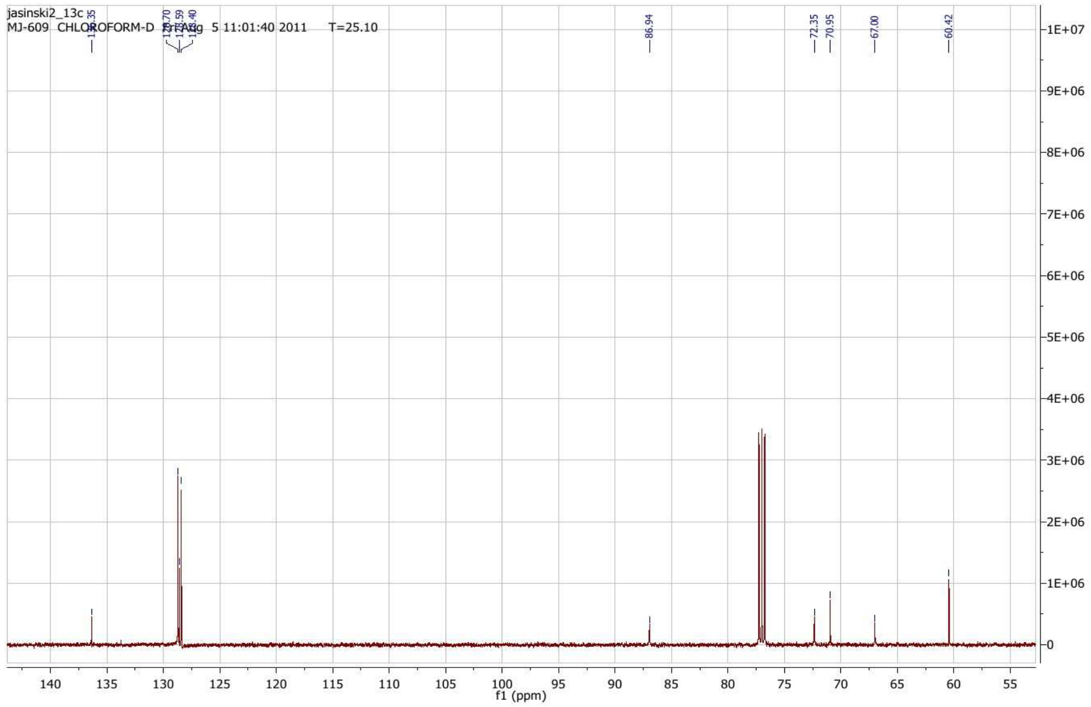
jasinski2_13c
MJ-609 CHLOROFORM-D Fri Aug 5 11:01:40 2011 T=25.10
86.94
72.35
70.95
67.00
60.42
f1 (ppm)

Compound 13:

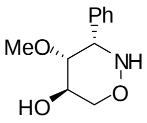
Ph
MeO
NH
O
HO

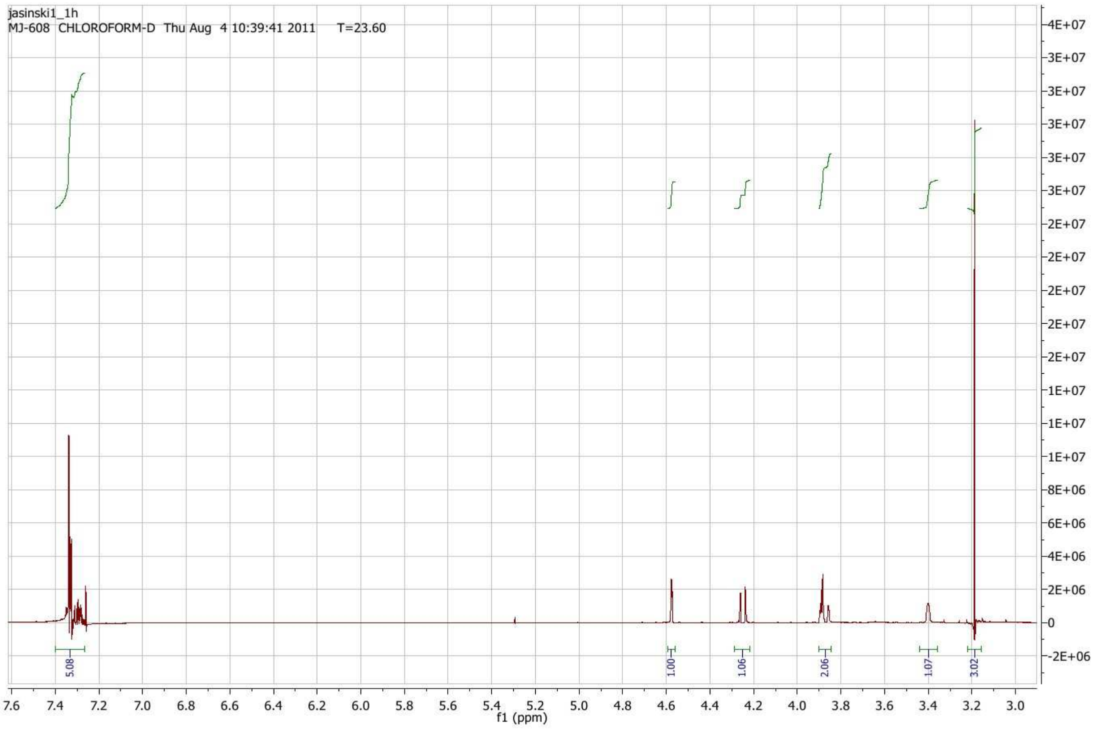
jasinski1_1h
MJ-608 CHLOROFORM-D Thu Aug 4 10:39:41 2011 T=23.60
5.08
1.00
1.06
2.06
1.07
3.02
f1 (ppm)

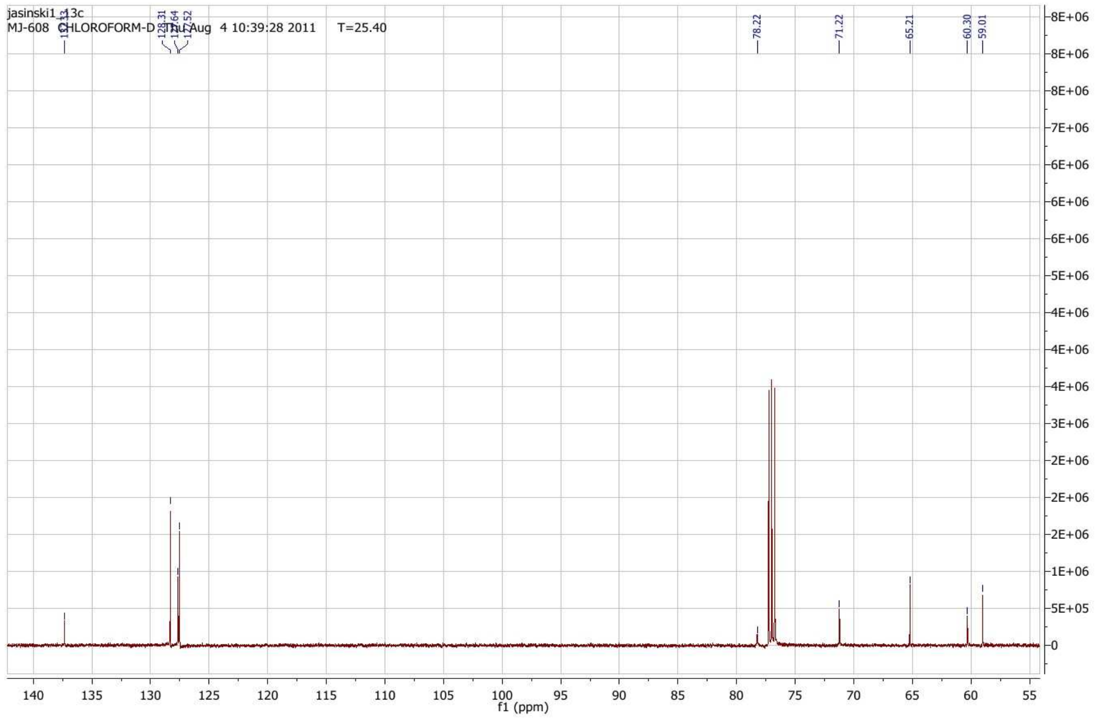
jasinski1_13c
MJ-608 CHLOROFORM-D Thu Aug 4 10:39:28 2011 T=25.40
128.31
127.64
127.52
78.22
71.22
65.21
60.30
59.01
f1 (ppm)

Compound 14:

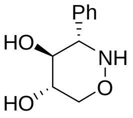
Ph
HO
NH
O
HO

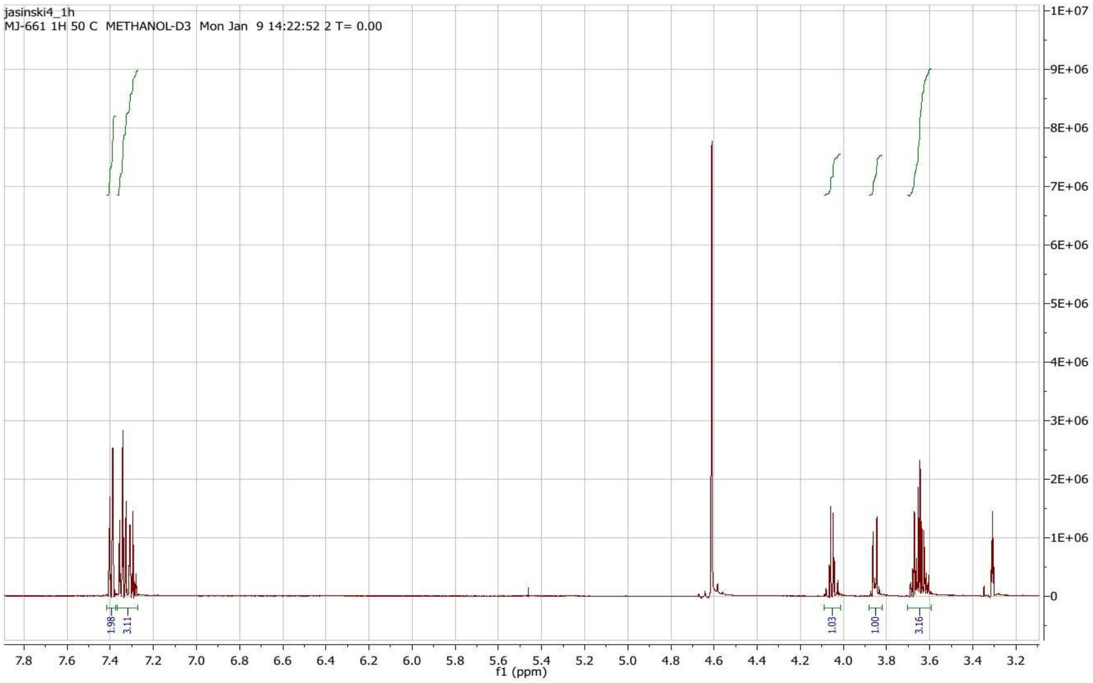
jasinski4_1h
MJ-661 1H 50 C METHANOL-D3 Mon Jan 9 14:22:52 2 T= 0.00
1.98
3.11
1.03
1.00
3.16
f1 (ppm)

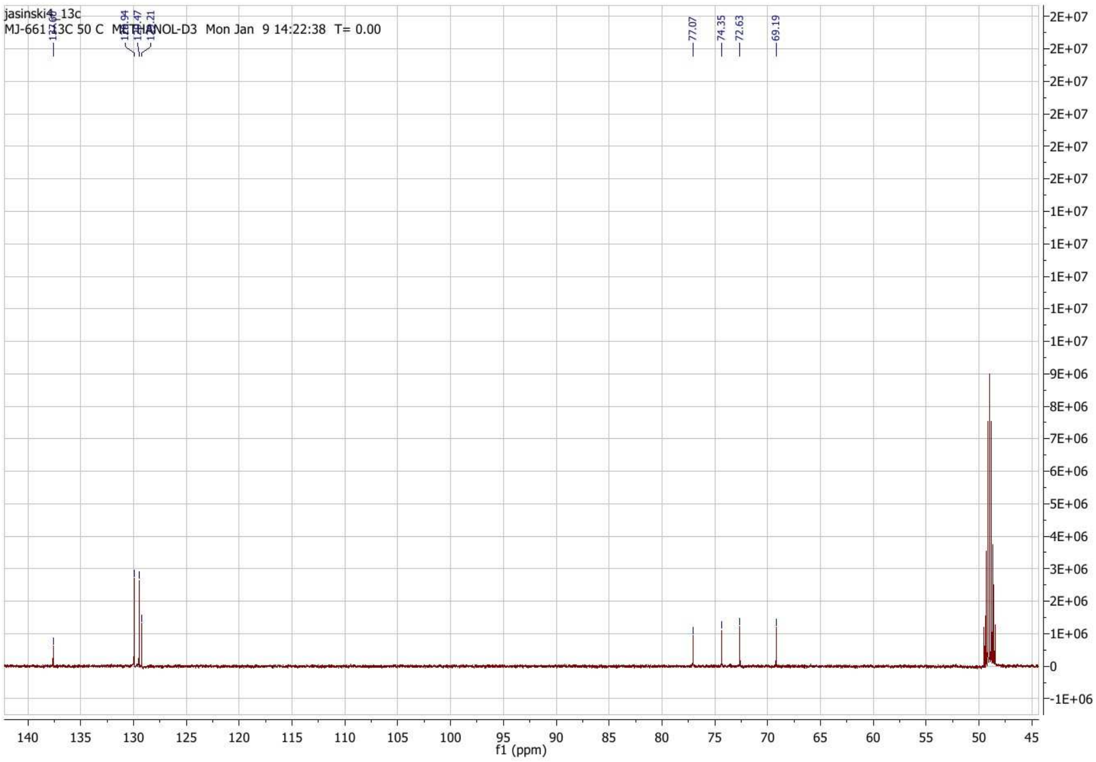
jasinski4_13c
MJ-661 13C 50 C METHANOL-D3 Mon Jan 9 14:22:38 T= 0.00
77.07
74.35
72.63
69.19
f1 (ppm)

Diagnostic part of the ^{1}H NMR spectrum of **14** measured at room temperature

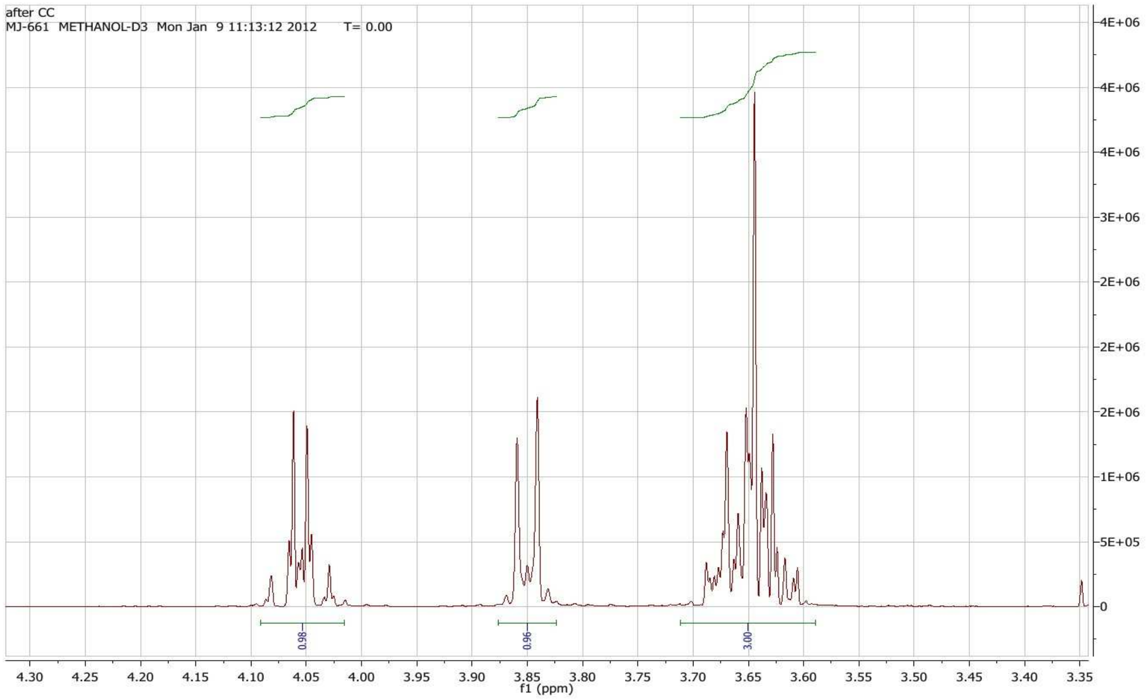

^{1}H NMR spectrum of **14** taken in the presence of BBr_3

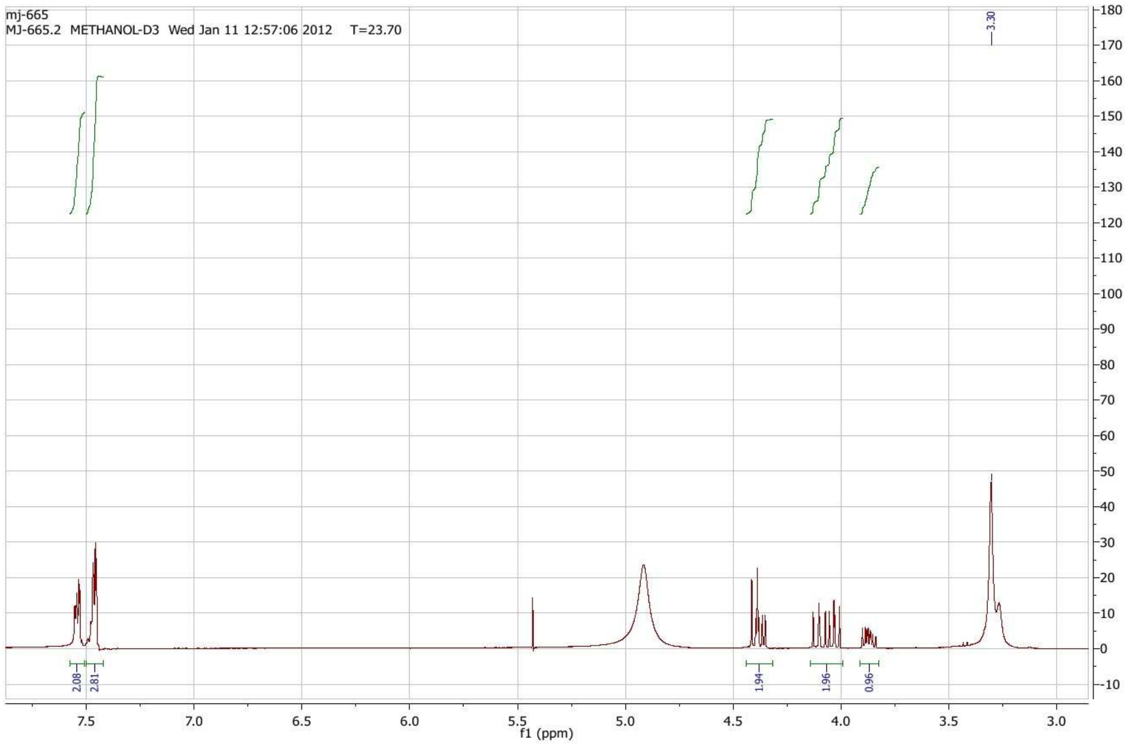

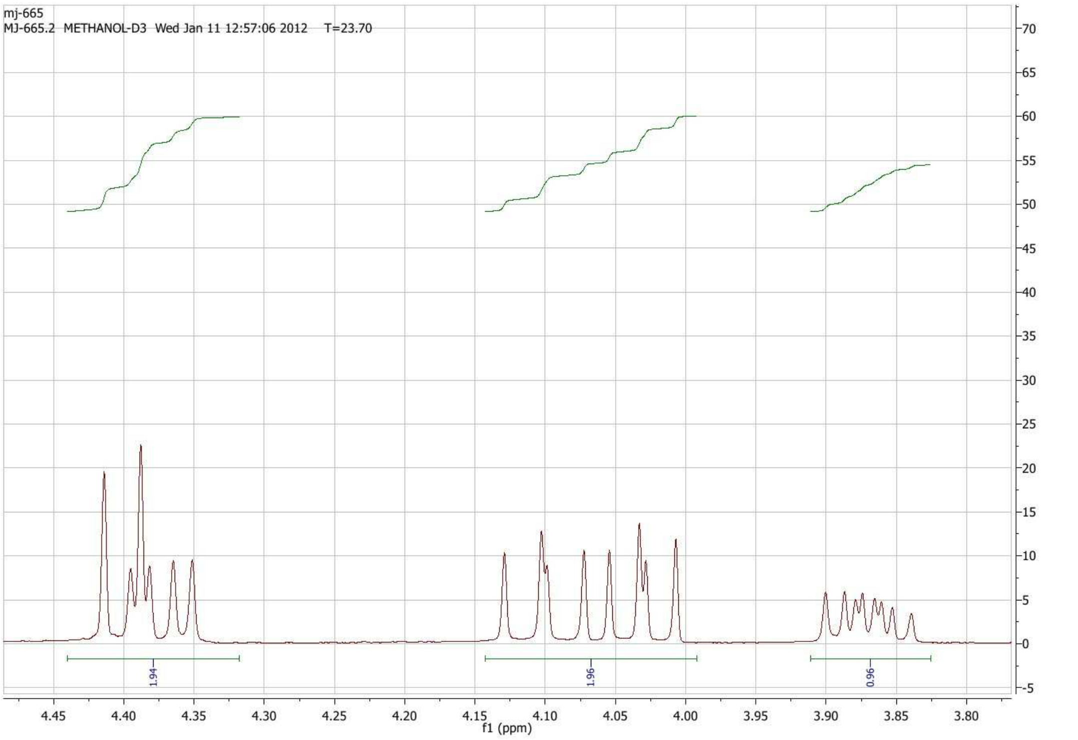

(3*S*,4*R*,5*R*)-3-Phenyl-4-(2-trimethylsilylethoxy)-[1,2]oxazinan-5-ol

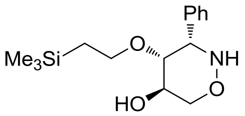

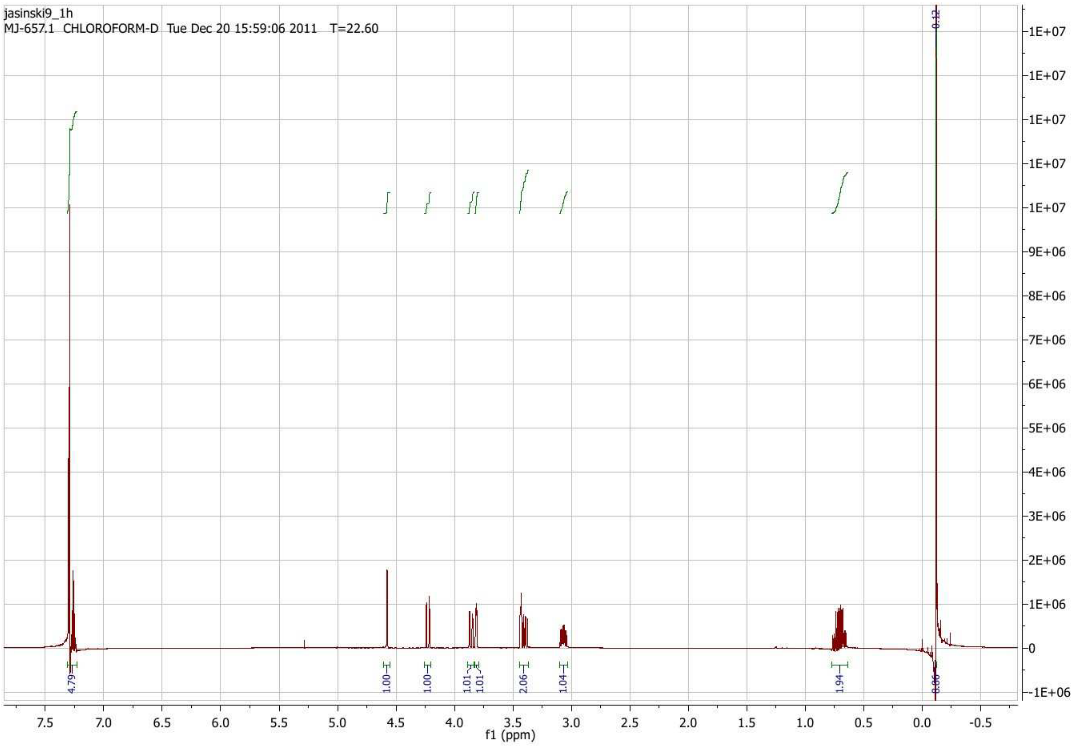

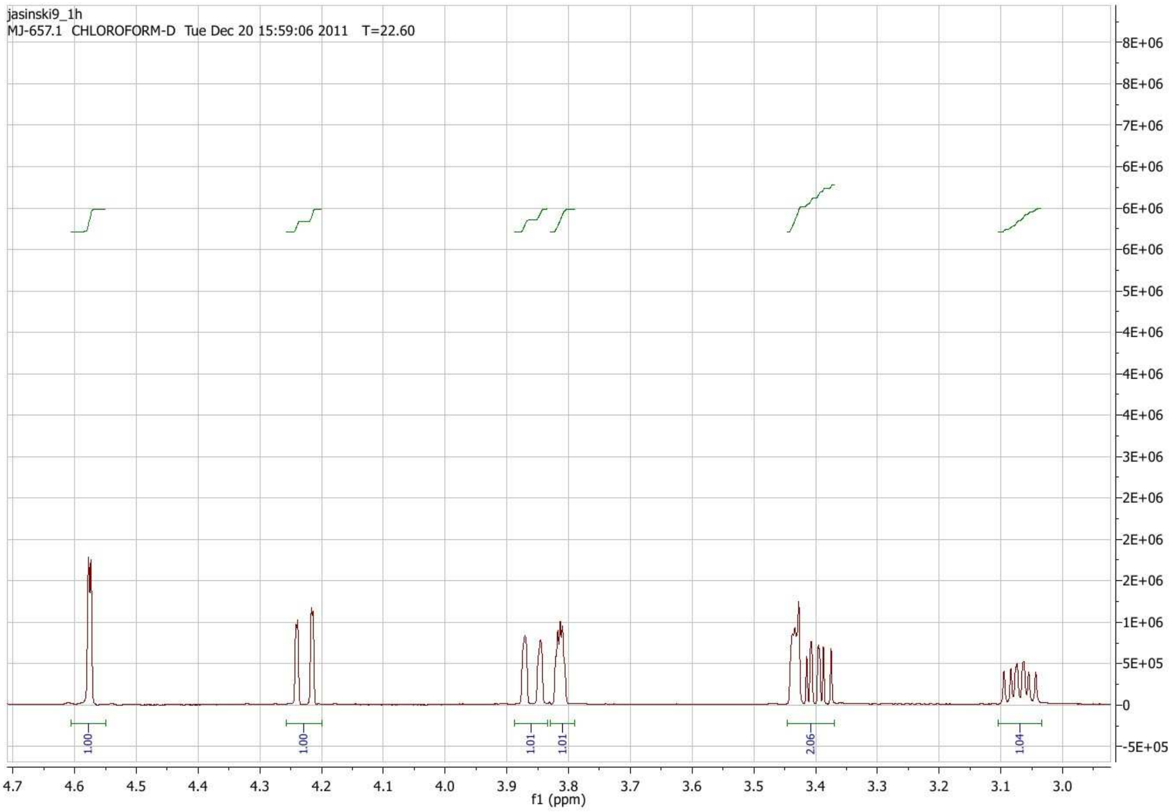
jasinski9_1h
MJ-657.1 CHLOROFORM-D Tue Dec 20 15:59:06 2011 T=22.60
1.00
1.00
1.01
1.01
2.06
1.04
f1 (ppm)

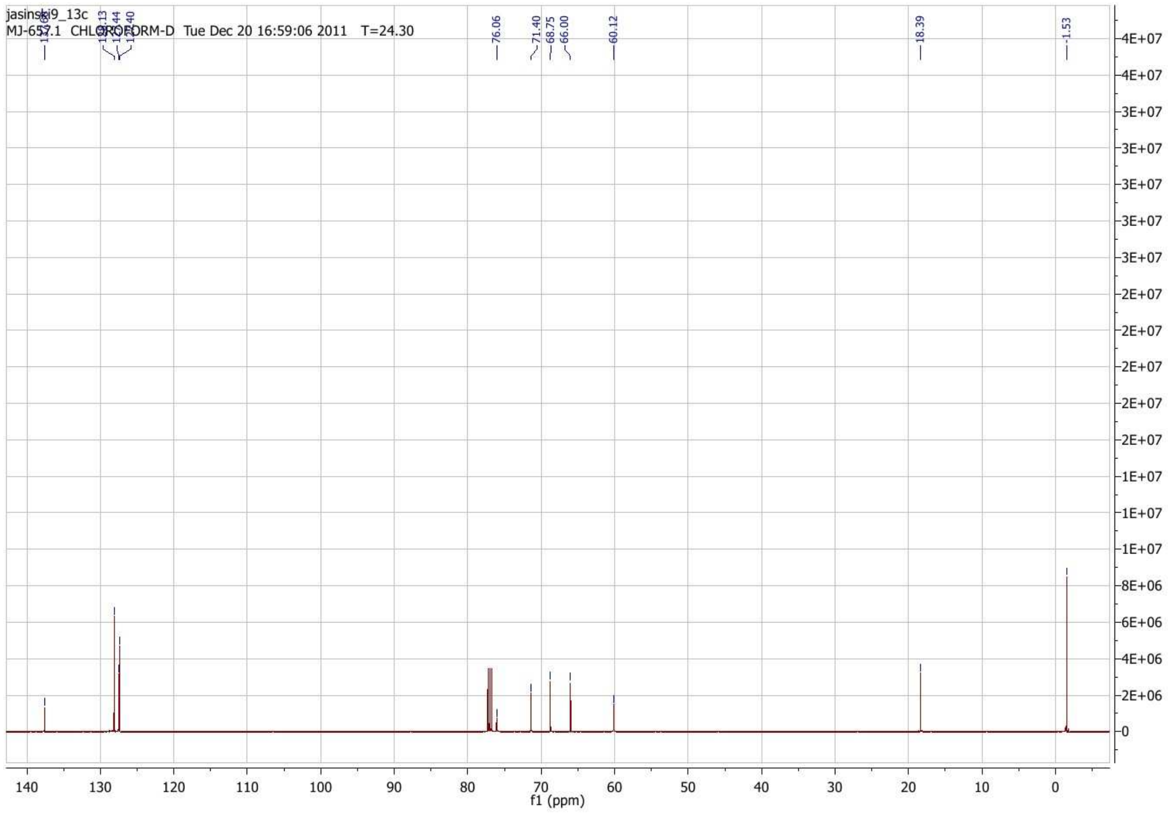
jasinski9_13c
MJ-657.1 CHLOROFORM-D Tue Dec 20 16:59:06 2011 T=24.30
128.13
127.44
127.40
76.06
71.40
68.75
66.00
60.12
18.39
-1.53
f1 (ppm)

Compound 15:

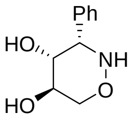
Ph
HO
NH
O
HO

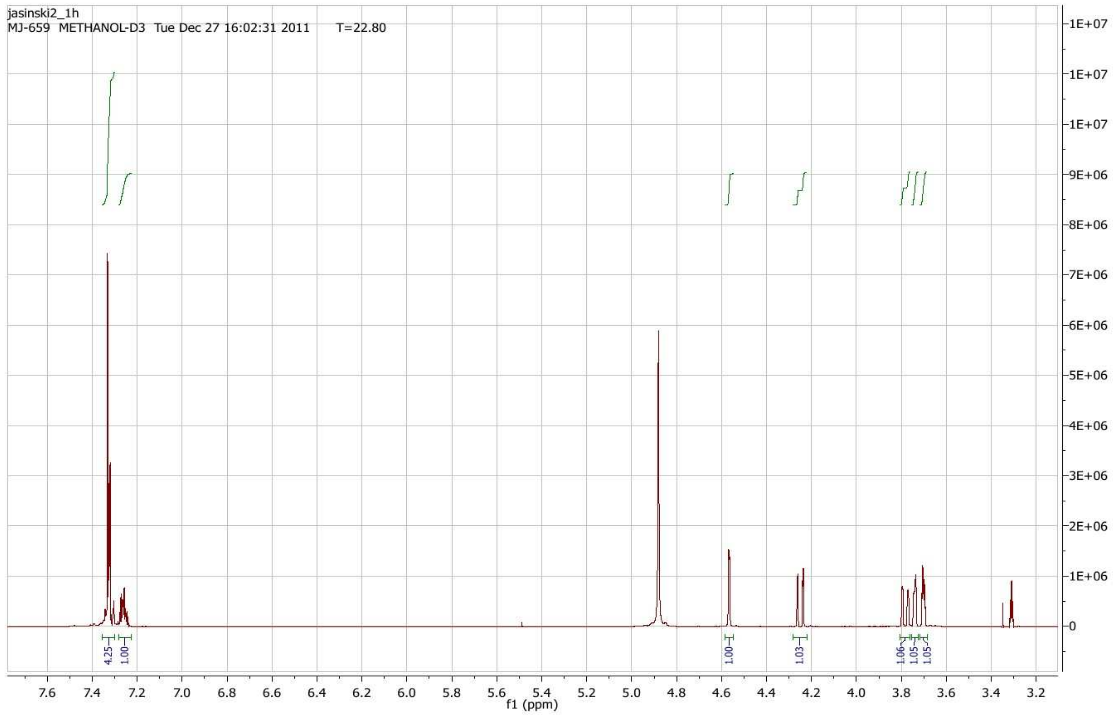
jasinski2_1h
MJ-659 METHANOL-D3 Tue Dec 27 16:02:31 2011 T=22.80
4.25
1.00
1.00
1.03
1.06
1.05
1.05
f1 (ppm)

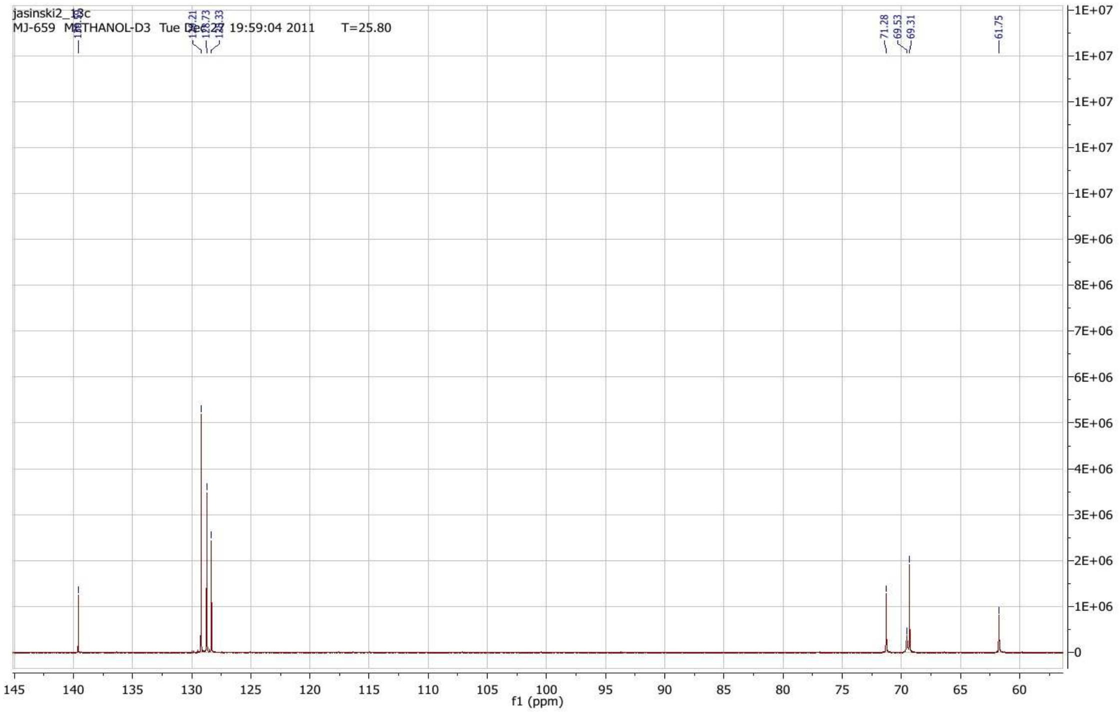
jasinski2_13c
MJ-659 METHANOL-D3 Tue Dec 27 19:59:04 2011 T=25.80
71.28
69.53
69.31
61.75
f1 (ppm)

Compound 16:

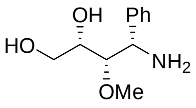
OH
Ph
HO
NH2
OMe

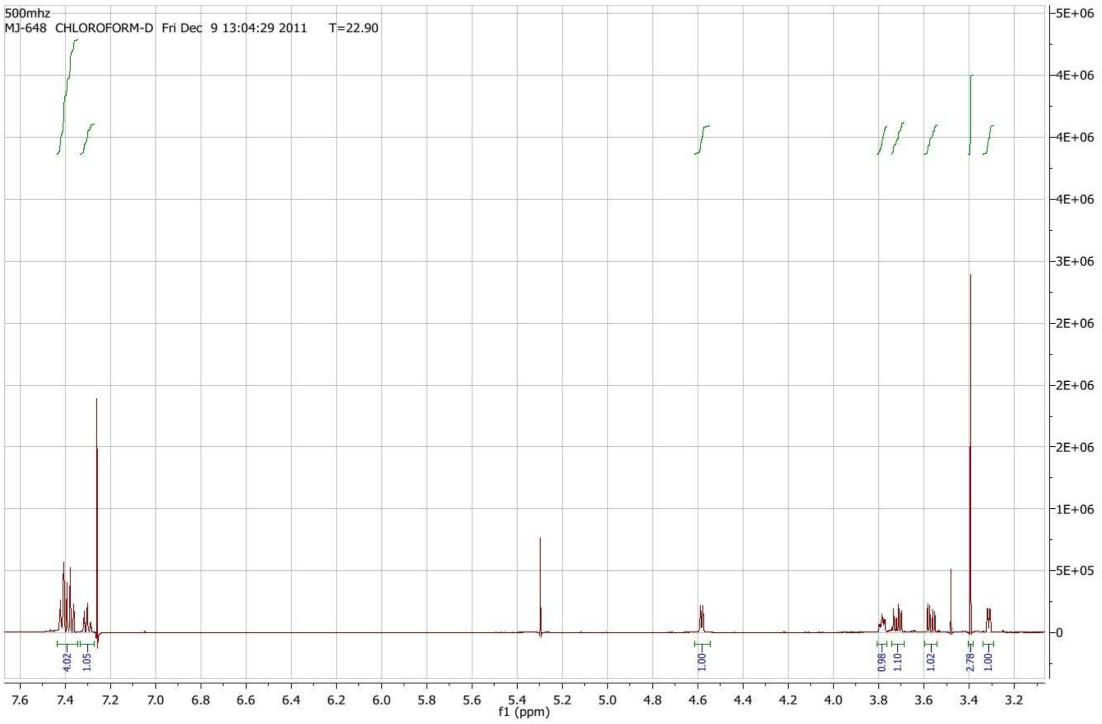
500mhz
MJ-648 CHLOROFORM-D Fri Dec 9 13:04:29 2011 T=22.90
4.02
1.05
1.00
0.98
1.10
1.02
2.78
1.00
f1 (ppm)

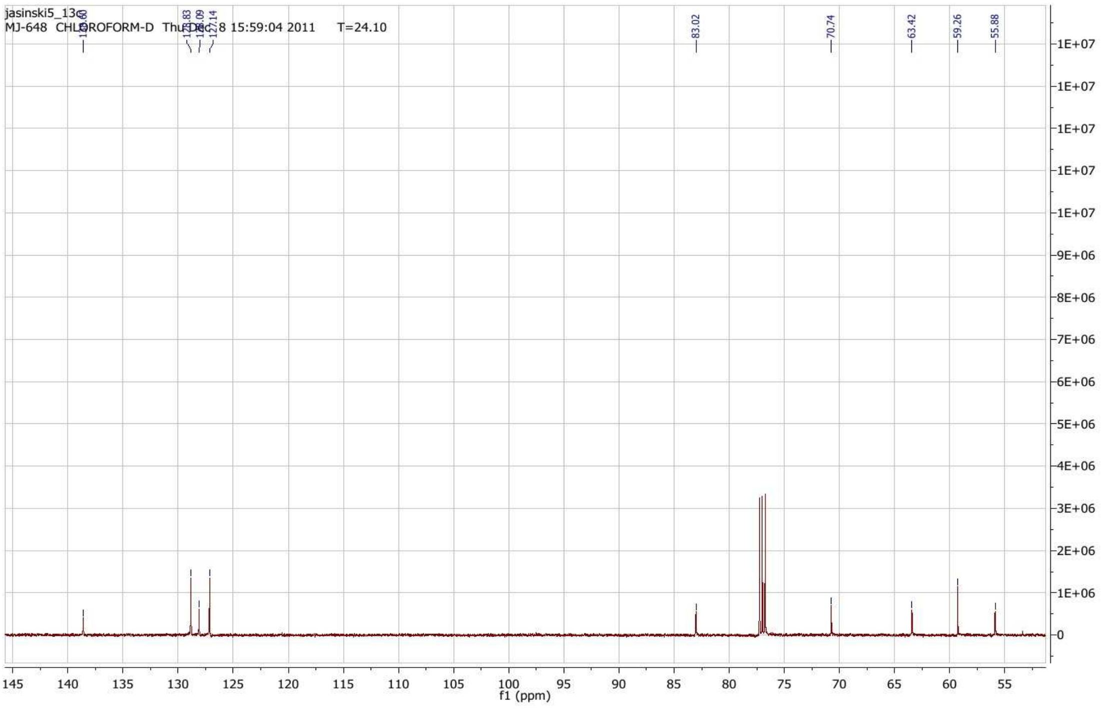
jasinski5_13c
MJ-648 CHLOROFORM-D Thu Dec 8 15:59:04 2011 T=24.10
127.14
83.02
70.74
63.42
59.26
55.88
f1 (ppm)

Compound 17:

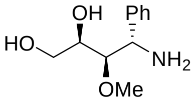
OH
Ph
HO
NH2
OMe

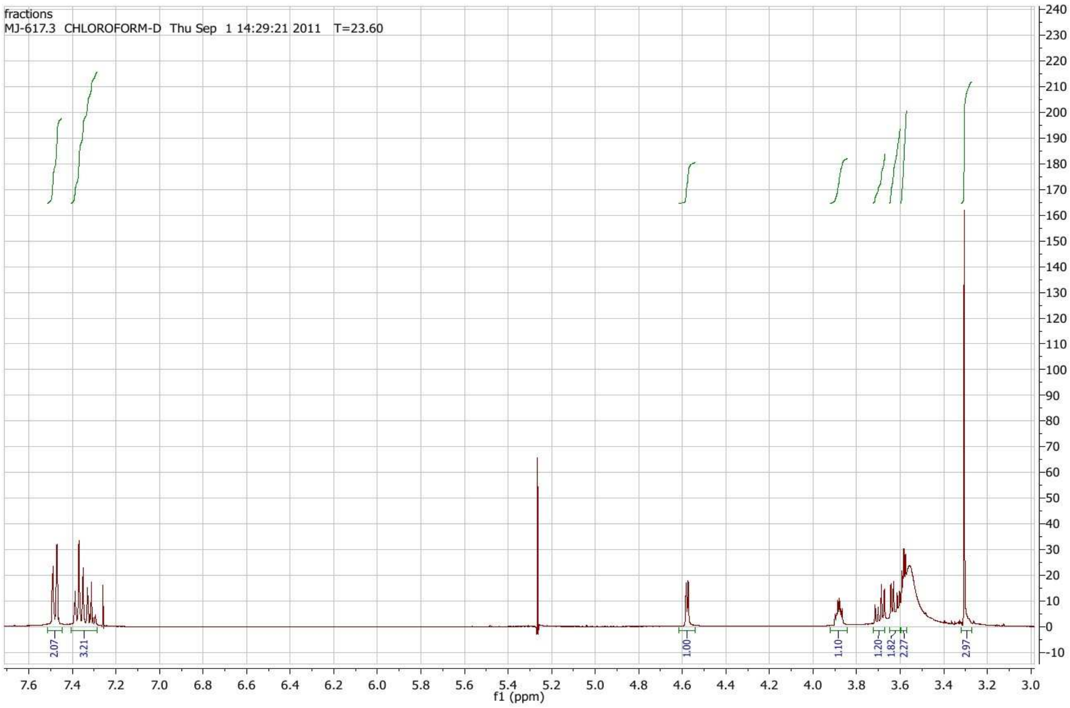
fractions
MJ-617.3 CHLOROFORM-D Thu Sep 1 14:29:21 2011 T=23.60
2.07
3.21
1.00
1.10
1.20
1.82
2.27
2.97
f1 (ppm)

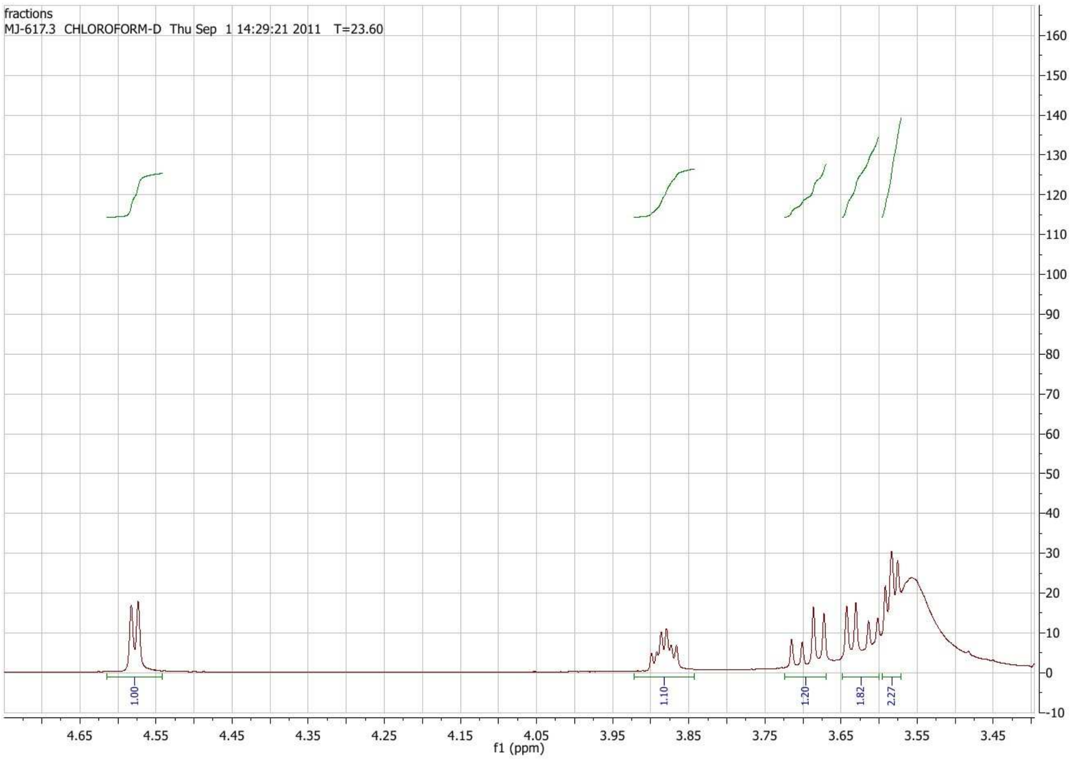
fractions
MJ-617.3 CHLOROFORM-D Thu Sep 1 14:29:21 2011 T=23.60
1.00
1.10
1.20
1.82
2.27
f1 (ppm)

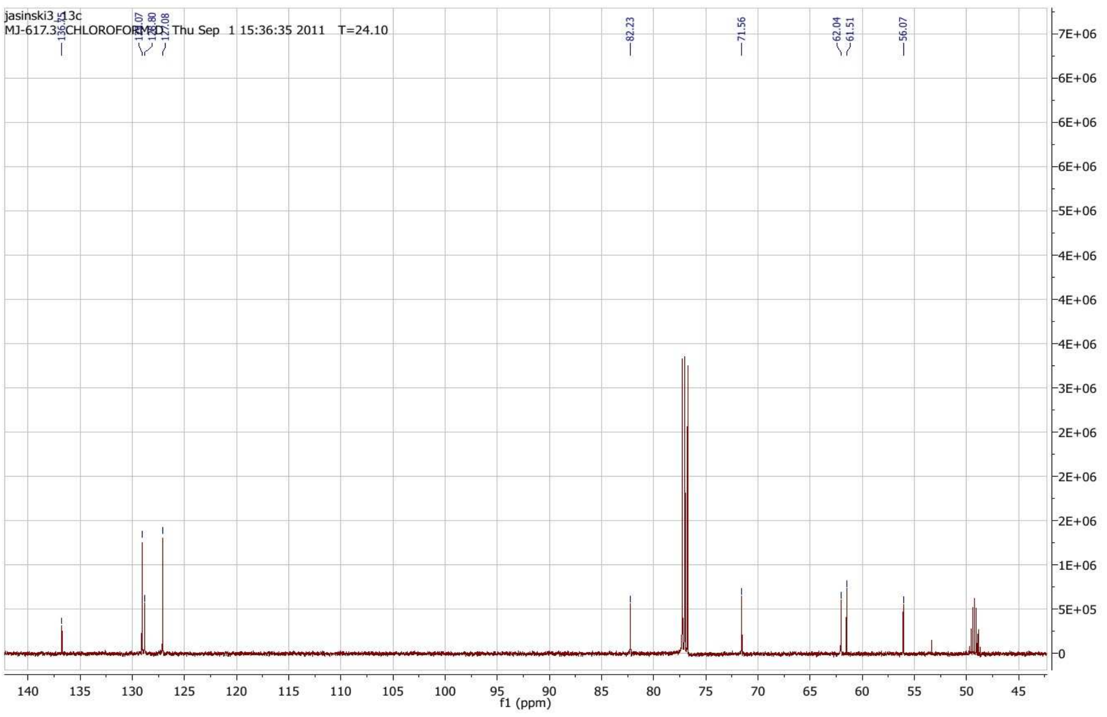

Compound 18:

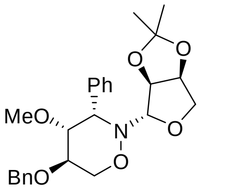

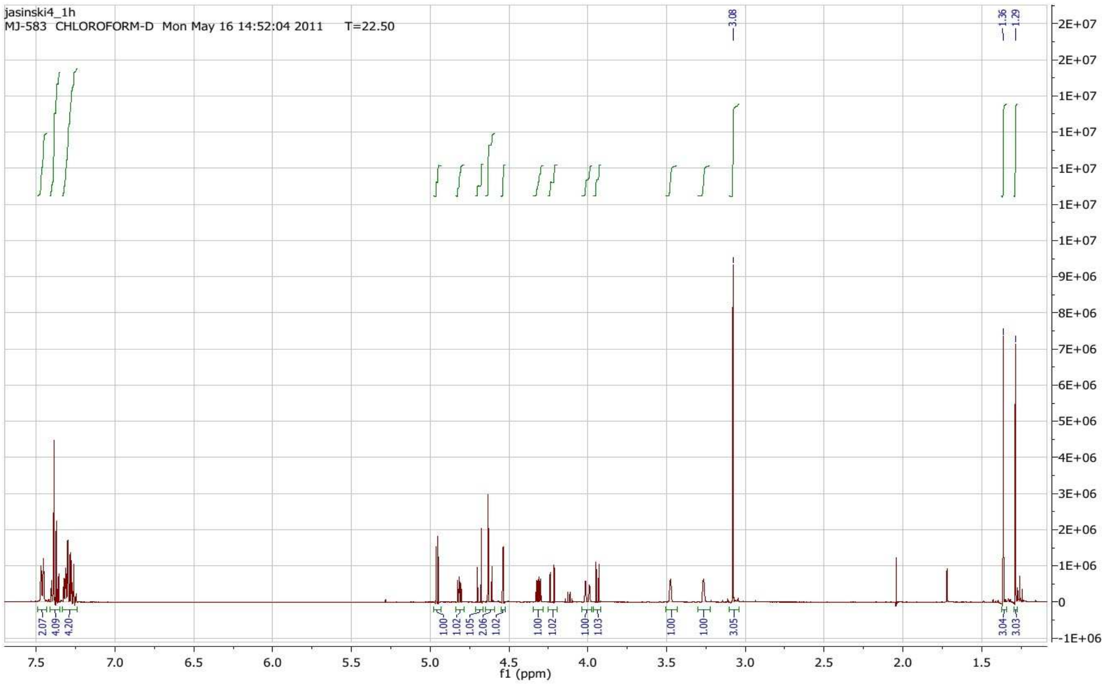

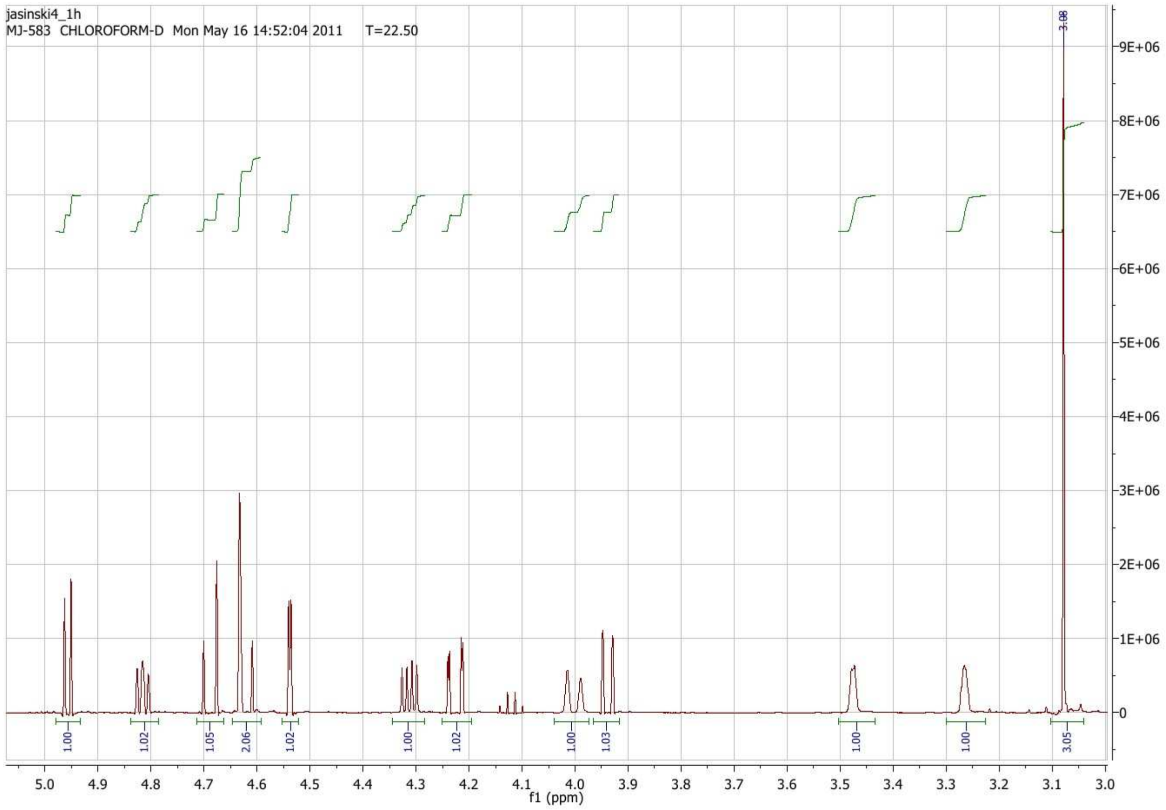
jasinski4_1h
MJ-583 CHLOROFORM-D Mon May 16 14:52:04 2011 T=22.50
1.00 1.02 1.05 2.06 1.02 1.00 1.02 1.00 1.03 1.00 1.00 3.05
f1 (ppm)

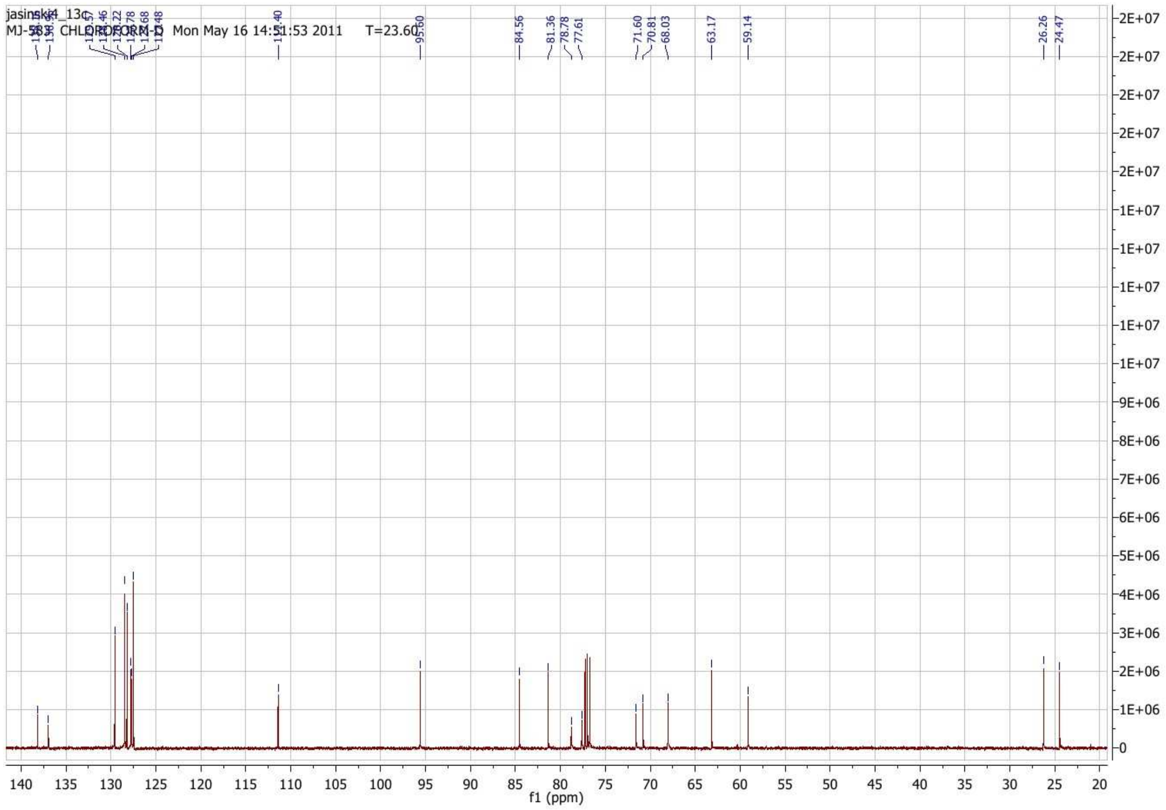
jasinski4_13c
MJ-583 CHLOROFORM-D Mon May 16 14:51:53 2011 T=23.60
84.56 81.36 78.78 77.61 71.60 70.81 68.03 63.17 59.14 26.26 24.47
f1 (ppm)

Compound 19:

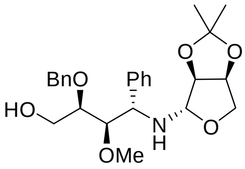

BnO
Ph
HO
N
H
O
O
O
OMe

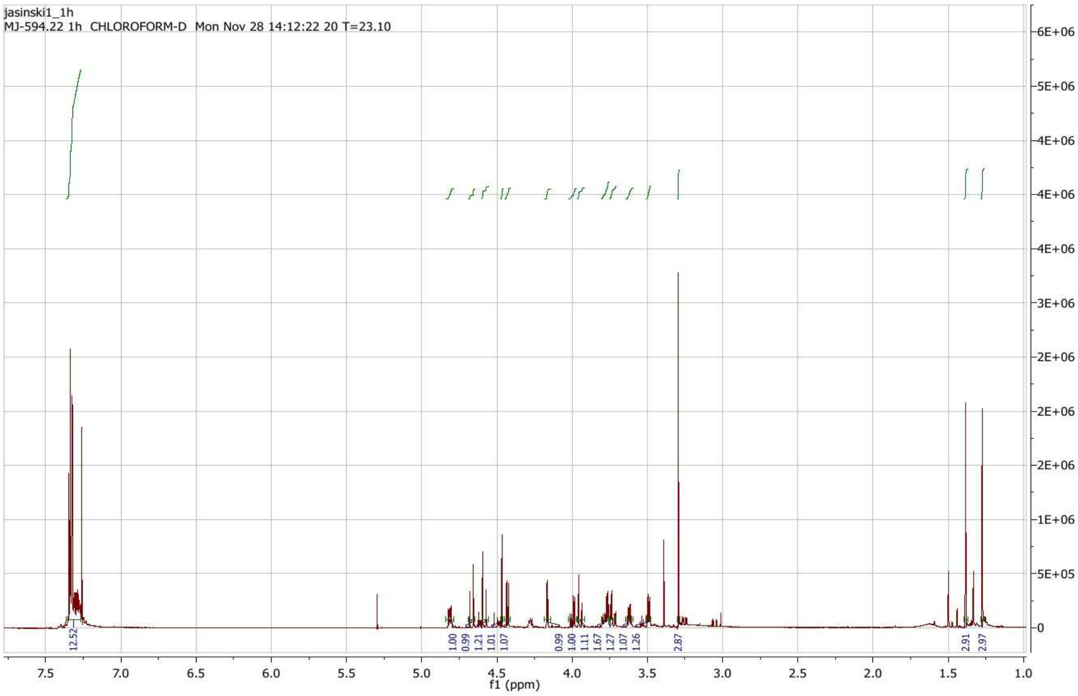

jasinski1_1h
MJ-594.22 1h CHLOROFORM-D Mon Nov 28 14:12:22 20 T=23.10
f1 (ppm)

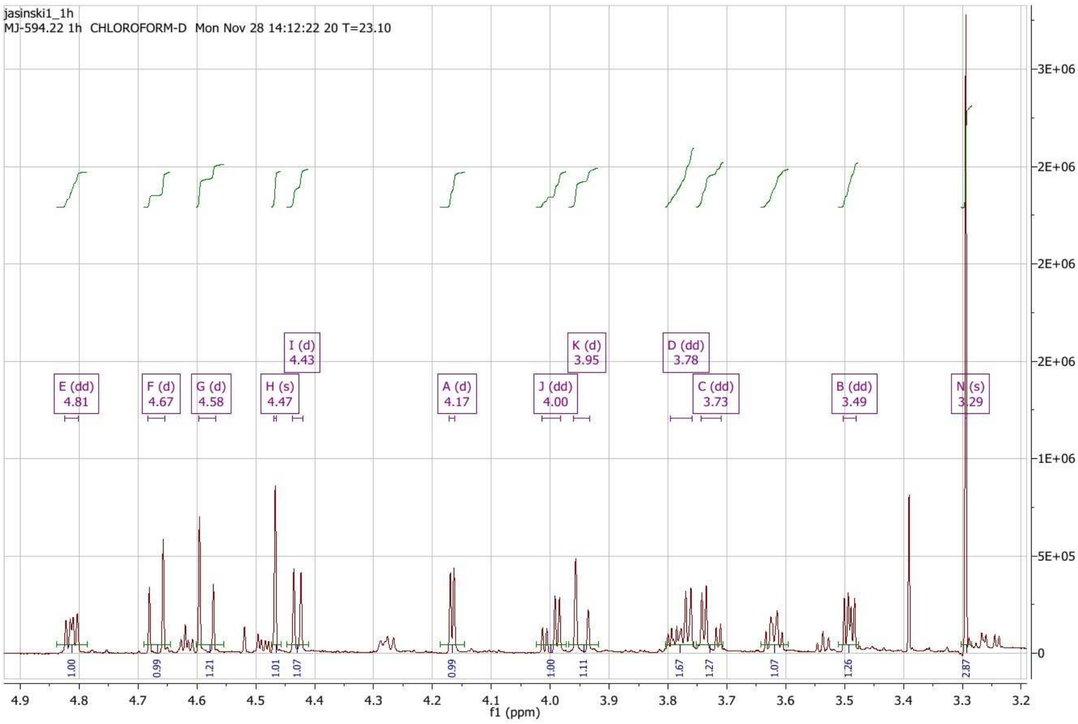

jasinski1_1h
MJ-594.22 1h CHLOROFORM-D Mon Nov 28 14:12:22 20 T=23.10
E (dd) 4.81
F (d) 4.67
G (d) 4.58
H (s) 4.47
I (d) 4.43
A (d) 4.17
J (dd) 4.00
K (d) 3.95
D (dd) 3.78
C (dd) 3.73
B (dd) 3.49
N (s) 3.29
f1 (ppm)

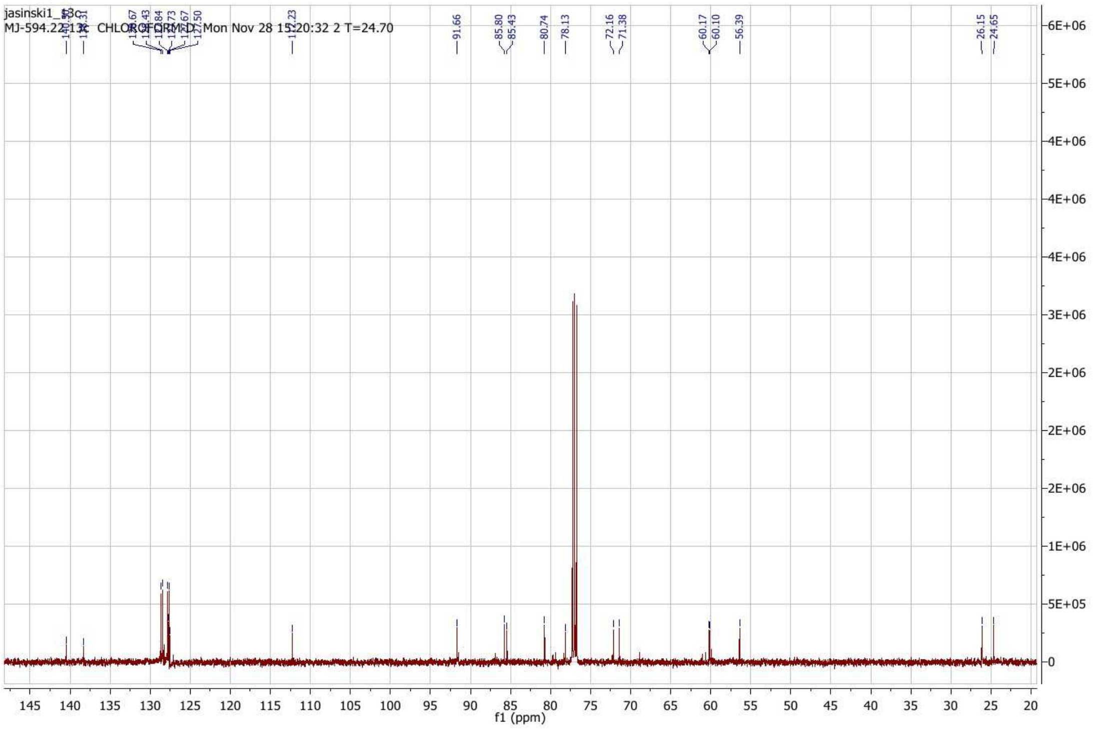

Compound 20:

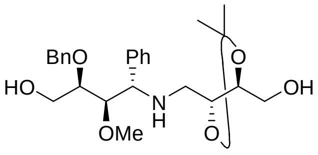

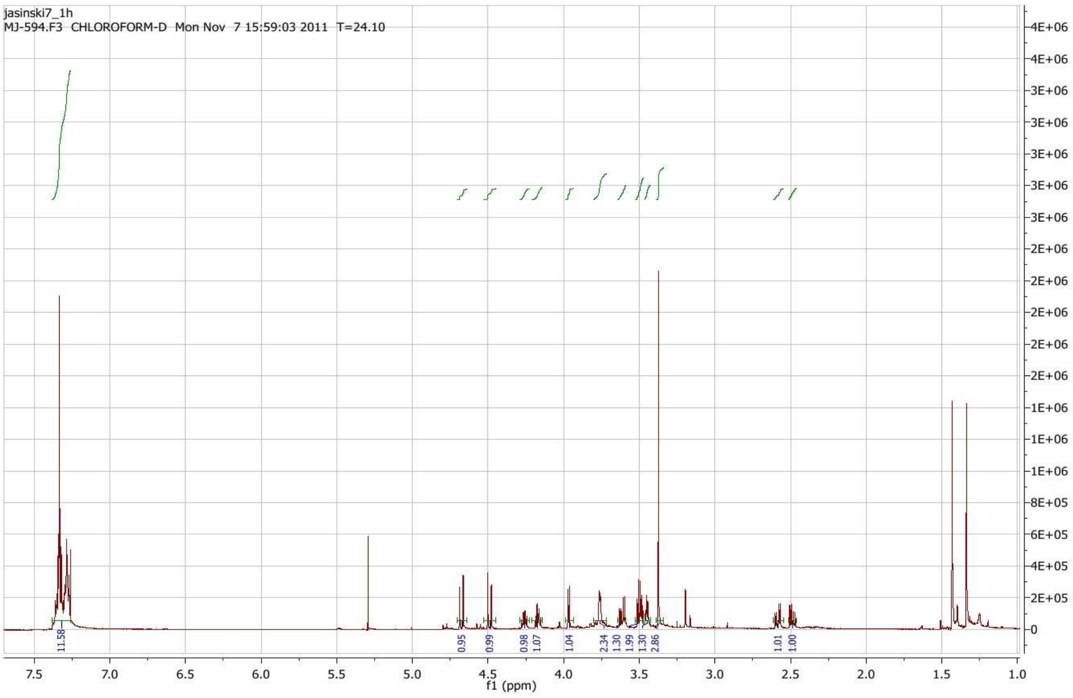

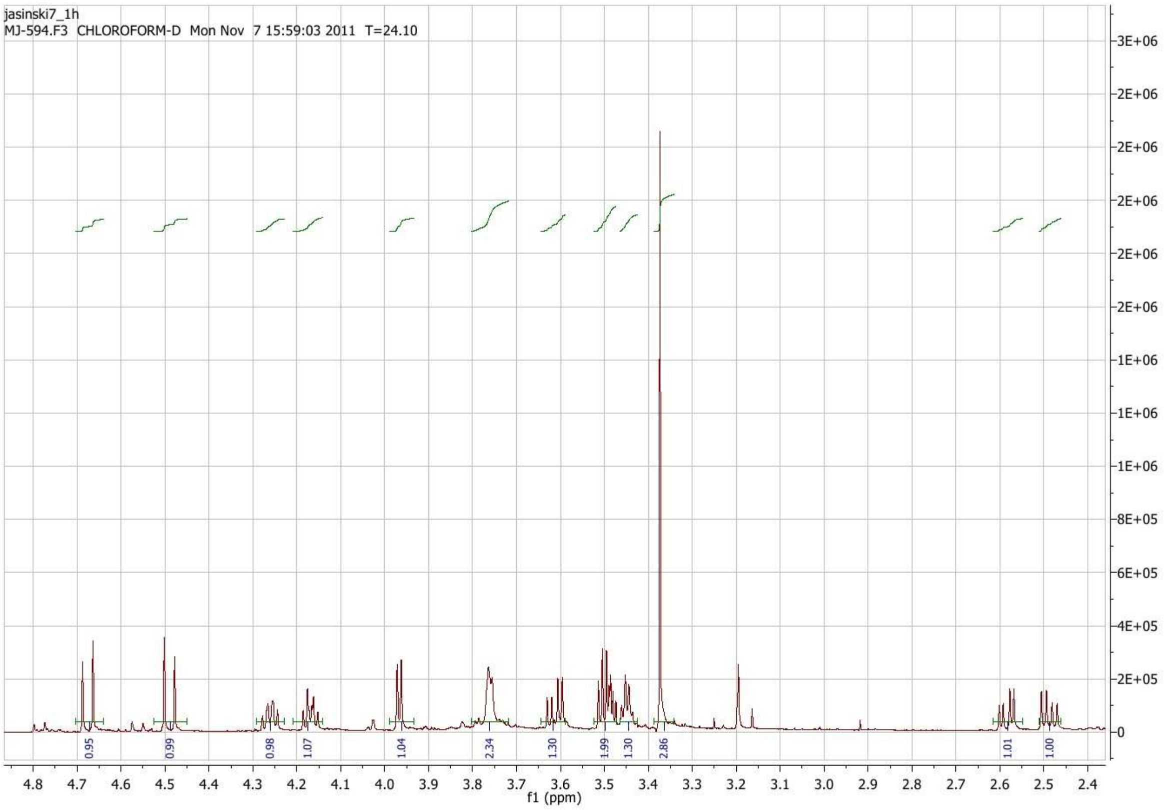
jasinski7_1h
MJ-594.F3 CHLOROFORM-D Mon Nov 7 15:59:03 2011 T=24.10
0.95
0.99
0.98
1.07
1.04
2.34
1.30
1.99
1.30
2.86
1.01
1.00
f1 (ppm)

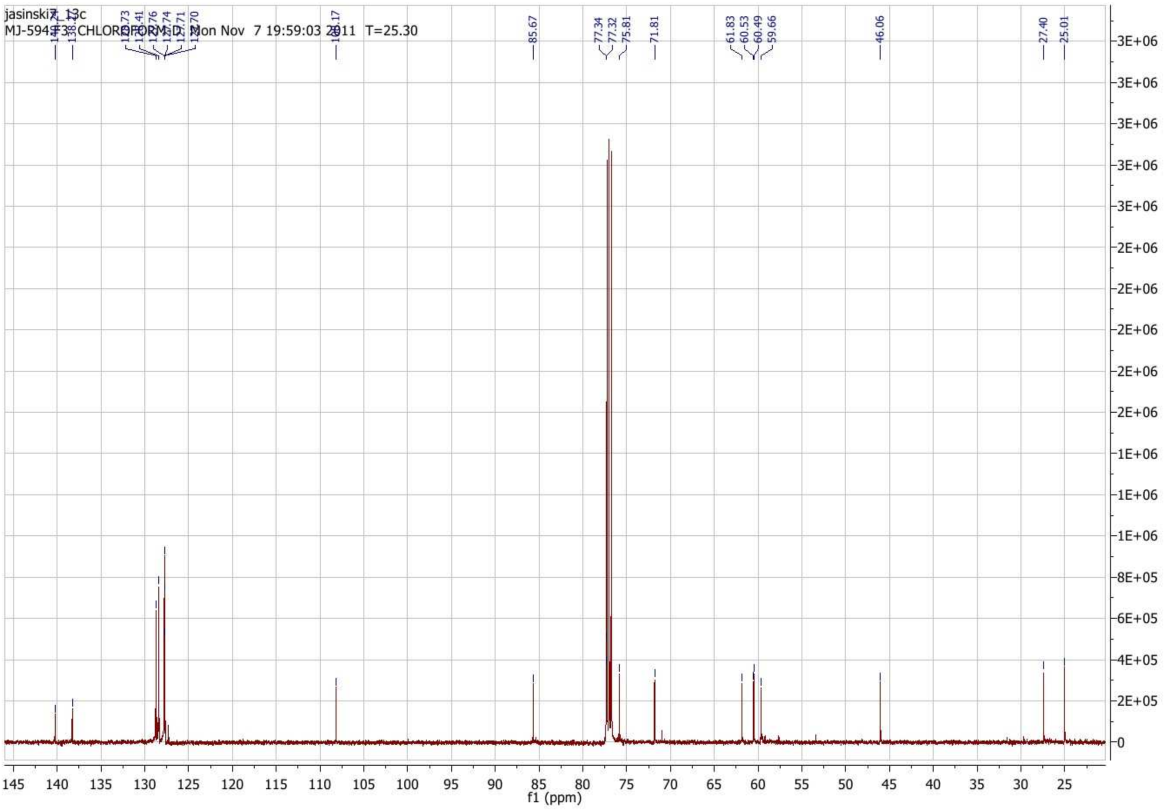
jasinski7_13c
MJ-594.F3 CHLOROFORM-D Mon Nov 7 19:59:03 2011 T=25.30
85.67
77.34
77.32
75.81
71.81
61.83
60.53
60.49
59.66
46.06
27.40
25.01
f1 (ppm)

Compound 21:

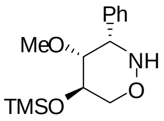
Ph
MeO
NH
O
TMSO

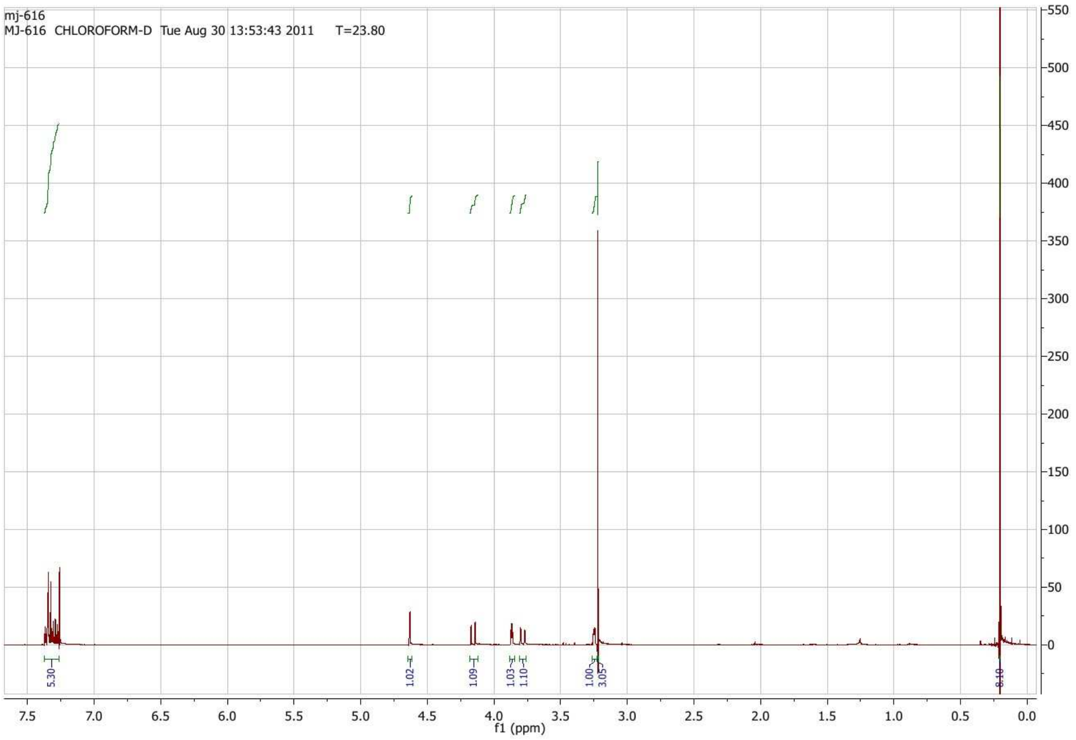
mj-616
MJ-616 CHLOROFORM-D Tue Aug 30 13:53:43 2011 T=23.80
f1 (ppm)

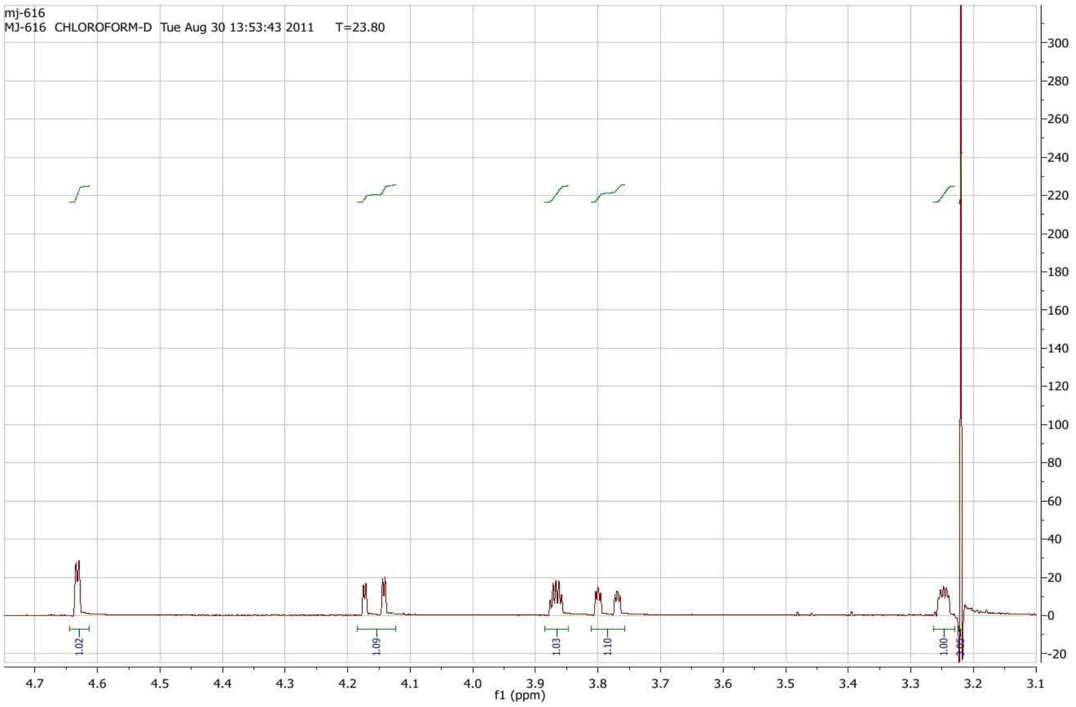
mj-616
MJ-616 CHLOROFORM-D Tue Aug 30 13:53:43 2011 T=23.80
f1 (ppm)

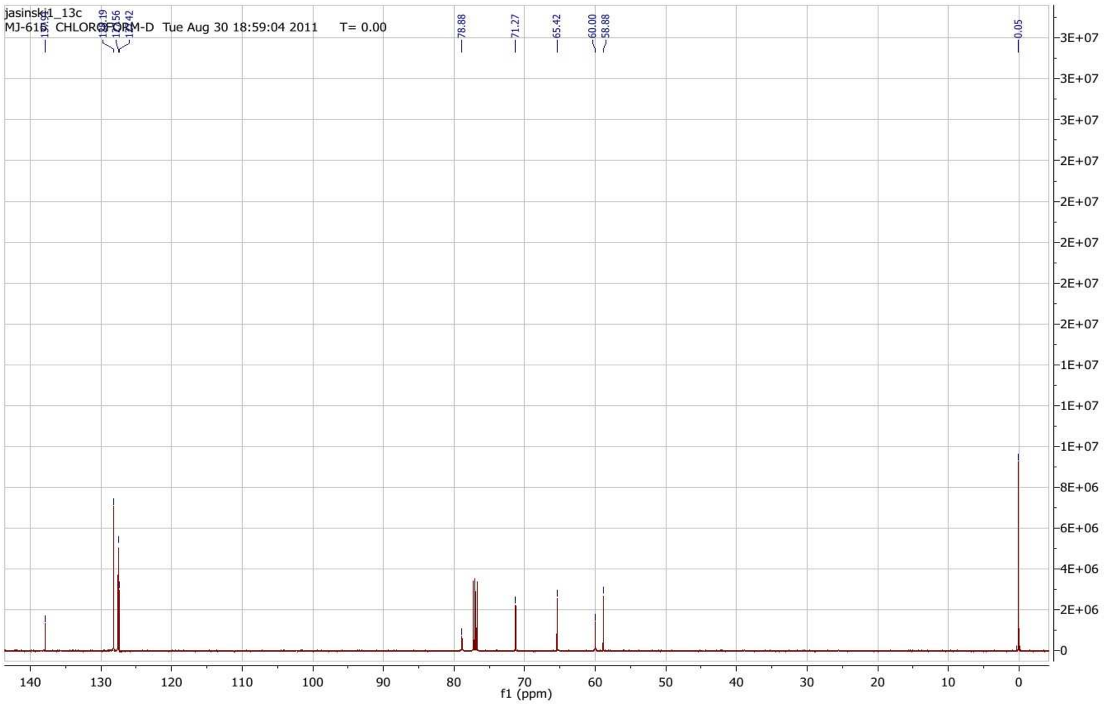
jasinski1_13c
MJ-616 CHLOROFORM-D Tue Aug 30 18:59:04 2011 T= 0.00
78.88
71.27
65.42
60.00
58.88
0.05
f1 (ppm)

Compound 23:

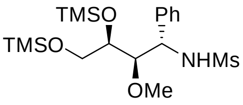
TMSO
Ph
TMSO
NHMs
OMe

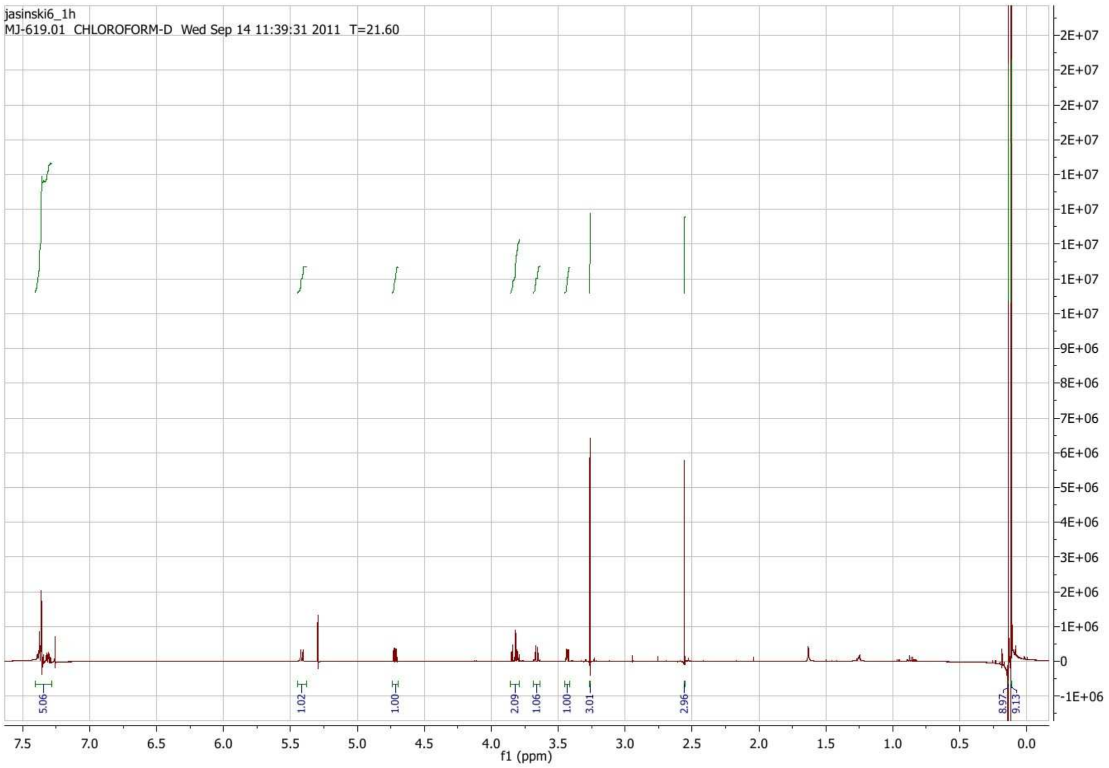
jasinski6_1h
MJ-619.01 CHLOROFORM-D Wed Sep 14 11:39:31 2011 T=21.60
5.06
1.02
1.00
2.09
1.06
1.00
3.01
2.96
8.97
9.13
f1 (ppm)

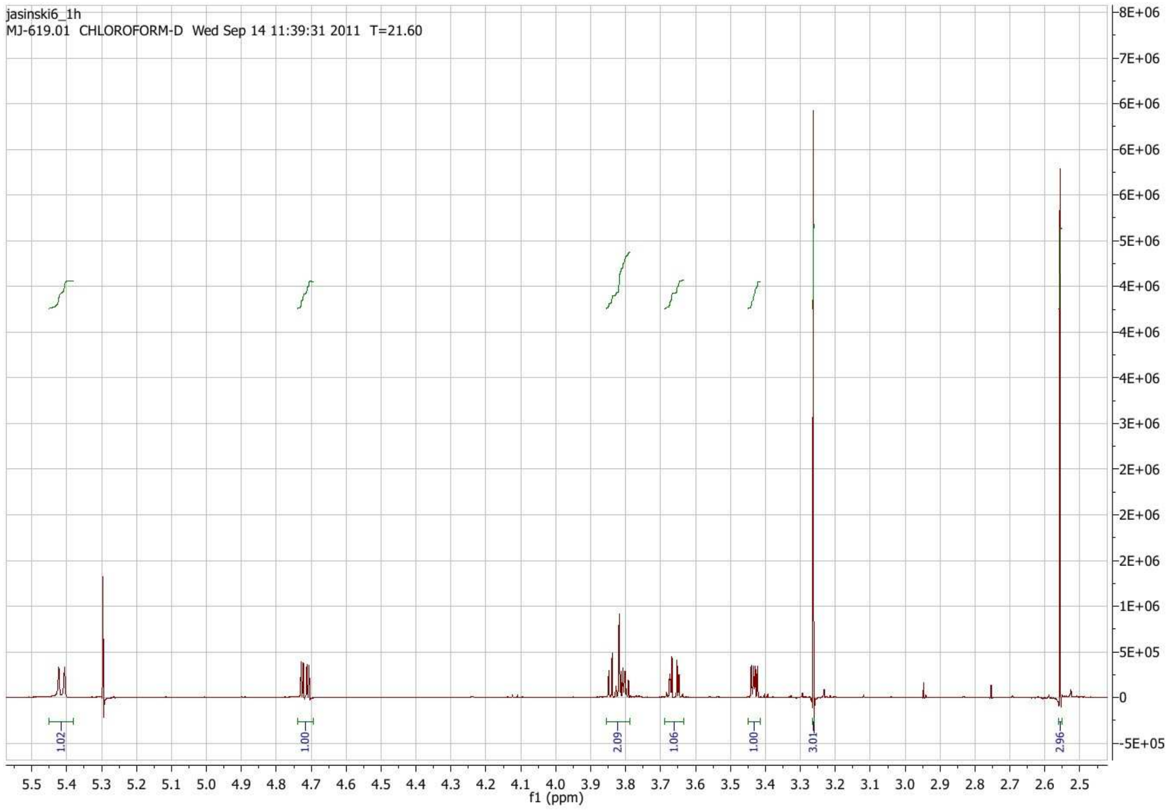
jasinski6_1h
MJ-619.01 CHLOROFORM-D Wed Sep 14 11:39:31 2011 T=21.60
f1 (ppm)

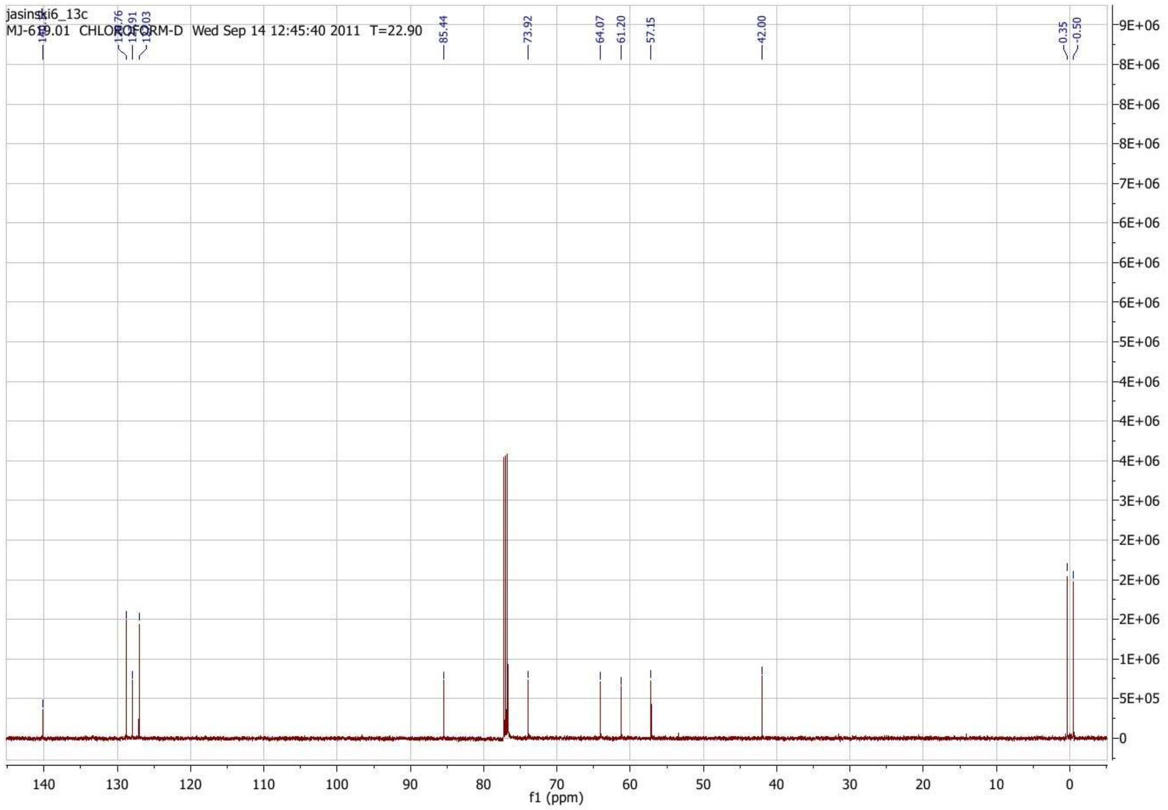
jasinski6_13c
MJ-619.01 CHLOROFORM-D Wed Sep 14 12:45:40 2011 T=22.90
85.44
73.92
64.07
61.20
57.15
42.00
0.35
-0.50
f1 (ppm)

Compound 24:

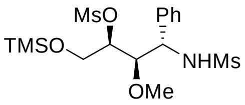
MsO
Ph
TMSO
NHMs
OMe

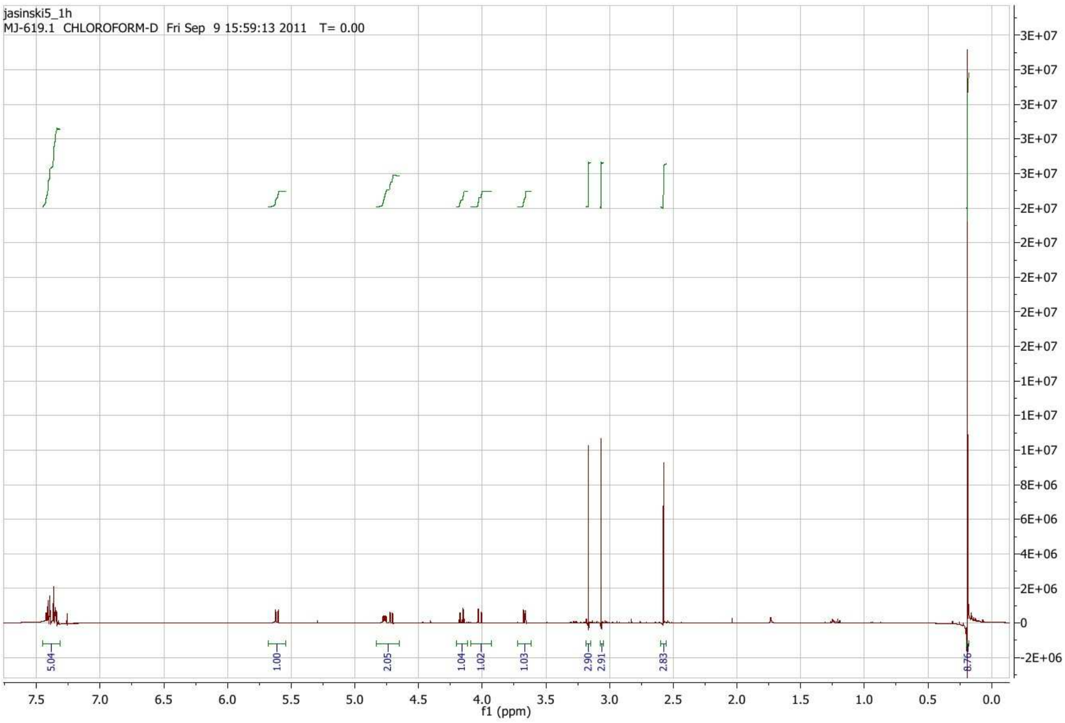
jasinski5_1h
MJ-619.1 CHLOROFORM-D Fri Sep 9 15:59:13 2011 T= 0.00
5.04
1.00
2.05
1.04
1.02
1.03
2.90
2.91
2.83
8.76
f1 (ppm)

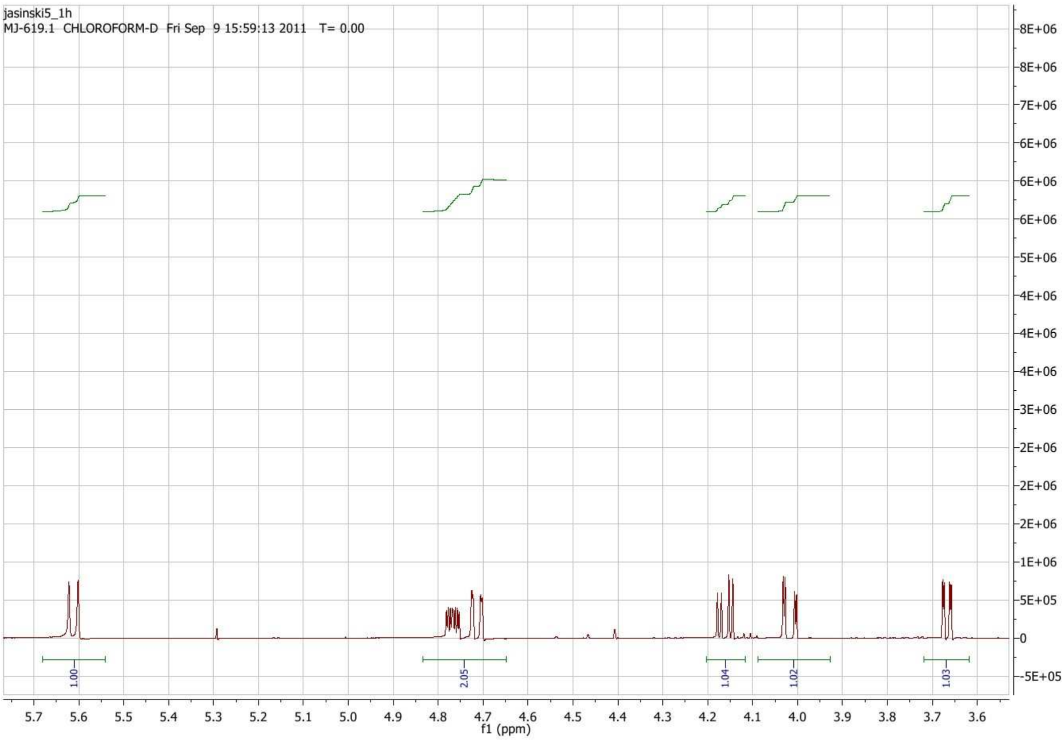
jasinski5_1h
MJ-619.1 CHLOROFORM-D Fri Sep 9 15:59:13 2011 T= 0.00
1.00
2.05
1.04
1.02
1.03
f1 (ppm)

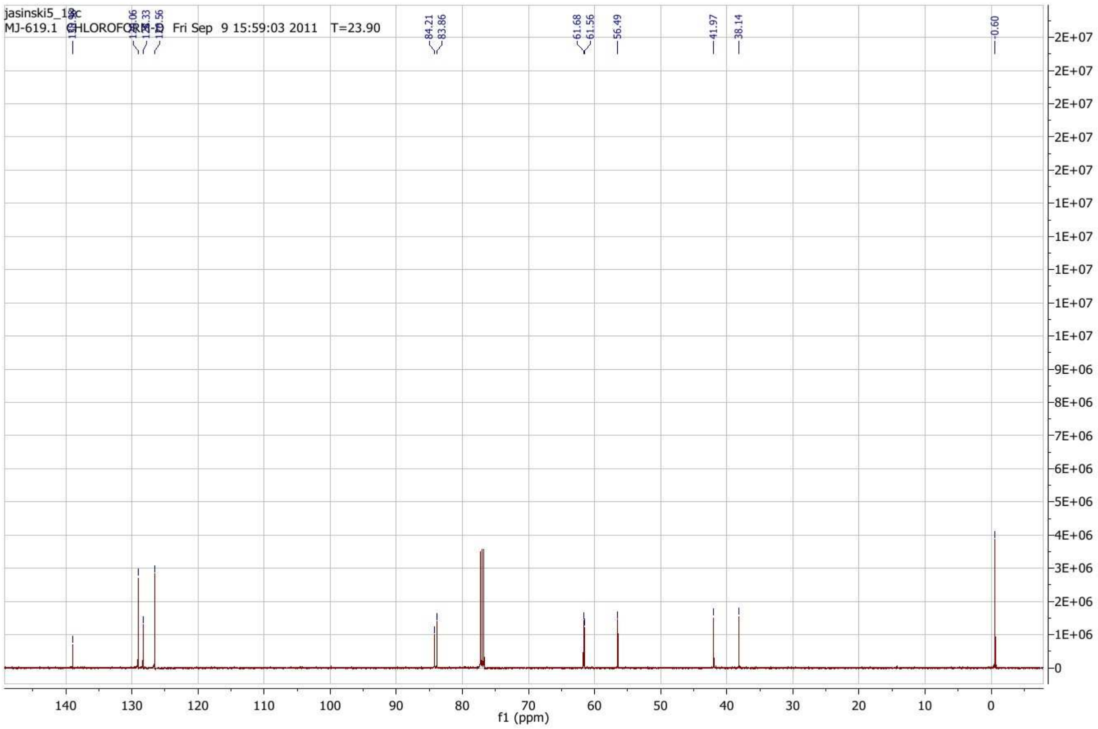
jasinski5_13c
MJ-619.1 CHLOROFORM-D Fri Sep 9 15:59:03 2011 T=23.90
129.06
128.33
126.56
84.21
83.86
61.68
61.56
56.49
41.97
38.14
0.60
f1 (ppm)

Compound 25:

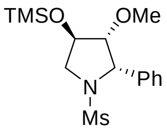
TMSO
OMe
Ph
N
Ms

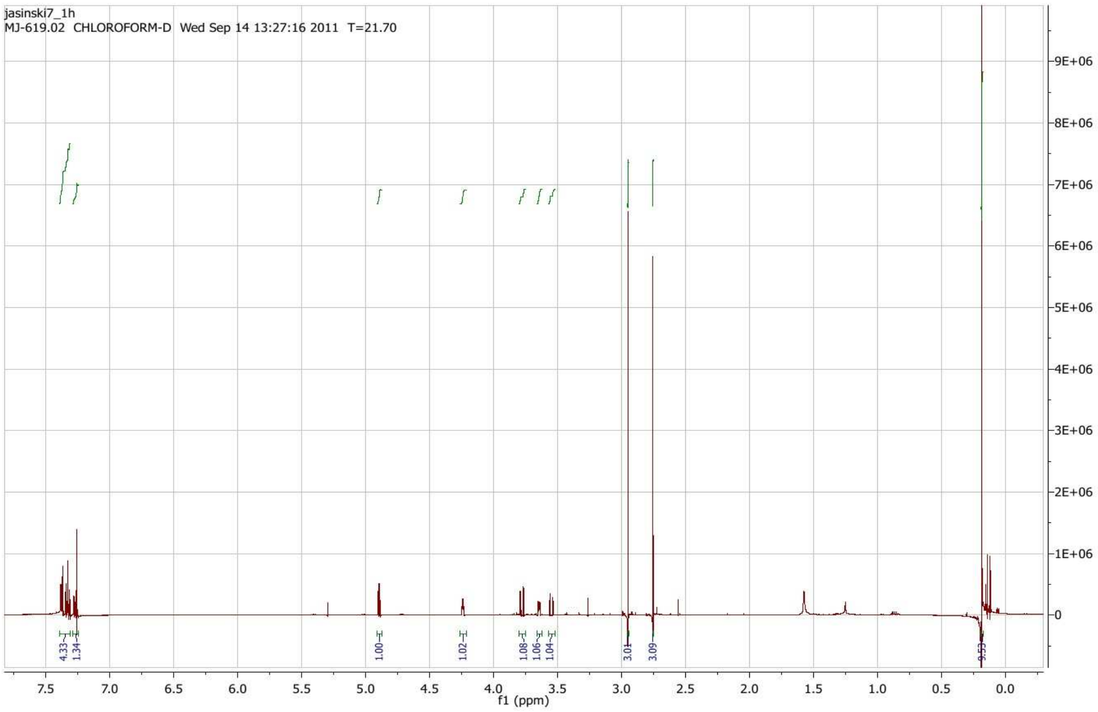
jasinski7_1h
MJ-619.02 CHLOROFORM-D Wed Sep 14 13:27:16 2011 T=21.70
4.33
1.34
1.00
1.02
1.08
1.06
1.04
3.01
3.09
f1 (ppm)

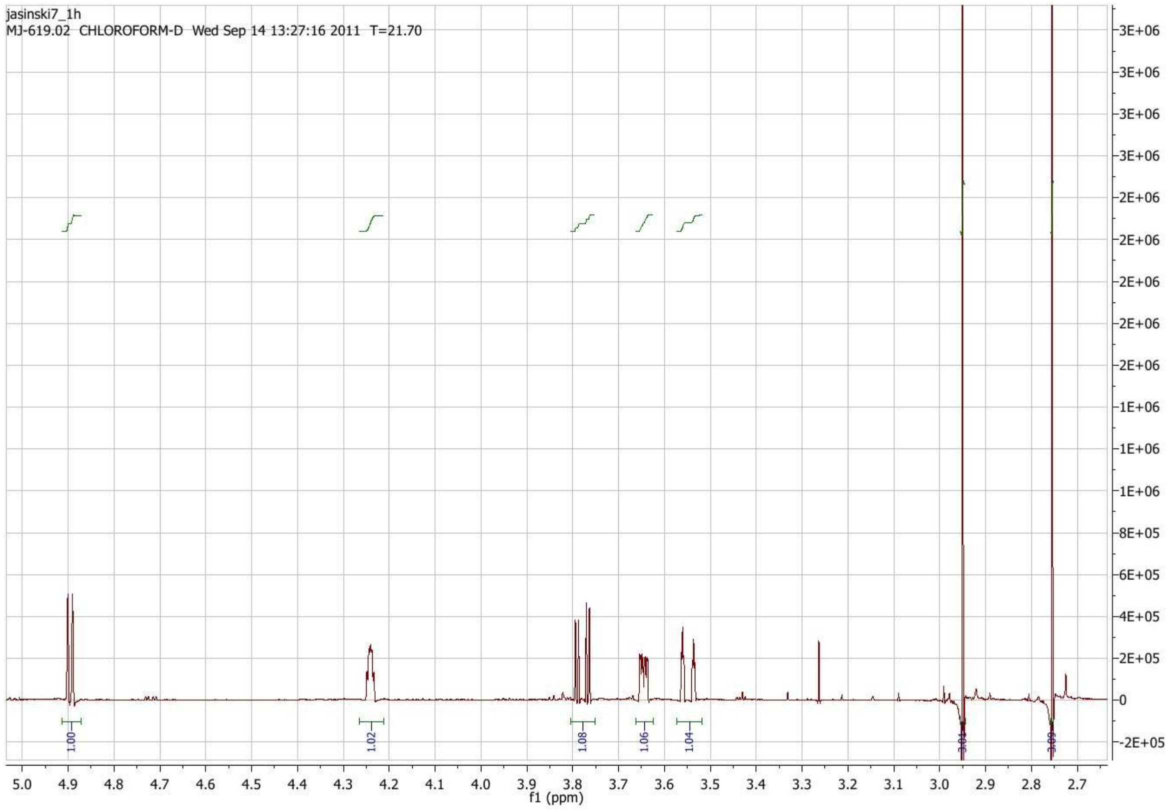
jasinski7_1h
MJ-619.02 CHLOROFORM-D Wed Sep 14 13:27:16 2011 T=21.70
1.00
1.02
1.08
1.06
1.04
3.01
3.09
f1 (ppm)

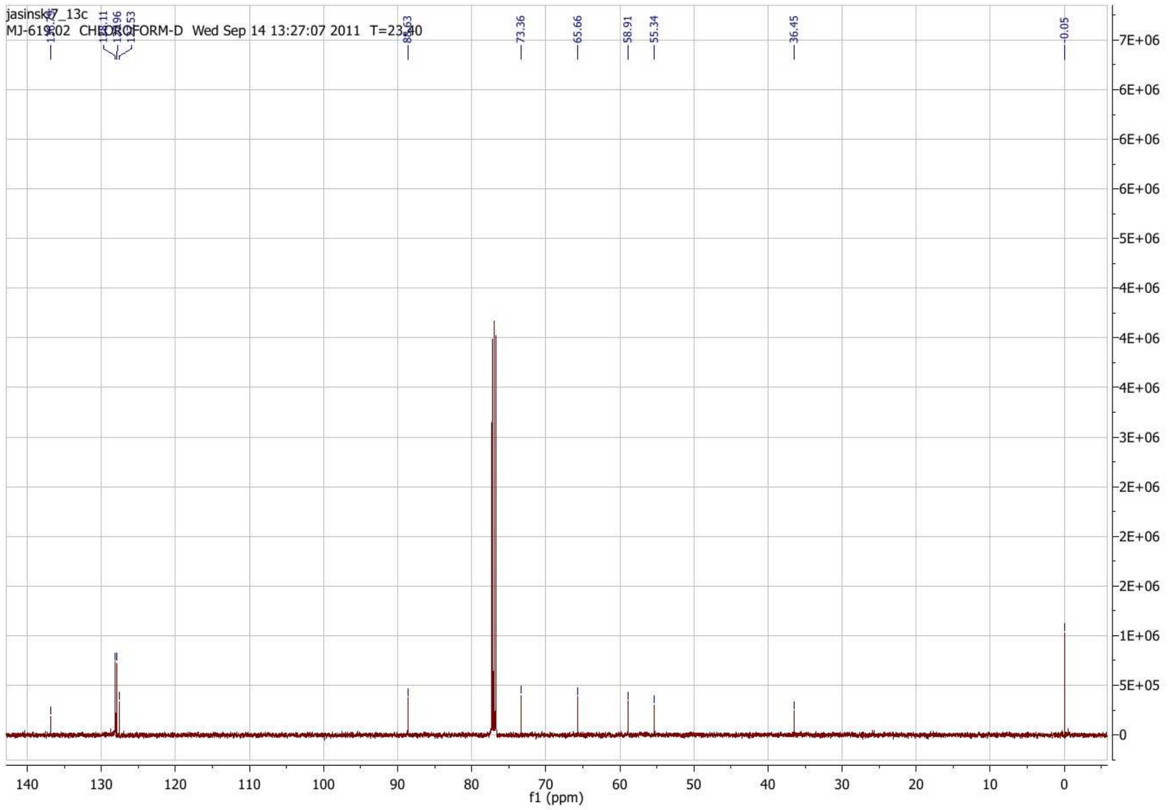
jasinski7_13c
MJ-619.02 CHLOROFORM-D Wed Sep 14 13:27:07 2011 T=23.40
73.36
65.66
58.91
55.34
36.45
0.05
f1 (ppm)

Compound 26:

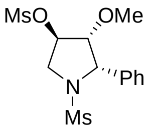

MsO
OMe
Ph
N
Ms

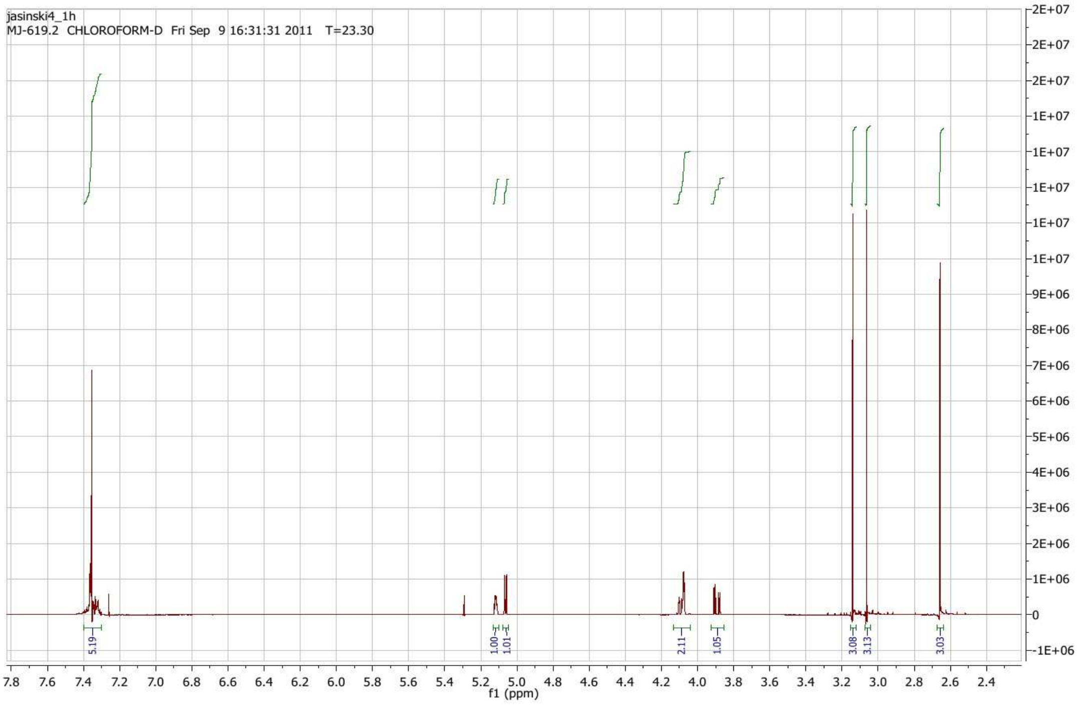

jasinski4_1h
MJ-619.2 CHLOROFORM-D Fri Sep 9 16:31:31 2011 T=23.30
5.19
1.00
1.01
2.11
1.05
3.08
3.13
3.03
f1 (ppm)

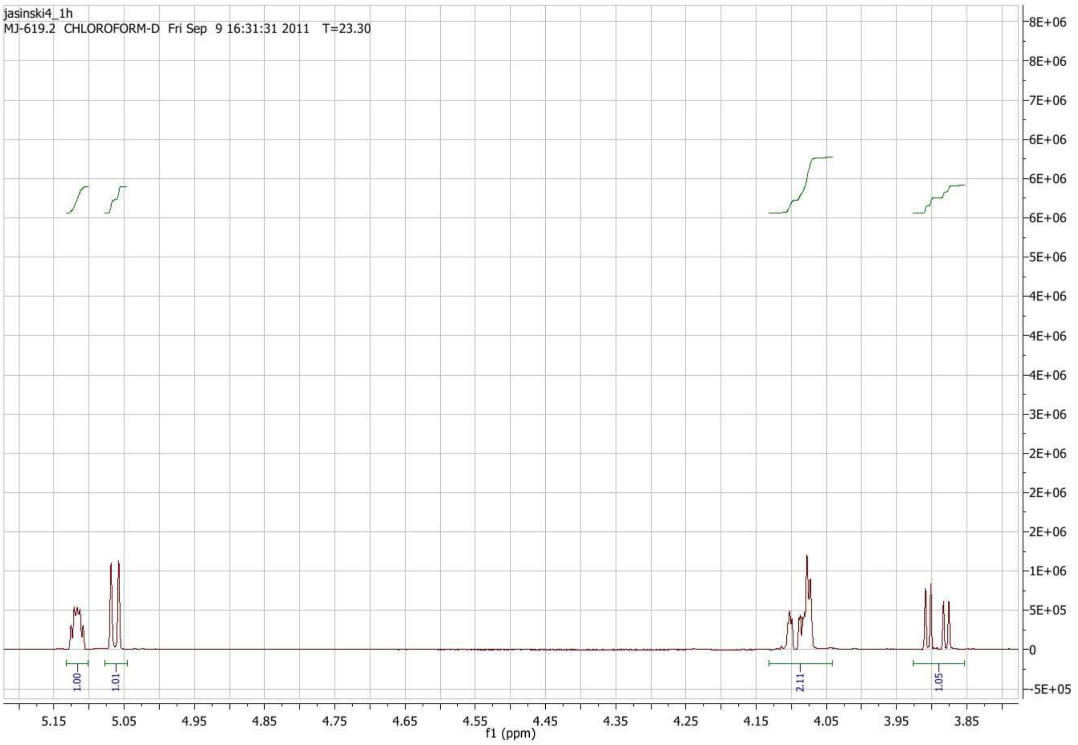

jasinski4_1h
MJ-619.2 CHLOROFORM-D Fri Sep 9 16:31:31 2011 T=23.30
1.00
1.01
2.11
1.05
f1 (ppm)

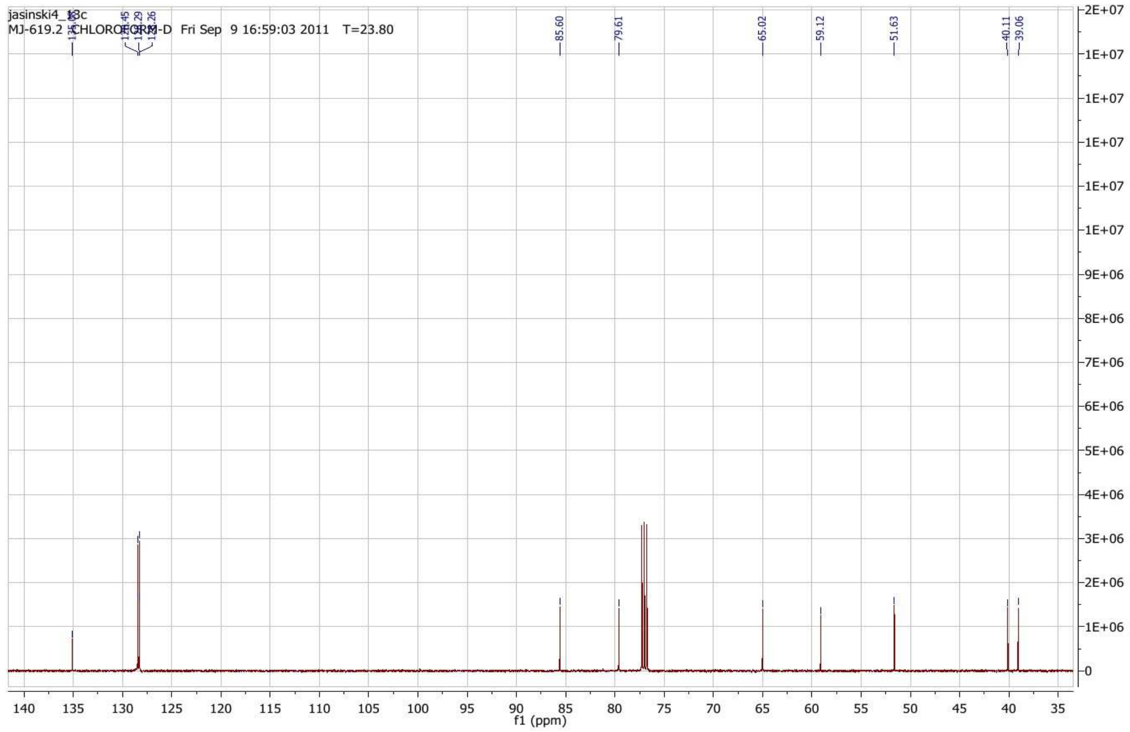
jasinski4_13c
MJ-619.2 CHLOROFORM-D Fri Sep 9 16:59:03 2011 T=23.80
85.60
79.61
65.02
59.12
51.63
40.11
39.06
f1 (ppm)

Compound 27:

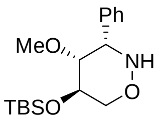
Ph
MeO
NH
O
TBSO

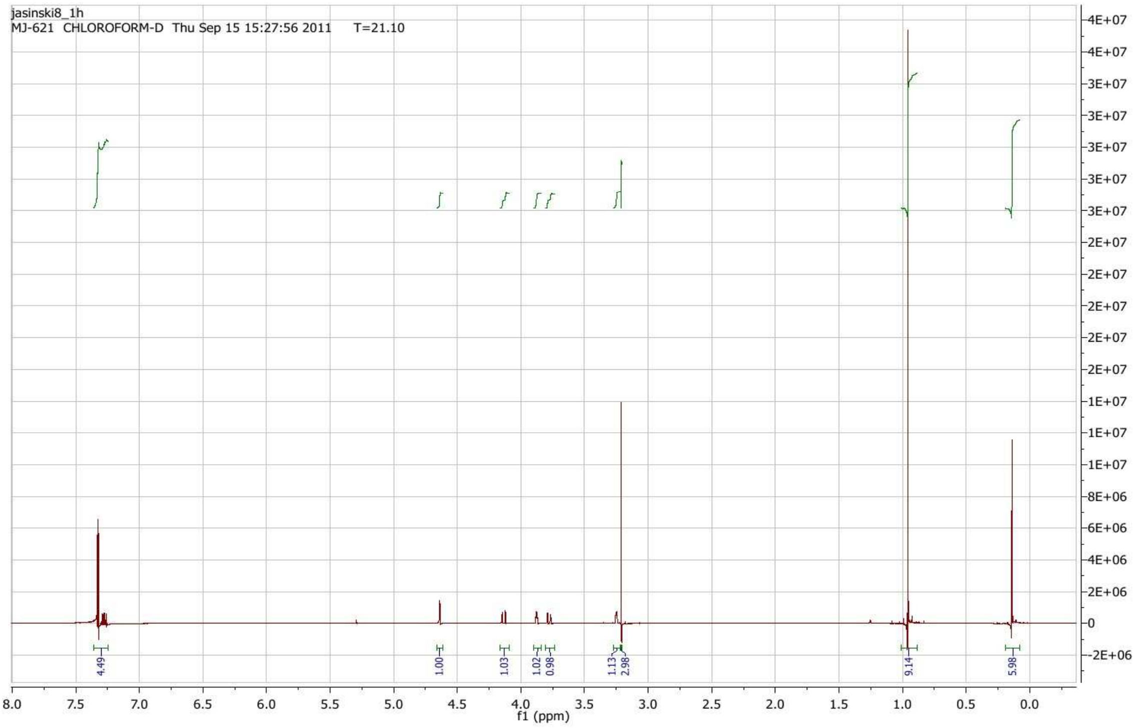
jasinski8_1h
MJ-621 CHLOROFORM-D Thu Sep 15 15:27:56 2011 T=21.10
4.49
1.00
1.03
1.02
0.98
1.13
2.98
9.14
5.98
f1 (ppm)

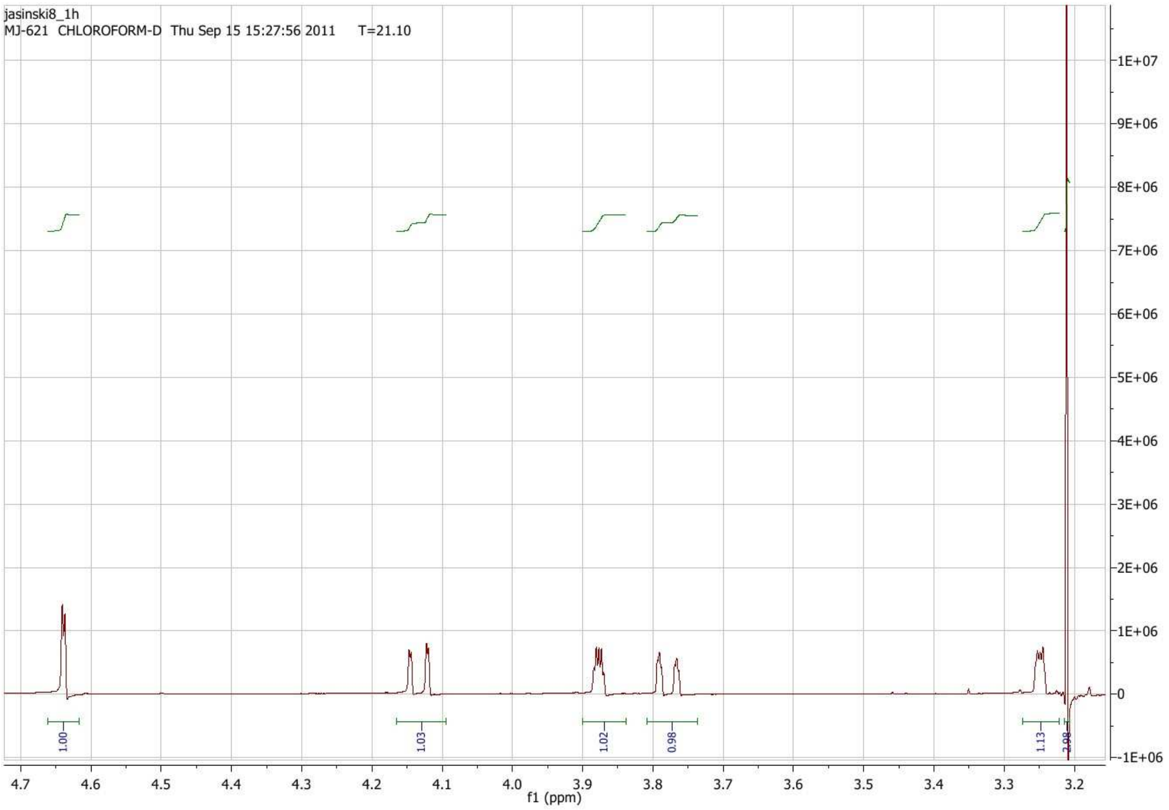
jasinski8_1h
MJ-621 CHLOROFORM-D Thu Sep 15 15:27:56 2011 T=21.10
1.00
1.03
1.02
0.98
1.13
f1 (ppm)

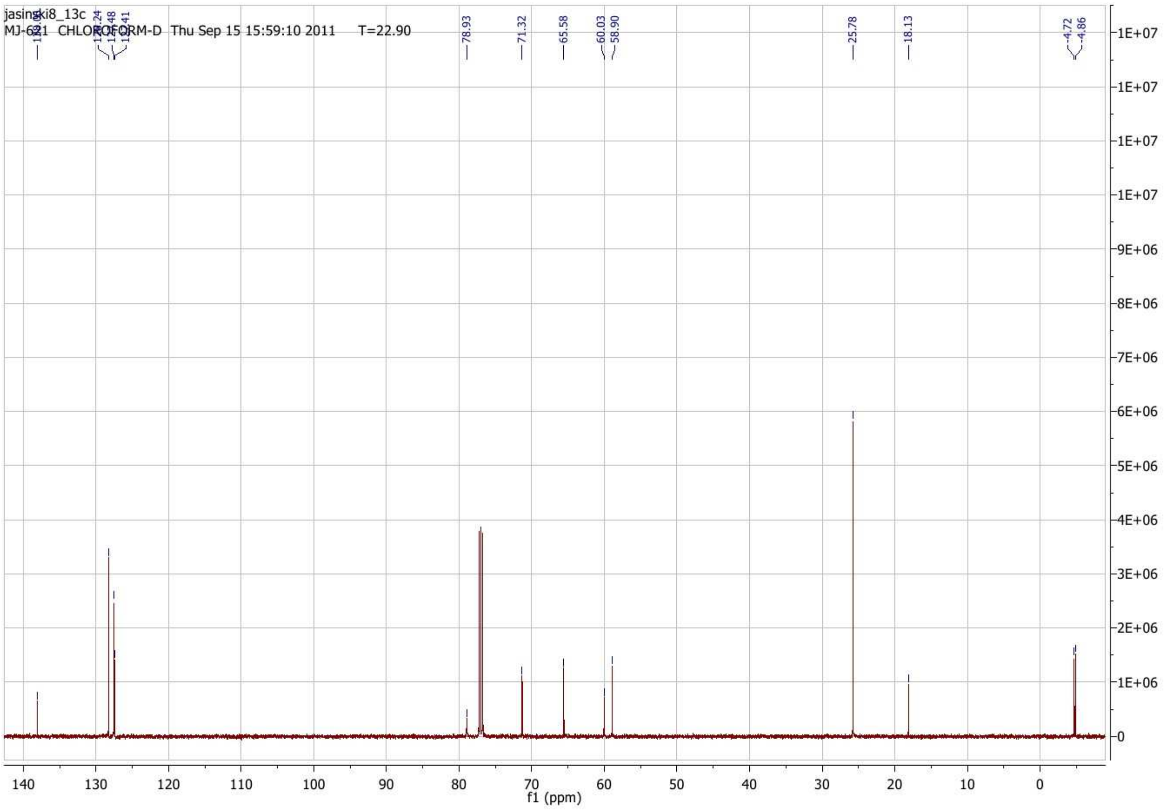
jasinski8_13c
MJ-621 CHLOROFORM-D Thu Sep 15 15:59:10 2011 T=22.90
78.93
71.32
65.58
60.03
58.90
25.78
18.13
-4.72
-4.86
f1 (ppm)

Compound 28:

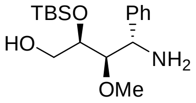
TBSO
Ph
HO
NH2
OMe

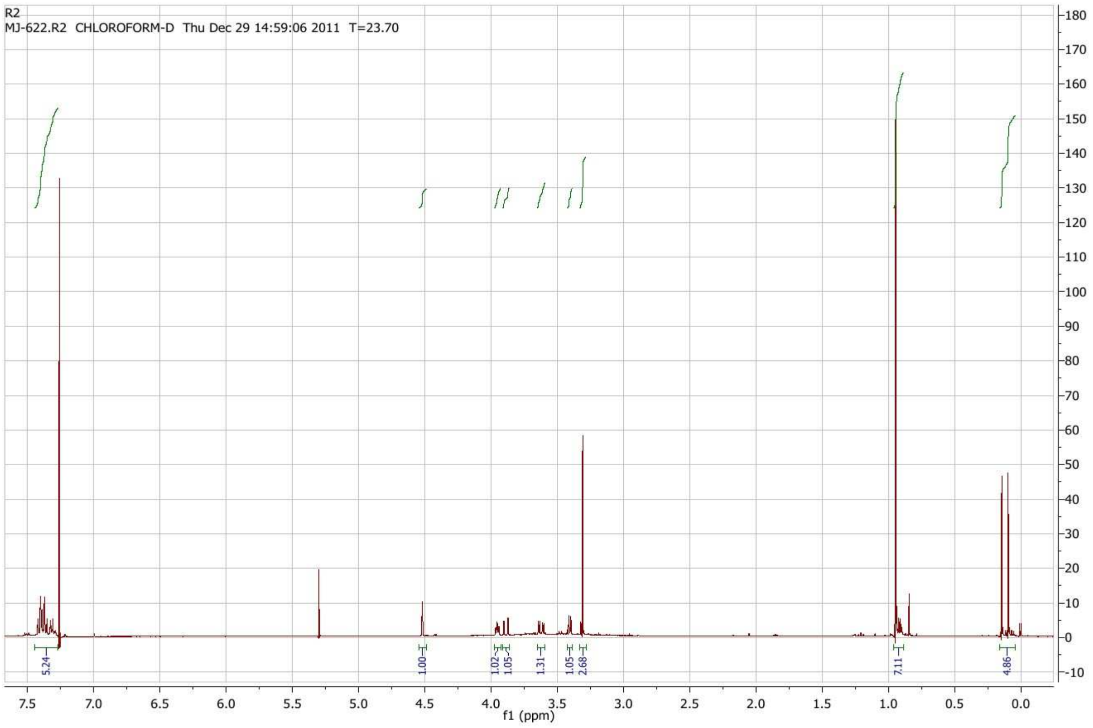
R2
MJ-622.R2 CHLOROFORM-D Thu Dec 29 14:59:06 2011 T=23.70
5.24
1.00
1.02
1.05
1.31
1.05
2.68
7.11
4.86
f1 (ppm)

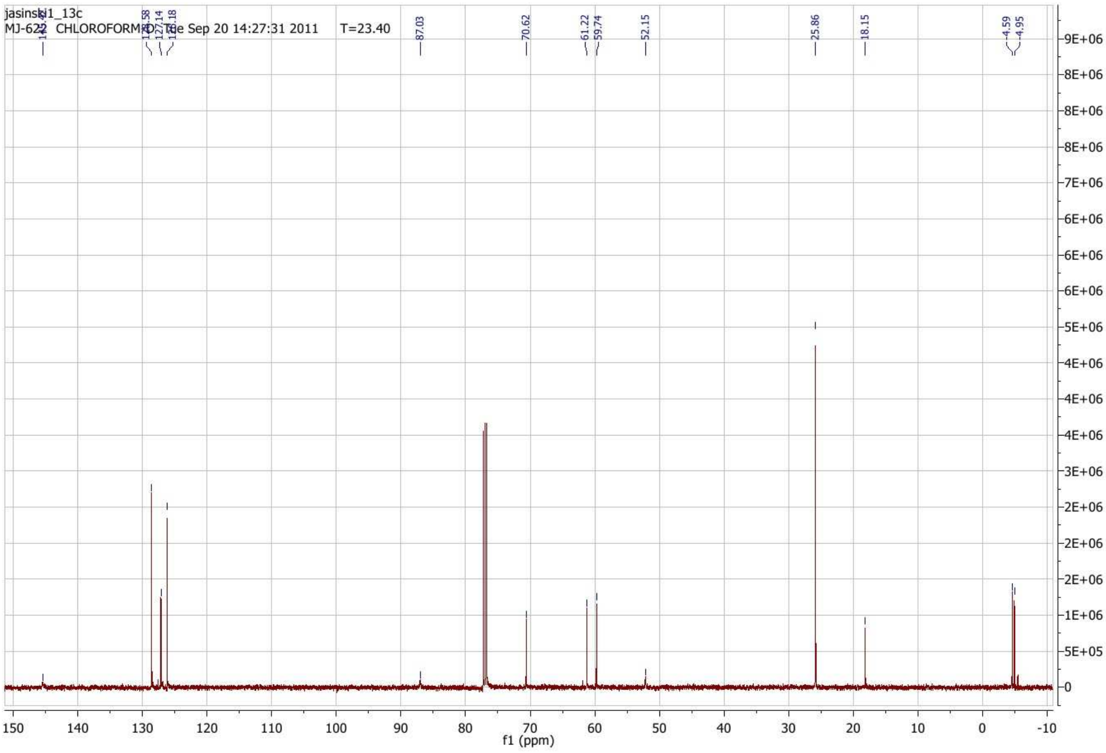
MJ-622 CHLOROFORM-D Tue Sep 20 14:27:31 2011 T=23.40
127.14
126.18
87.03
70.62
61.22
59.74
52.15
25.86
18.15
-4.59
-4.95
f1 (ppm)

Compound 29:

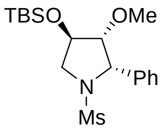
TBSO
OMe
N
Ph
Ms

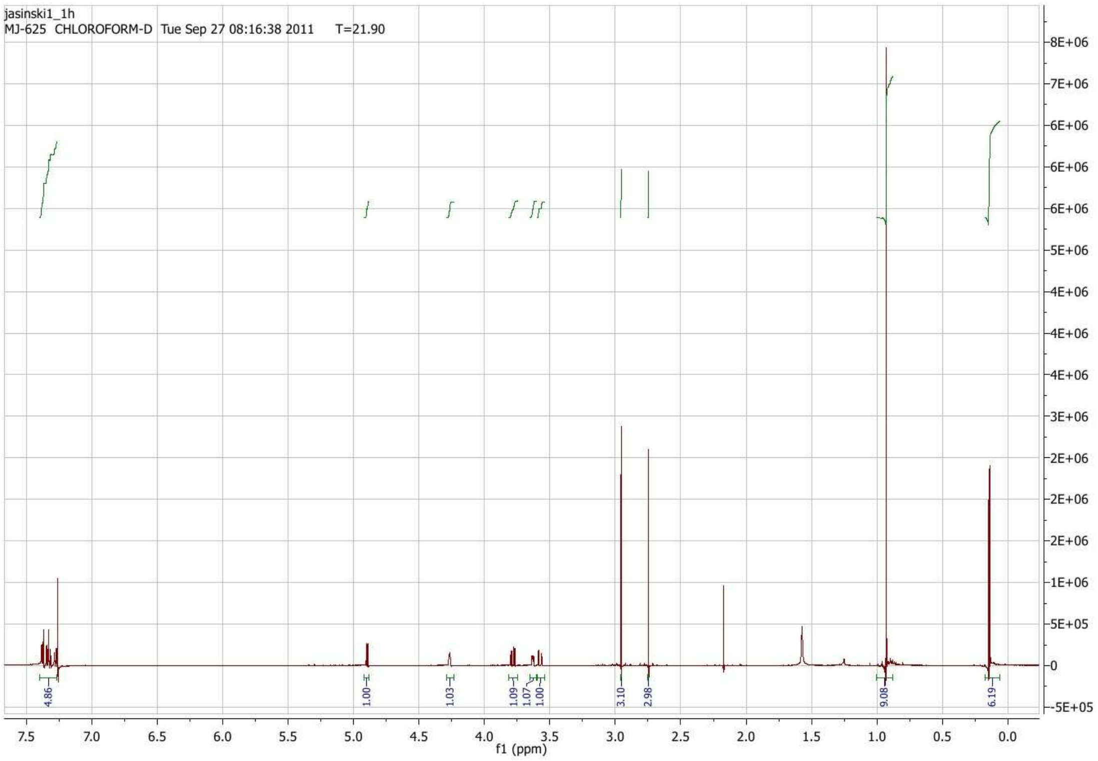
jasinski1_1h
MJ-625 CHLOROFORM-D Tue Sep 27 08:16:38 2011 T=21.90
f1 (ppm)

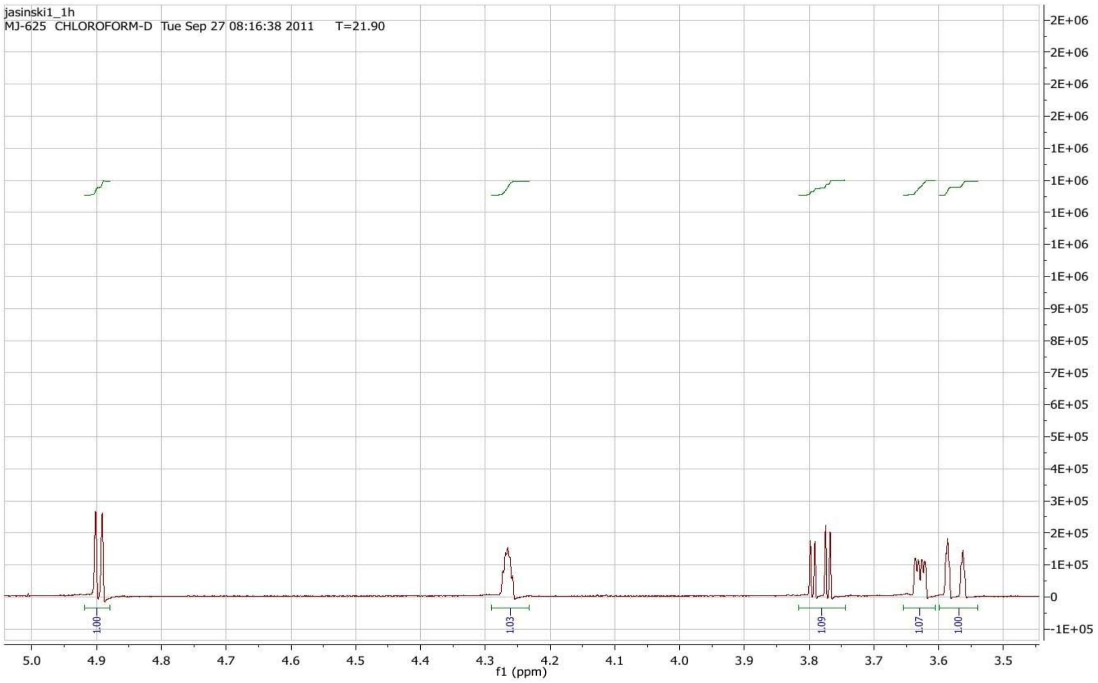
jasinski1_1h
MJ-625 CHLOROFORM-D Tue Sep 27 08:16:38 2011 T=21.90
f1 (ppm)

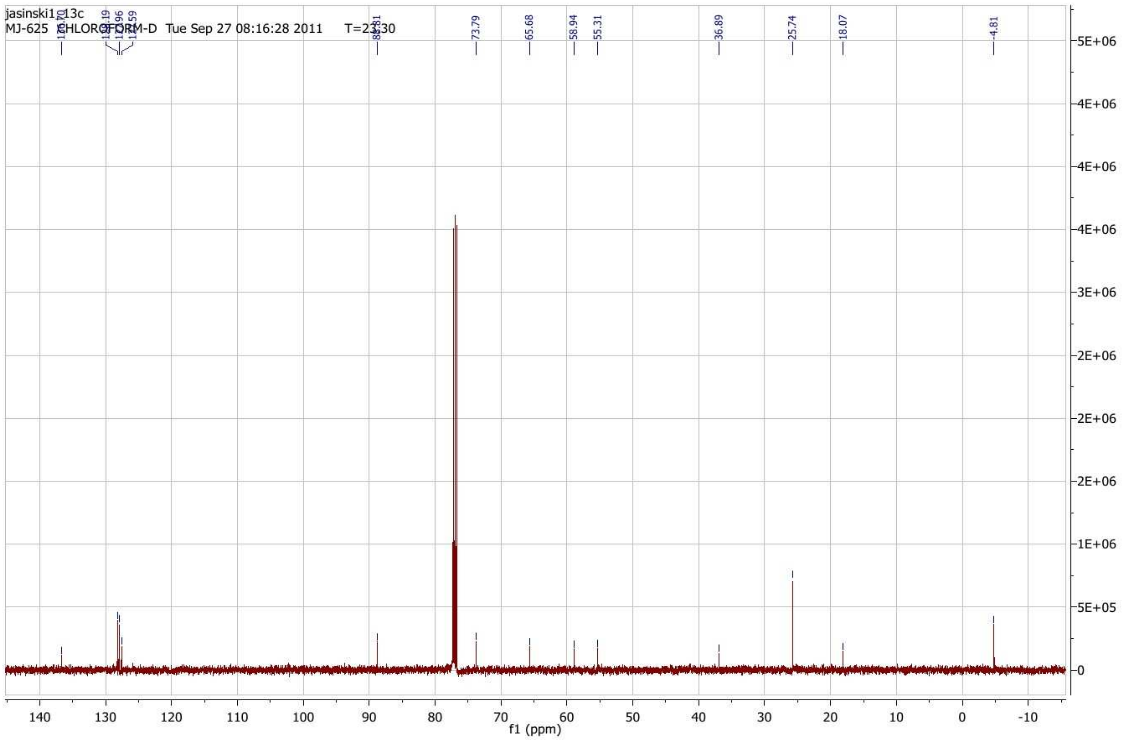
jasinski1_13c
MJ-625 CHLOROFORM-D Tue Sep 27 08:16:28 2011 T=23.30
73.79
65.68
58.94
55.31
36.89
25.74
18.07
-4.81
f1 (ppm)

Compound 30:

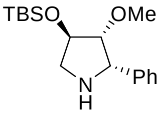
TBSO
OMe
Ph
N
H

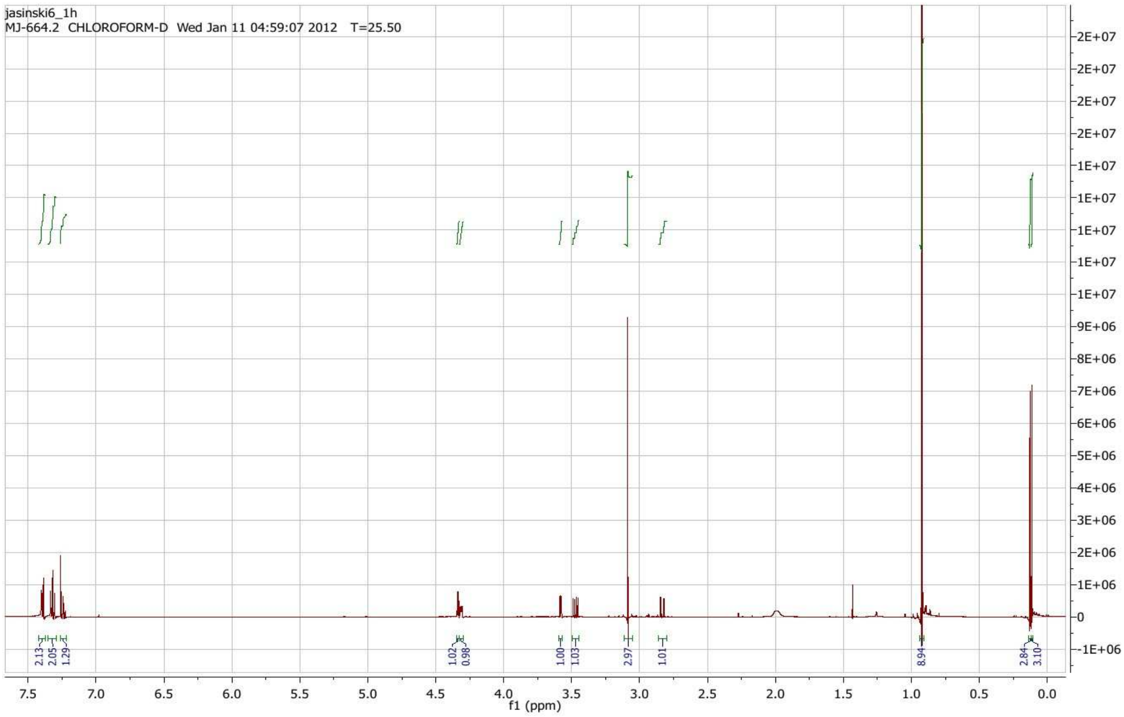
jasinski6_1h
MJ-664.2 CHLOROFORM-D Wed Jan 11 04:59:07 2012 T=25.50
2.13
2.05
1.29
1.02
0.98
1.00
1.03
2.97
1.01
8.94
2.84
3.10
f1 (ppm)

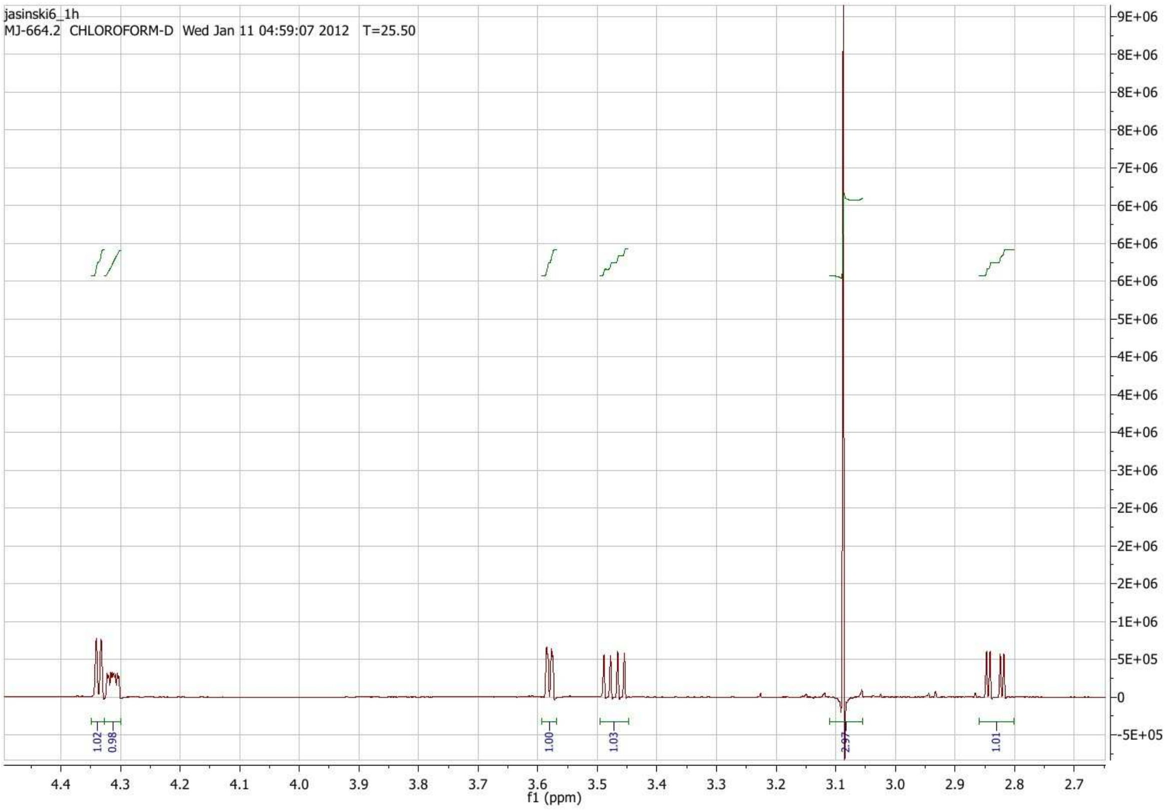

jasinski6_1h
MJ-664.2 CHLOROFORM-D Wed Jan 11 04:59:07 2012 T=25.50
f1 (ppm)

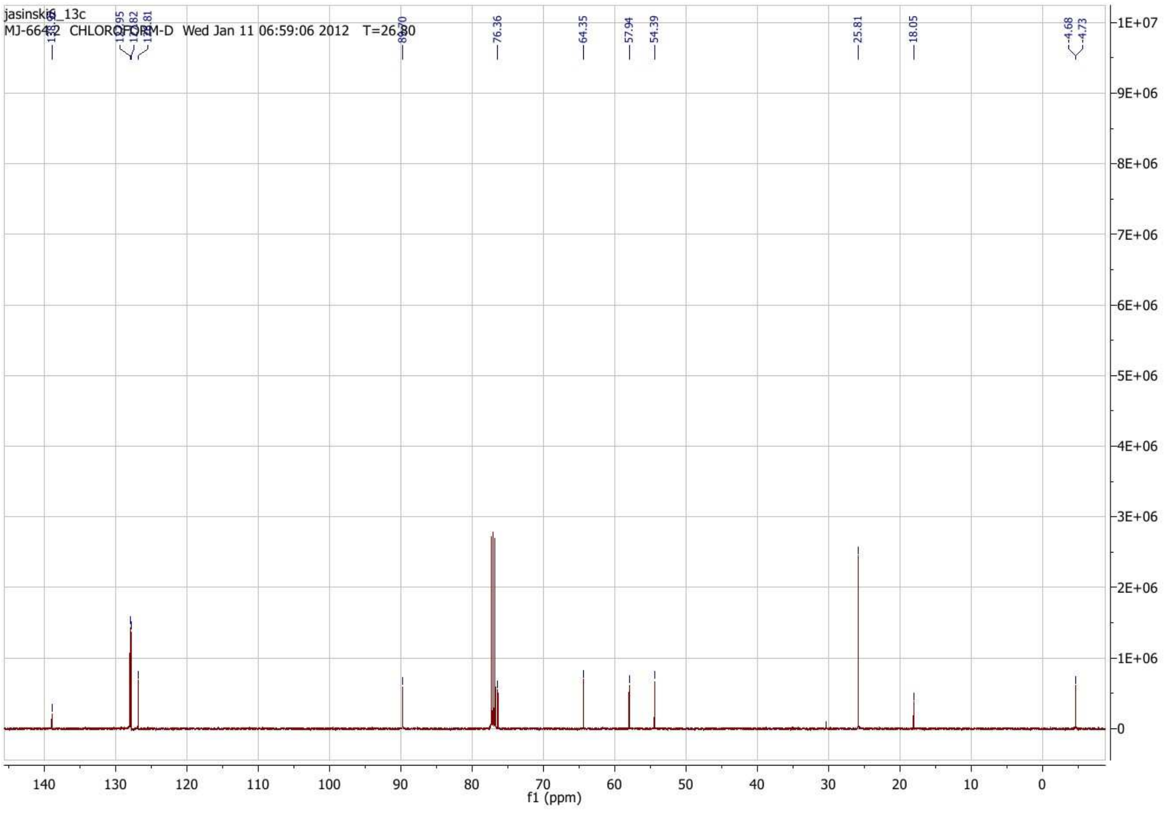

jasinski6_13c
MJ-664.2 CHLOROFORM-D Wed Jan 11 06:59:06 2012 T=26.30
76.36
64.35
57.94
54.39
25.81
18.05
f1 (ppm)

Compound 31:

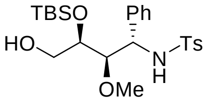
TBSO
Ph
HO
N
Ts
H
OMe

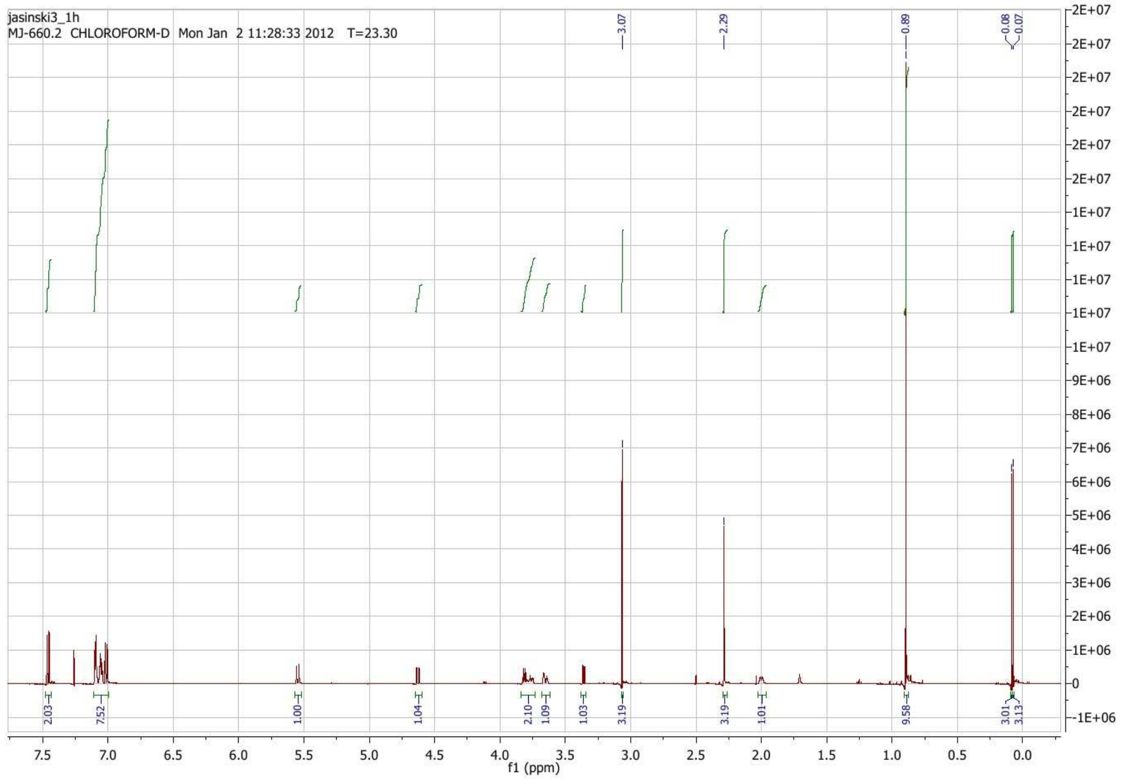
jasinski3_1h
MJ-660.2 CHLOROFORM-D Mon Jan 2 11:28:33 2012 T=23.30
f1 (ppm)

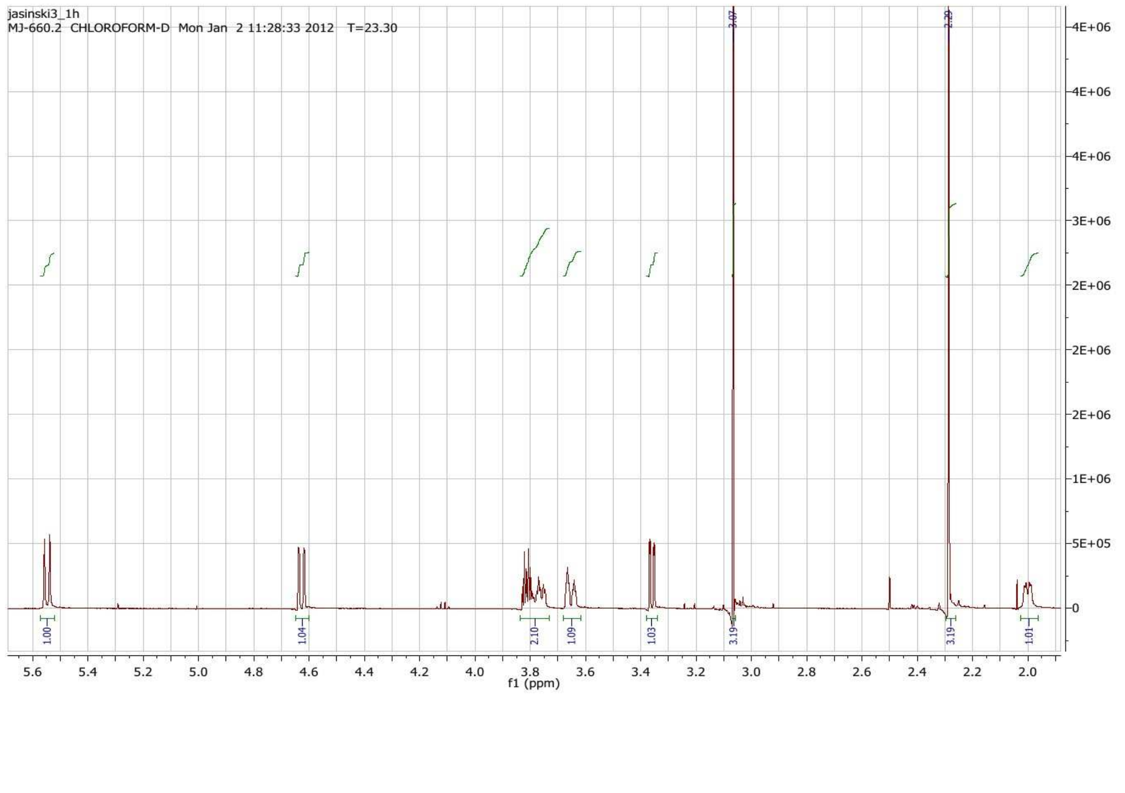
jasinski3_1h
MJ-660.2 CHLOROFORM-D Mon Jan 2 11:28:33 2012 T=23.30
f1 (ppm)

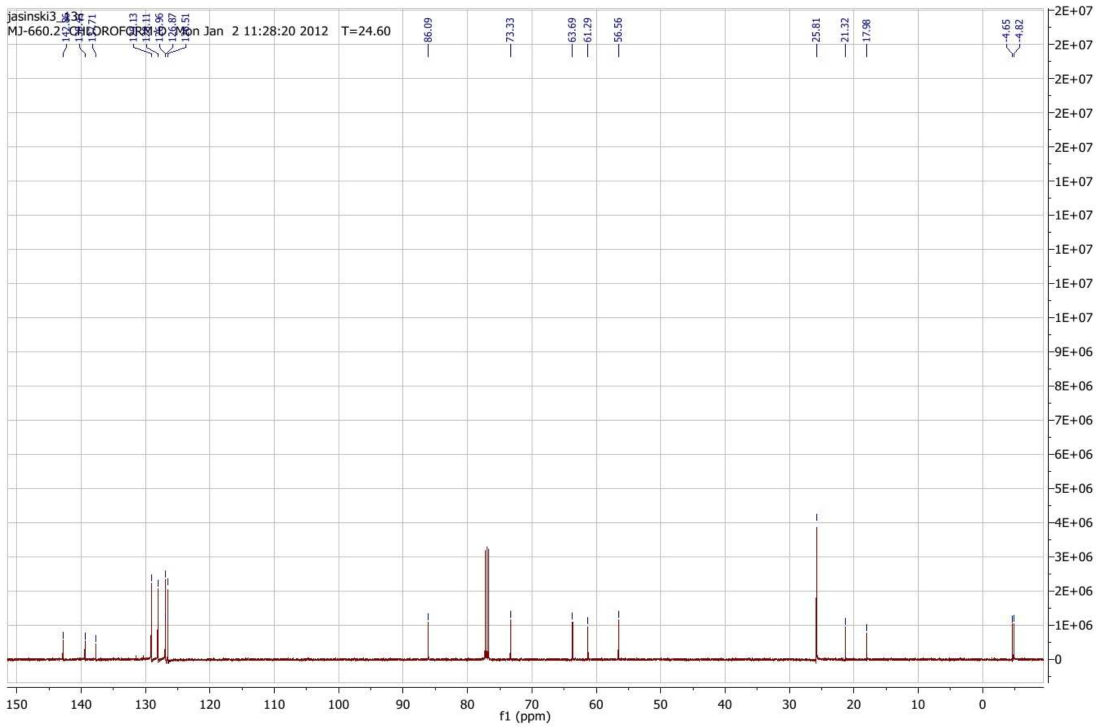
jasinski3_13c
MJ-660.2 CHLOROFORM-D Mon Jan 2 11:28:20 2012 T=24.60
86.09
73.33
63.69
61.29
56.56
25.81
21.32
17.98
4.65
4.82
f1 (ppm)

Compound 32:

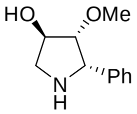
HO
OMe
Ph
N
H

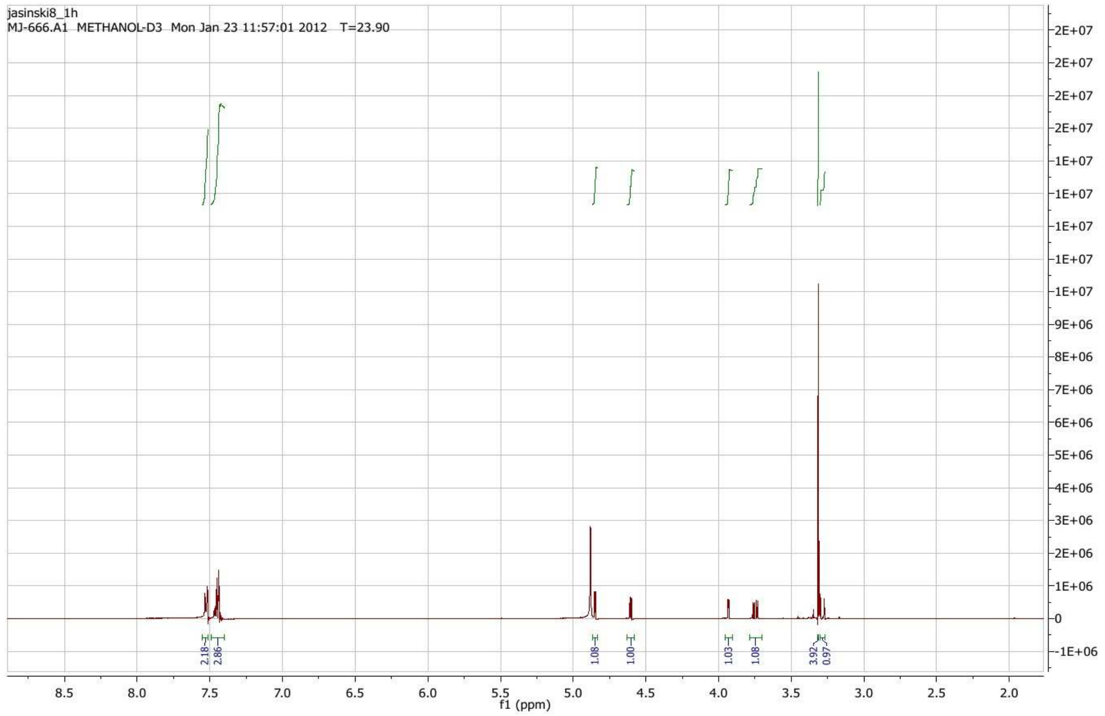
jasinski8_1h
MJ-666.A1 METHANOL-D3 Mon Jan 23 11:57:01 2012 T=23.90
2.18
2.86
1.08
1.00
1.03
1.08
3.92
0.97
f1 (ppm)

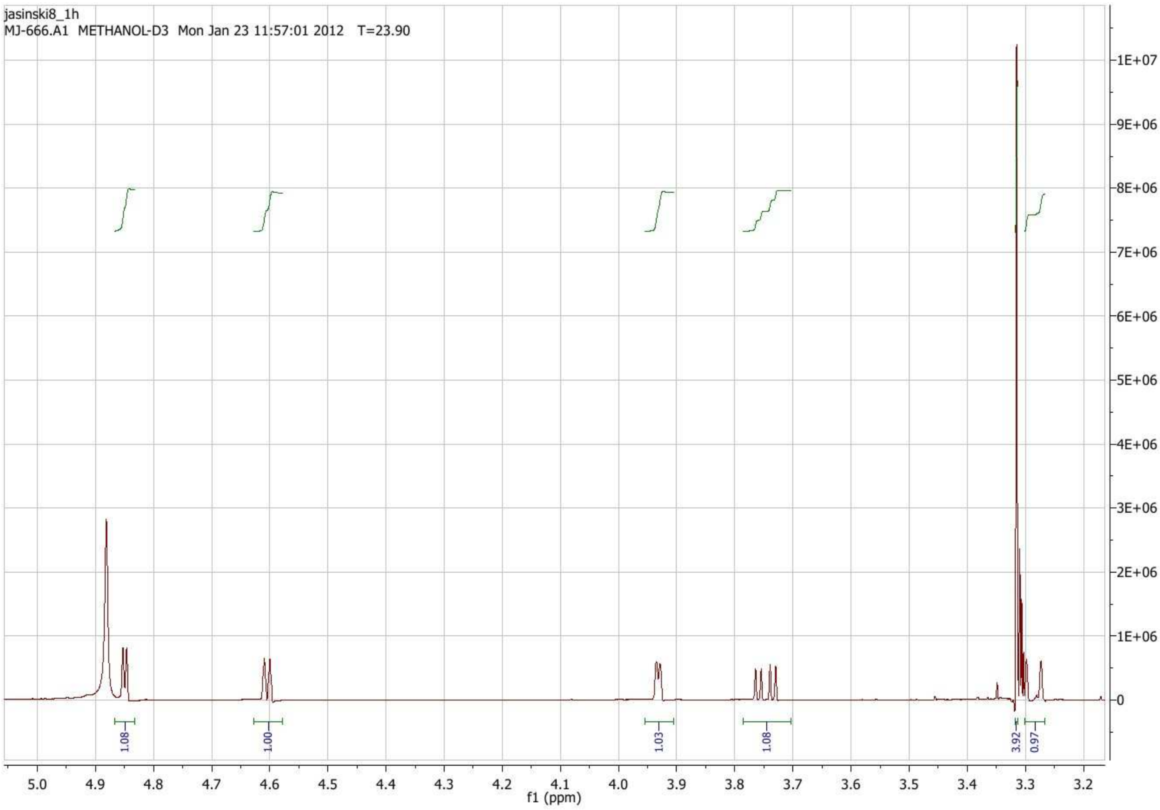
jasinski8_1h
MJ-666.A1 METHANOL-D3 Mon Jan 23 11:57:01 2012 T=23.90
1.08
1.00
1.03
1.08
3.92
0.97
5.0 4.9 4.8 4.7 4.6 4.5 4.4 4.3 4.2 4.1 4.0 3.9 3.8 3.7 3.6 3.5 3.4 3.3 3.2
f1 (ppm)
1E+07
9E+06
8E+06
7E+06
6E+06
5E+06
4E+06
3E+06
2E+06
1E+06
0

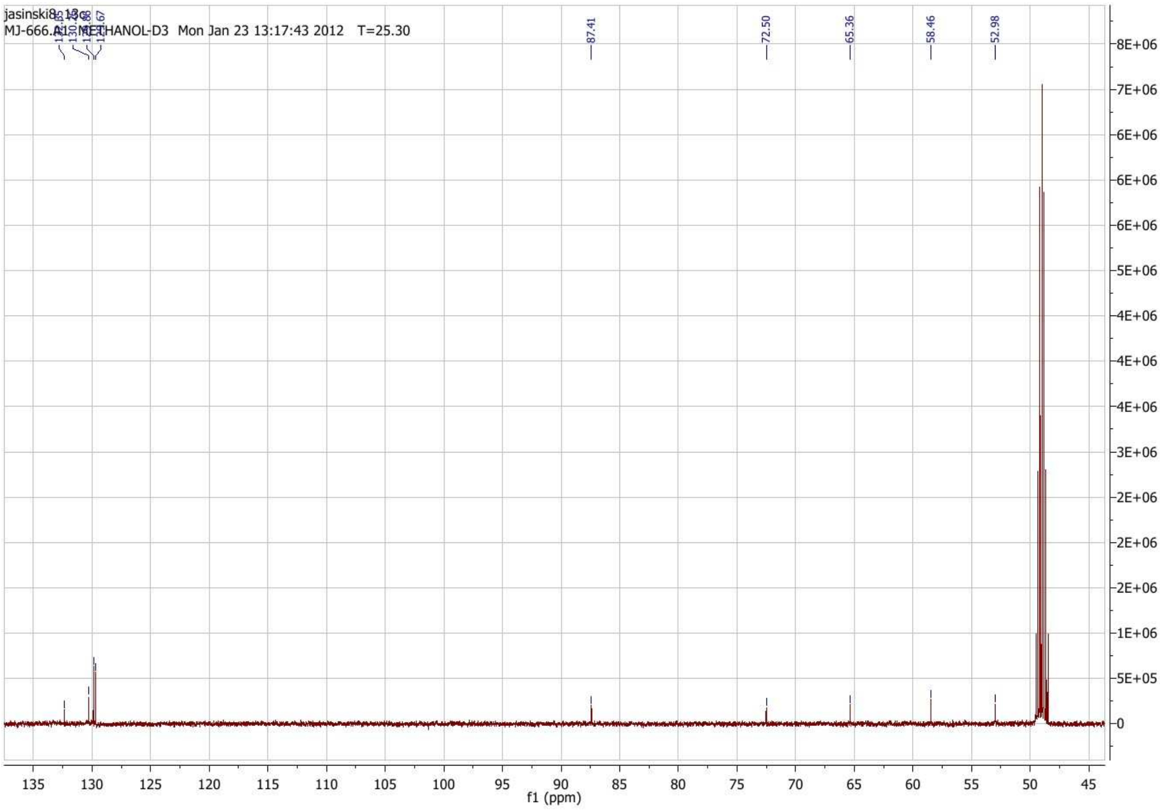
jasinski8_13c
MJ-666.A1 METHANOL-D3 Mon Jan 23 13:17:43 2012 T=25.30
129.67
87.41
72.50
65.36
58.46
52.98
135 130 125 120 115 110 105 100 95 90 85 80 75 70 65 60 55 50 45
f1 (ppm)
8E+06
7E+06
6E+06
6E+06
6E+06
5E+06
4E+06
4E+06
4E+06
3E+06
2E+06
2E+06
2E+06
1E+06
5E+05
0
